# Supplementary figures and images for: Phase separation of a plant virus movement protein and cellular factors support virus-host interactions
Source: PLoS Pathog. 2021 Sep 20;17(9):e1009622. doi: 10.1371/journal.ppat.1009622 (PMC8483311; doi:10.1371/journal.ppat.1009622)

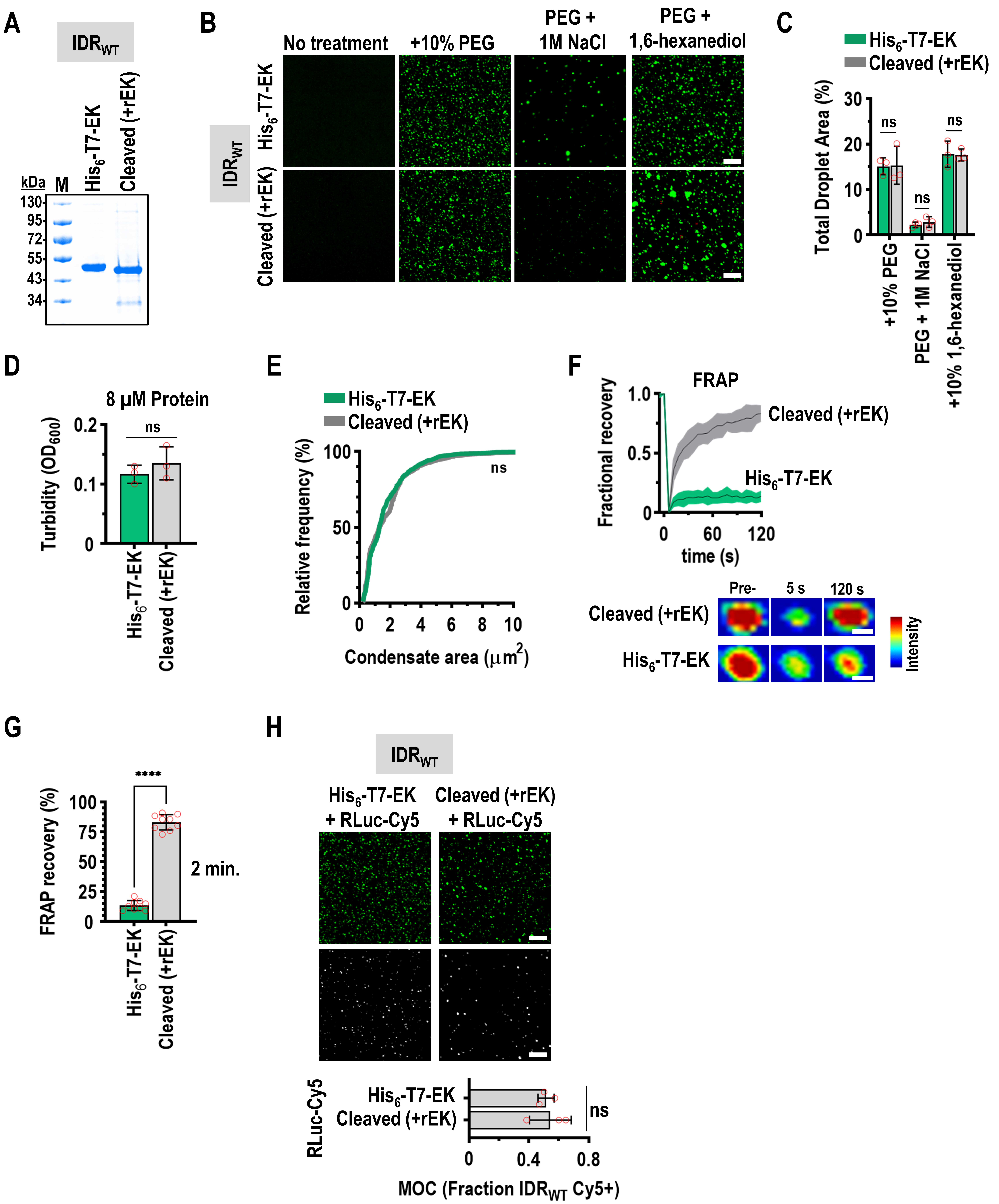

Supplement: S1 Fig — (A) Coomassie-stained SDS-PAGE gel shows expected subtle downward shift by IDRWT following His-tag cleavage with recombinant enterokinase (rEK). (B) Untagged or tagged IDRWT droplet formation was monitored under various conditions by confocal microscopy. Bar scale: 20 μm. (C) Total droplet areas (%) were measured from confocal images using ImageJ. Error bars denote standard deviations and data points represent individual 20x fields (3 total). ns: not significant by two-way ANOVA and Sidak’s multiple comparisons test. (D) In vitro turbidity assays (OD600) were performed with 8 μM tagged or untagged IDRWT. Three biological replicates are shown (red circles). ns: not significant by unpaired t test. (E) Particle sizes of tagged and untagged IDRWT droplets from three 20x fields were measured using ImageJ. ns: not significant by two-tailed Mann-Whitney rank test. (F) Droplet dynamics of His-tagged and untagged IDRWT were measured by FRAP. Results are from 9 FRAP experiments with shaded areas representing standard deviations for each condition. Representative droplets and heat map overlays are shown for each construct. (G) End-point (2 min.) FRAP recoveries were compared between tagged and untagged IDRWT. Error bars denote standard deviations and data points represent individual FRAP experiments (9 total) ****P<0.0001 unpaired t test. (H) RLuc-Cy5 RNAs were mixed with tagged and untagged IDRWT at a 1:500 RNA:protein ratio. The fraction of IDRWT signal that was positive for Cy5-labelled RNA was determined by Mander’s Overlap Coefficient (MOC) analysis. Error bars denote standard deviations and data points represent individual 20x fields. ns: not significant by unpaired t test. (TIF) [file ppat.1009622.s001.tif]

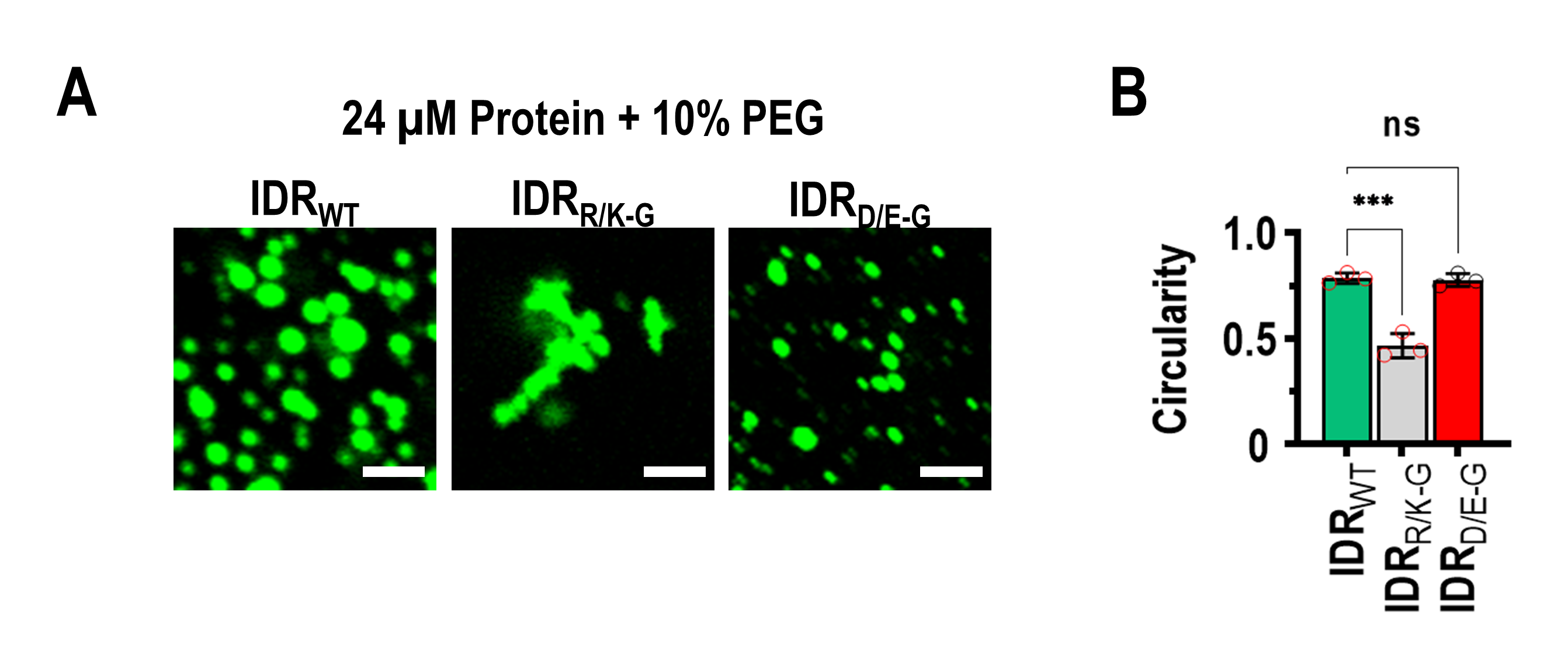

Supplement: S2 Fig — (A) 24 μM protein was mixed with 10% PEG-8000 to induce phase separation in standard assay buffer. Droplet or aggregate formation was visualized by confocal microscopy. Bar scale: 5 μm. (B) Individual droplets or aggregates were assessed for circularity using ImageJ. Data points represent individual 20x fields and error bars denote standard deviations. ***P<0.001, ns: not significant by one-way ANOVA with Dunnett’s multiple comparisons test. (TIF) [file ppat.1009622.s002.TIF]

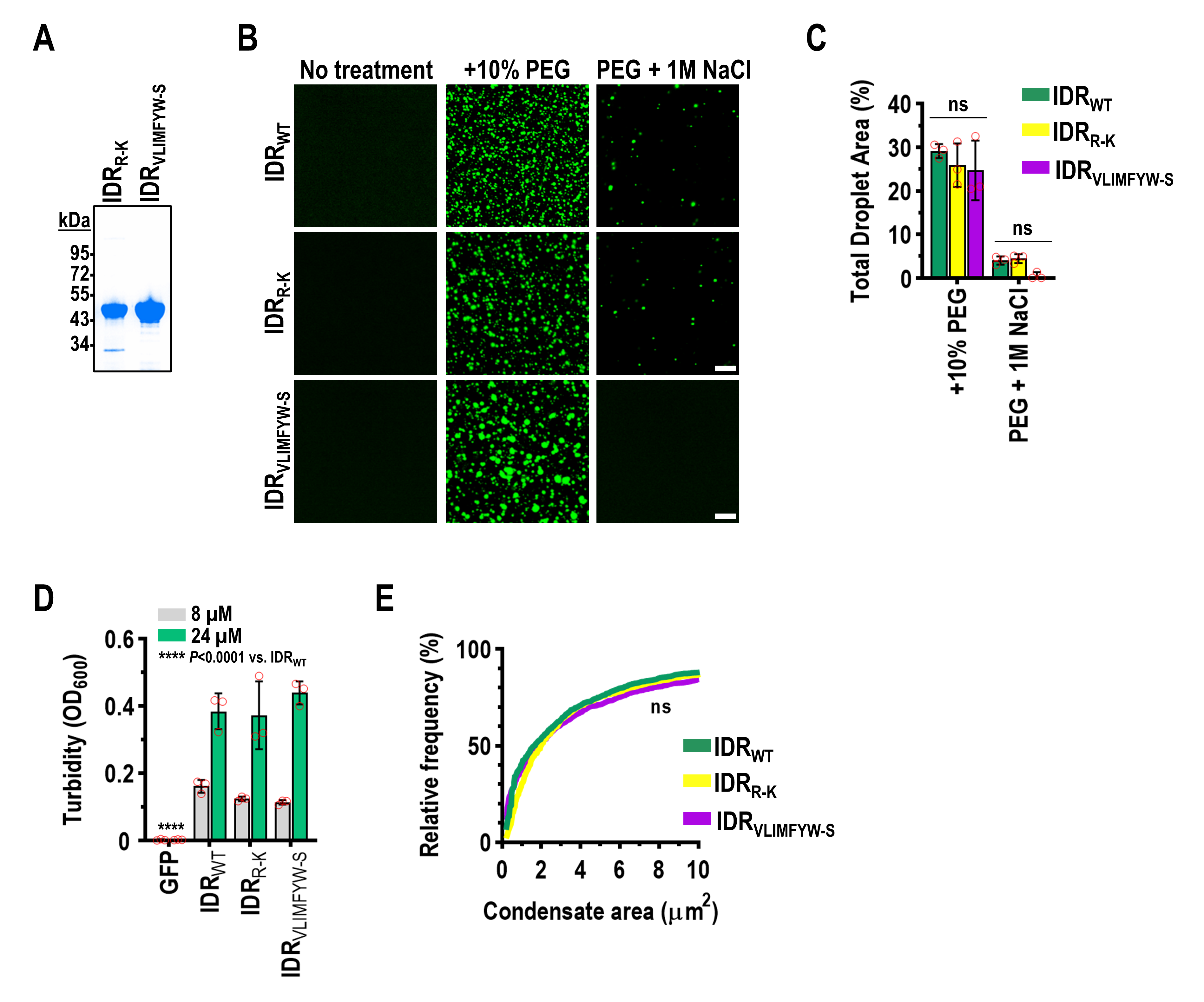

Supplement: S3 Fig — (A) SDS-PAGE analysis of Coomassie-stained recombinant IDRR-K and IDRVLIMFYW-S. Marker weights are shown on left in kilodaltons (kDa). IDRR-K contains lysine substitutions for all arginines whereas IDRVLIMFYW-S contains serine substitutions for all hydrophobic residues. (B) Droplet formation by IDRWT, IDRR-K, and IDRVLIMFYW-S was visualized by confocal microscopy under crowding conditions with and without 1 M NaCl. (C) Total droplet areas were measured from three separate 20x fields for each condition (red circles). Error bars denote standard deviations. ns: not significant by two-way ANOVA and Sidak’s multiple comparisons test. (D) Turbidity assays (OD600) comparing GFP, IDRWT, IDRR-K, and IDRVLIMFYW-S phase separation propensities. Error bars denote standard deviations and data points represent biological replicates (3 total). **** P<0.0001 by two-way ANOVA with Dunnett’s multiple comparisons test vs. IDRWT. (E) Mean condensate sizes for IDRR-K and IDRVLIMFYW-S mutants and wild-type IDRWT were plotted by cumulative distribution frequency. Particle sizes were measured from three representative 20x fields using ImageJ. ns: not significant, two-tailed Mann-Whitney tests compared to IDRWT. (TIF) [file ppat.1009622.s003.TIF]

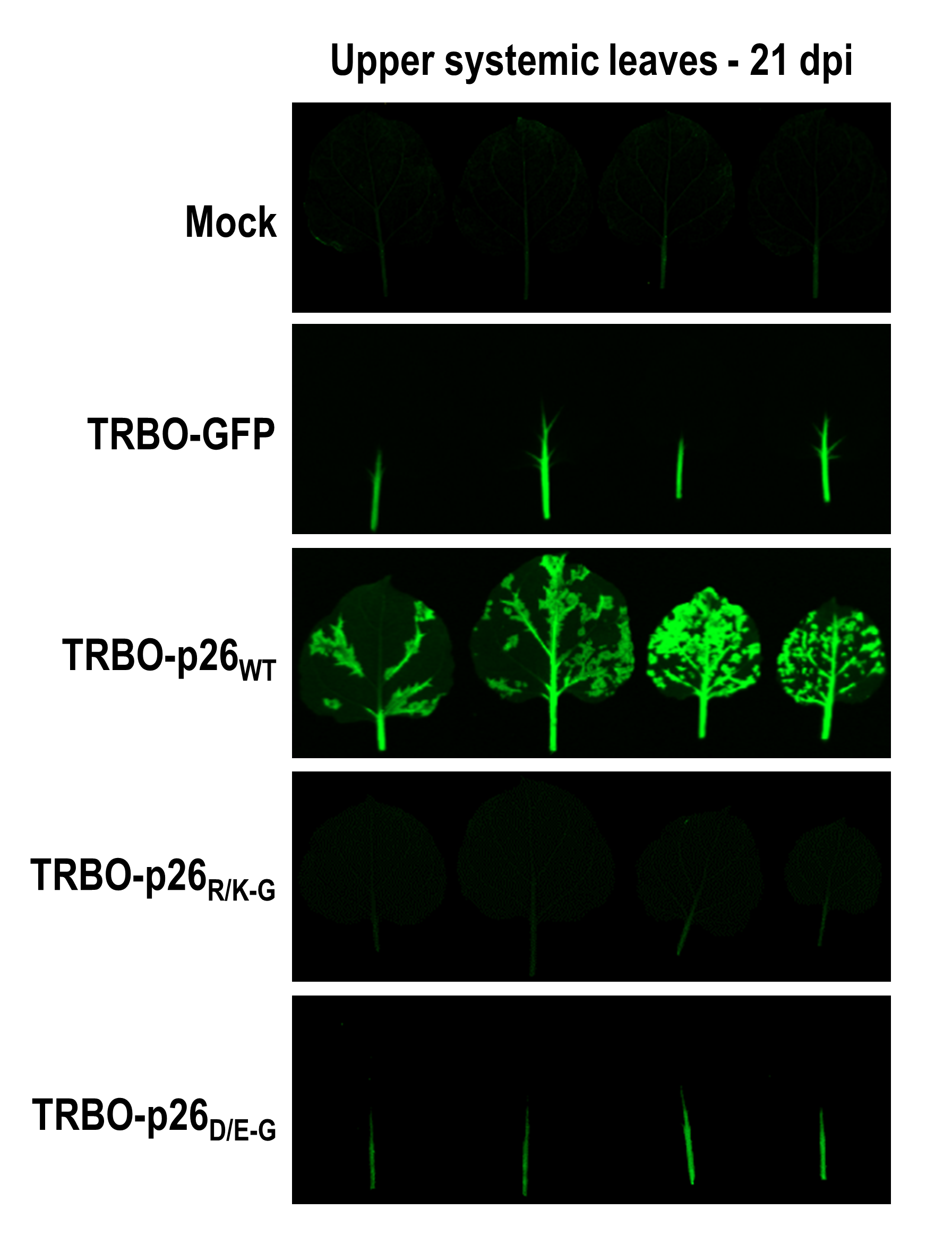

Supplement: S4 Fig — At 21 dpi, upper N. benthamiana systemic leaves were imaged at 488 nm. TRBO-GFP and TRBO-p26D/E-G were mostly restricted to the petiole and midrib of systemic leaves. In contrast, TRBO-p26WT invaded the lamina of systemic leaves. Images are representative of three independent experiments with at least four plants for each condition. (TIF) [file ppat.1009622.s004.TIF]
