# Supplementary material for: Phase separation of a plant virus movement protein and cellular factors support virus-host interactions
Source: PLoS Pathog. 2021 Sep 20;17(9):e1009622. doi: 10.1371/journal.ppat.1009622 (PMC8483311; doi:10.1371/journal.ppat.1009622)
Supplement: S1 Appendix — Details include recombinant protein amino acid sequences with predicted molecular weights, DNA coding sequences for all cloning inserts, and cloning strategies with restriction sites used. Full-length PEMV2 genome (not deposited in GenBank) is included. (DOCX) [file ppat.1009622.s005.docx]

**Wild-type and mutant IDRs. Synthetic gBlock fragments were cloned into pRSET-his-eGFP. IDR-GFP fusions were expressed in *E. coli* and purified for *in vitro* phase separation assays.**

**IDR or Cterm amino acid sequence**

**Universal gBlock tag for PCR amplification**

**Underlined sequence denotes restriction site used for ligation**

**GFP**

>Amino acid sequence of recombinant protein (including tags)

MRGSHHHHHHGMASMTGGQQMGRDLYDDDDKDRWGSMVSKGEELFTGVVPILVELDGDVNGHKFSVSGEGEGDATYGKLTLKFICTTGKLPVPWPTLVTTLTYGVQCFSRYPDHMKQHDFFKSAMPEGYVQERTIFFKDDGNYKTRAEVKFEGDTLVNRIELKGIDFKEDGNILGHKLEYNYNSHNVYIMADKQKNGIKVNFKIRHNIEDGSVQLADHYQQNTPIGDGPVLLPDNHYLSTQSKLSKDPNEKRDHMVLLEFVTAAGITLGMDELYK

MW = 31.1 kDa

**IDR_WT_ (amino acids 1-132)**

>Amino acid sequence of recombinant protein (including tags and GFP)

MRGSHHHHHHGMASMTGGQQMGRDLYDDDDKDRWGSMAVGKYMTIIINVNNDERKQPEGATGSSVRRGDNKRTRGNKPRSHHPGSRERKGYNHPSPTPKNSKQGQLRTEAVQEHPKHGGTAFRRESGGSVHPSHPRRRARRGGDMAPRQHPPPPRERRTKTETQAERRGSMVSKGEELFTGVVPILVELDGDVNGHKFSVSGEGEGDATYGKLTLKFICTTGKLPVPWPTLVTTLTYGVQCFSRYPDHMKQHDFFKSAMPEGYVQERTIFFKDDGNYKTRAEVKFEGDTLVNRIELKGIDFKEDGNILGHKLEYNYNSHNVYIMADKQKNGIKVNFKIRHNIEDGSVQLADHYQQNTPIGDGPVLLPDNHYLSTQSKLSKDPNEKRDHMVLLEFVTAAGITLGMDELYK

MW = 46.2 kDa

-PCR amplified using IDR_BamHI_F and IDR_BamHI_R primers. Digested with BamHI prior to ligation into pRSET-his-eGFP.

>PCR amplified sequence (full-length PEMV2 used as template)

GCAGCAGGATCCATGGCGGTAGGGAAATATATGACGATAATCATTAATGTCAATAACGACGAGCGCAAGCAACCAGAAGGAGCTACTGGCAGCTCTGTACGGCGAGGTGACAATAAAAGAACTCGAGGAAACAAACCTCGGAGTCATCACCCCGGTTCGCGCGAACGAAAAGGTTACAATCACCCCTCTCCTACCCCCAAAAACTCAAAGCAGGGTCAGCTCCGTACTGAAGCGGTTCAGGAGCACCCGAAACACGGGGGGACTGCTTTCCGTAGAGAAAGTGGTGGTAGTGTTCACCCCTCACATCCCCGACGACGTGCTAGGAGAGGTGGAGATATGGCTCCACGACAGCATCCTCCCCCACCTCGGGAGCGTCGGACCAAGACTGAAACTCAAGCTGAGCGAAGGGGATCCGCAGCA

**IDR_R/K-G_**

>Amino acid sequence

MRGSHHHHHHGMASMAVGGYMTIIINVNNDEGGQPEGATGSSVGGGDNGGTGGNGPGSHHPGSGEGGGYNHPSPTPGNSGQGQLGTEAVQEHPGHGGTAFGGESGGSVHPSHPGGGAGGGGDMAPGQHPPPPGEGGTGTETQAEGGGSMVSKGEELFTGVVPILVELDGDVNGHKFSVSGEGEGDATYGKLTLKFICTTGKLPVPWPTLVTTLTYGVQCFSRYPDHMKQHDFFKSAMPEGYVQERTIFFKDDGNYKTRAEVKFEGDTLVNRIELKGIDFKEDGNILGHKLEYNYNSHNVYIMADKQKNGIKVNFKIRHNIEDGSVQLADHYQQNTPIGDGPVLLPDNHYLSTQSKLSKDPNEKRDHMVLLEFVTAAGITLGMDELYK

MW = 40.8 kDa

-PCR amplified using gBlock_Tag_F and gBlock_Tag_R primers. Digested with NheI and BamHI prior to ligation into pRSET-his-eGFP.

>Synthetic DNA sequence

GGCATGCATTCCGCAACGAGGCAGCAGCTAGC ATG GCC GTG GGC GGG TAC ATG ACC ATC ATC ATC AAC GTC AAT AAC GAT GAG GGA GGA CAA CCG GAG GGT GCC ACG GGA TCT TCC GTC GGA GGA GGT GAC AAC GGA GGT ACA GGT GGA AAT GGT CCT GGT AGC CAT CAT CCC GGT TCT GGG GAG GGA GGG GGA TAC AAT CAT CCC AGT CCT ACA CCA GGT AAT TCG GGT CAG GGT CAG CTG GGT ACG GAG GCT GTC CAA GAA CAT CCC GGC CAC GGT GGC ACA GCT TTT GGA GGT GAG AGC GGC GGC TCT GTT CAC CCA TCT CAC CCC GGC GGC GGT GCC GGA GGT GGT GGA GAC ATG GCA CCG GGG CAA CAT CCA CCT CCT CCA GGC GAA GGA GGG ACT GGG ACA GAA ACT CAA GCG GAG GGC GGG GGATCCGCAGCACCATTATCCGGCGATGCGACG

**IDR_D/E-G_**

>Amino acid sequence

MRGSHHHHHHGMASMAVGKYMTIIINVNNGGRKQPGGATGSSVRRGGNKRTRGNKPRSHHPGSRGRKGYNHPSPTPKNSKQGQLRTGAVQGHPKHGGTAFRRGSGGSVHPSHPRRRARRGGGMAPRQHPPPPRGRRTKTGTQAGRRGSMVSKGEELFTGVVPILVELDGDVNGHKFSVSGEGEGDATYGKLTLKFICTTGKLPVPWPTLVTTLTYGVQCFSRYPDHMKQHDFFKSAMPEGYVQERTIFFKDDGNYKTRAEVKFEGDTLVNRIELKGIDFKEDGNILGHKLEYNYNSHNVYIMADKQKNGIKVNFKIRHNIEDGSVQLADHYQQNTPIGDGPVLLPDNHYLSTQSKLSKDPNEKRDHMVLLEFVTAAGITLGMDELYK

MW = 42.8 kDa

-PCR amplified using gBlock_Tag_F and gBlock_Tag_R primers. Digested with NheI and BamHI prior to ligation into pRSET-his-eGFP.

>Synthetic DNA sequence

GGCATGCATTCCGCAACGAGGCAGCAGCTAGC ATG GCA GTG GGT AAA TAT ATG ACC ATC ATC ATT AAC GTT AAT AAT GGG GGA CGC AAG CAG CCT GGC GGT GCG ACC GGG TCG TCG GTA CGC CGT GGT GGA AAT AAA CGT ACC CGC GGA AAC AAG CCT CGC TCA CAT CAC CCG GGA AGT CGT GGA CGT AAA GGT TAT AAC CAT CCC TCA CCA ACA CCG AAA AAT TCC AAA CAA GGT CAG CTT CGT ACC GGA GCC GTT CAG GGC CAC CCA AAA CAC GGA GGA ACA GCC TTT CGC CGC GGA AGC GGT GGC TCC GTA CAT CCA TCC CAT CCA CGC CGC CGT GCG CGT CGT GGT GGC GGA ATG GCG CCG CGT CAG CAT CCC CCT CCT CCA CGC GGG CGT CGT ACT AAA ACG GGG ACC CAA GCG GGA CGC CGT GGATCCGCAGCACCATTATCCGGCGATGCGACG

**IDR_ΔNLS_**

>Amino acid sequence

MRGSHHHHHHGMASMAVGKYMTIIINVNNDERKQPEGATGSSVRRGDNKRTRGNKPRSHHPGSRERKGYNHPSPTPKNSKQGQLRTEAVQEHPKHGGTAFRRESGGSVHPSHPGGDMAPRQHPPPPRERRTKTETQAERRGSMVSKGEELFTGVVPILVELDGDVNGHKFSVSGEGEGDATYGKLTLKFICTTGKLPVPWPTLVTTLTYGVQCFSRYPDHMKQHDFFKSAMPEGYVQERTIFFKDDGNYKTRAEVKFEGDTLVNRIELKGIDFKEDGNILGHKLEYNYNSHNVYIMADKQKNGIKVNFKIRHNIEDGSVQLADHYQQNTPIGDGPVLLPDNHYLSTQSKLSKDPNEKRDHMVLLEFVTAAGITLGMDELYK

MW = 42.8 kDa

-PCR amplified using gBlock_Tag_F and gBlock_Tag_R primers. Digested with NheI and BamHI prior to ligation into pRSET-his-eGFP.

>Synthetic DNA sequence

GGCATGCATTCCGCAACGAGGCAGCAGCTAGC ATG GCT GTC GGC AAG TAT ATG ACT ATC ATT ATC AAT GTT AAC AAT GAT GAA CGC AAG CAG CCA GAG GGG GCA ACA GGA AGT TCT GTG CGT CGT GGA GAC AAT AAG CGT ACA CGC GGC AAC AAG CCC CGC TCG CAC CAT CCT GGA AGT CGT GAG CGT AAA GGG TAT AAT CAC CCG TCG CCG ACA CCC AAG AAT TCT AAG CAG GGA CAG CTT CGC ACA GAG GCA GTT CAA GAA CAC CCC AAA CAT GGG GGC ACG GCT TTC CGC CGT GAA AGC GGG GGA AGC GTA CAT CCC TCA CAT CCA GGG GGC GAC ATG GCT CCT CGC CAA CAC CCT CCA CCA CCT CGC GAG CGT CGC ACT AAA ACA GAG ACT CAG GCG GAA CGT CGT GGATCCGCAGCACCATTATCCGGCGATGCGACG

**Cterm_-10aa**

>Amino acid sequence

MRGSHHHHHHGMASAQALSVLPTLLDCIGGLDLGPAEVLLHCHRAVRRQLRTGVQPIQPVPHVESTHRSSDPQLLESSTTCSANLQNDGAGRVIGGGIPTAIPEGSDVGSMVSKGEELFTGVVPILVELDGDVNGHKFSVSGEGEGDATYGKLTLKFICTTGKLPVPWPTLVTTLTYGVQCFSRYPDHMKQHDFFKSAMPEGYVQERTIFFKDDGNYKTRAEVKFEGDTLVNRIELKGIDFKEDGNILGHKLEYNYNSHNVYIMADKQKNGIKVNFKIRHNIEDGSVQLADHYQQNTPIGDGPVLLPDNHYLSTQSKLSKDPNEKRDHMVLLEFVTAAGITLGMDELYK

MW = 38.5 kDa

-PCR amplified using Cterm_NheI_F and Cterm_-10aa_BamHI_R primers. Digested with NheI and BamHI prior to ligation into pRSET-his6-eGFP.

>PCR amplified sequence (full-length PEMV2 used as template)

GCAGCAGCTAGCGCCCAAGCTCTTAGCGTTCTACCCACCCTACTCGATTGCATTGGGGGACTCGATCTCGGGCCAGCCGAGGTCCTTCTCCATTGTCACCGAGCTGTTCGAAGGCAACTTCGCACCGGGGTGCAGCCCATTCAGCCTGTTCCTCATGTGGAGTCCACGCATCGAAGCAGTGACCCACAACTACTTGAGTCGTCCACCACGTGCTCTGCCAATTTGCAGAACGATGGTGCGGGACGCGTTATCGGAGGTGGCATCCCAACAGCAATACCTGAAGGGAGCGATGTCGGATCCGCAGCA

**IDR_R-K_**

>Amino acid sequence of recombinant protein (including tags and GFP)

MRGSHHHHHHGMASMTGGQQMGRDLYDDDDKDRWGSMAVGKYMTIIINVNNDEKKQPEGATGSSVKKGDNKKTKGNKPKSHHPGSKEKKGYNHPSPTPKNSKQGQLKTEAVQEHPKHGGTAFKKESGGSVHPSHPKKKAKKGGDMAPKQHPPPPKEKKTKTETQAEKKGSMVSKGEELFTGVVPILVELDGDVNGHKFSVSGEGEGDATYGKLTLKFICTTGKLPVPWPTLVTTLTYGVQCFSRYPDHMKQHDFFKSAMPEGYVQERTIFFKDDGNYKTRAEVKFEGDTLVNRIELKGIDFKEDGNILGHKLEYNYNSHNVYIMADKQKNGIKVNFKIRHNIEDGSVQLADHYQQNTPIGDGPVLLPDNHYLSTQSKLSKDPNEKRDHMVLLEFVTAAGITLGMDELYK

MW = 45.6 kDa

-PCR amplified using gBlock_Tag_F and gBlock_Tag_R primers. Digested with BamHI prior to ligation into pRSET-his-eGFP.

>Synthetic DNA sequence

GGCATGCATTCCGCAACGAGGCAGCAGGATCC ATG GCC GTA GGA AAG TAC ATG ACT ATT ATC ATC AAC GTC AAT AAT GAT GAG AAG AAG CAG CCA GAG GGA GCC ACT GGC TCC TCA GTA AAG AAG GGA GAT AAT AAA AAG ACA AAG GGC AAC AAG CCC AAG TCT CAC CAT CCC GGC TCT AAA GAG AAA AAA GGC TAT AAC CAC CCA TCC CCA ACT CCG AAG AAT AGC AAA CAA GGG CAG CTG AAA ACG GAA GCG GTT CAA GAA CAC CCA AAG CAT GGA GGA ACA GCG TTC AAA AAA GAA TCC GGC GGC TCT GTC CAC CCC AGC CAC CCA AAA AAA AAA GCT AAG AAG GGT GGA GAT ATG GCA CCT AAG CAA CAT CCT CCG CCA CCG AAA GAA AAG AAG ACG AAA ACA GAA ACT CAA GCC GAG AAG AAA GGATCCGCAGCACCATTATCCGGCGATGCGACG

**IDR_VLIMFYW-S_**

>Amino acid sequence of recombinant protein (including tags and GFP)

MRGSHHHHHHGMASMTGGQQMGRDLYDDDDKDRWGSSASGKSSTSSSNSNNDERKQPEGATGSSSRRGDNKRTRGNKPRSHHPGSRERKGSNHPSPTPKNSKQGQSRTEASQEHPKHGGTASRRESGGSSHPSHPRRRARRGGDSAPRQHPPPPRERRTKTETQAERRGSMVSKGEELFTGVVPILVELDGDVNGHKFSVSGEGEGDATYGKLTLKFICTTGKLPVPWPTLVTTLTYGVQCFSRYPDHMKQHDFFKSAMPEGYVQERTIFFKDDGNYKTRAEVKFEGDTLVNRIELKGIDFKEDGNILGHKLEYNYNSHNVYIMADKQKNGIKVNFKIRHNIEDGSVQLADHYQQNTPIGDGPVLLPDNHYLSTQSKLSKDPNEKRDHMVLLEFVTAAGITLGMDELYK

MW = 45.7 kDa

-PCR amplified using gBlock_Tag_F and gBlock_Tag_R primers. Digested with BamHI prior to ligation into pRSET-his-eGFP.

>Synthetic DNA sequence

GGCATGCATTCCGCAACGAGGCAGCAGGATCC TCC GCC AGC GGC AAG AGC TCA ACT TCC TCG AGT AAC TCT AAC AAC GAT GAA CGT AAA CAA CCC GAA GGG GCT ACT GGT TCC TCC TCG CGT CGC GGC GAC AAT AAA CGC ACC CGT GGC AAT AAA CCT CGC TCG CAT CAT CCC GGG TCA CGC GAA CGT AAG GGT AGT AAT CAC CCG AGT CCA ACC CCT AAA AAT TCT AAA CAA GGC CAA TCG CGC ACG GAG GCC TCT CAG GAG CAC CCA AAA CAC GGC GGT ACA GCT AGT CGC CGC GAA TCA GGA GGG TCG AGT CAT CCA AGC CAC CCT CGT CGT CGT GCT CGC CGC GGA GGT GAT TCT GCG CCT CGT CAA CAT CCC CCG CCG CCT CGC GAA CGC CGT ACA AAA ACA GAA ACC CAG GCG GAA CGC CGC GGATCCGCAGCACCATTATCCGGCGATGCGACG

**Full-length fibrillarin (Fib2) or the Fib2 GAR domain were cloned. pRSET-his-mCherry. Recombinant proteins were expressed in *E. coli* and purified for *in vitro* phase separation assays.**

**Fib2 amino acid sequence**

**GAR Domain**

**Underlined sequence denotes restriction site used for ligation**

**Full-length Fib2** Accession: AT4G25630.1 (CDS)

>Amino acid sequence of recombinant protein (with tags and mCherry)

MRGSHHHHHHGMASMRPPLTGSGGGFSGGRGRGGYSGGRGDGGFSGGRGGGGRGGGRGFSDRGGRGRGRGPPRGGARGGRGPAGRGGMKGGSKVIVEPHRHAGVFIAKGKEDALVTKNLVPGEAVYNEKRISVQNEDGTKTEYRVWNPFRSKLAAAILGGVDNIWIKPGAKVLYLGAASGTTVSHVSDLVGPEGCVYAVEFSHRSGRDLVNMAKKRTNVIPIIEDARHPAKYRMLVGMVDVIFSDVAQPDQARILALNASYFLKSGGHFVISIKANCIDSTVPAEAVFQTEVKKLQQEQFKPAEQVTLEPFERDHACVVGGYRMPKKPKAATAAGSVSKGEEDNMAIIKEFMRFKVHMEGSVNGHEFEIEGEGEGRPYEGTQTAKLKVTKGGPLPFAWDILSPQFMYGSKAYVKHPADIPDYLKLSFPEGFKWERVMNFEDGGVVTVTQDSSLQDGEFIYKVKLRGTNFPSDGPVMQKKTMGWEASSERMYPEDGALKGEIKQRLKLKDGGHYDAEVKTTYKAKKPVQLPGAYNVNIKLDITSHNEDYTIVEQYERAEGRHSTGGMDELYK

MW = 62.0 kDa

-PCR amplified using At_Fib2_NheI_F and At_Fib2_BamHI_R primers. cDNAs from Arabidopsis seedling RNAs were used as template

>PCR amplified sequence

GCAGCAGCTAGCATGAGACCTCCTCTAACTGGAAGTGGTGGTGGGTTCAGTGGTGGAAGAGGGCGTGGTGGATACAGTGGTGGTAGAGGTGACGGTGGATTCAGTGGTGGTCGAGGTGGCGGTGGTAGAGGAGGAGGAAGAGGTTTCAGCGACCGTGGTGGTCGCGGCAGAGGAAGAGGACCACCACGTGGTGGTGCTCGCGGCGGCAGAGGACCAGCTGGCCGTGGAGGCATGAAAGGAGGAAGCAAAGTGATTGTGGAACCTCACAGACACGCAGGAGTGTTCATTGCAAAGGGTAAAGAAGATGCCCTTGTTACCAAGAATTTGGTTCCTGGTGAAGCTGTCTACAACGAGAAGAGAATCTCTGTTCAGAACGAAGATGGAACCAAGACTGAATACAGAGTTTGGAATCCTTTCCGTTCGAAGTTGGCTGCTGCTATTCTTGGTGGTGTTGATAACATCTGGATTAAACCTGGTGCTAAAGTTCTATACCTTGGTGCTGCTTCTGGAACCACAGTCTCTCATGTGTCTGATCTTGTTGGCCCTGAGGGGTGTGTGTACGCGGTTGAGTTTTCTCATAGAAGTGGTAGAGATTTGGTGAACATGGCAAAGAAGAGAACCAATGTTATTCCAATCATCGAGGATGCTAGACACCCAGCTAAATACAGAATGCTTGTAGGCATGGTTGATGTTATCTTCTCTGATGTTGCTCAGCCAGATCAGGCTAGGATCTTGGCTTTGAATGCATCATACTTCCTCAAGTCAGGAGGACACTTTGTGATCTCAATAAAGGCAAACTGTATCGACTCCACCGTTCCAGCAGAAGCTGTGTTCCAGACTGAAGTGAAGAAGCTTCAACAGGAGCAATTCAAACCAGCTGAGCAAGTGACGCTTGAGCCATTTGAGCGTGACCATGCATGTGTCGTTGGTGGCTACCGTATGCCTAAGAAGCCAAAGGCTGCTACTGCTGCTGGATCCGCAGCA

**Fib2 GAR Domain**

>Amino acid sequence of recombinant protein (with tags and mCherry)

MRGSHHHHHHGMASGSGGGFSGGRGRGGYSGGRGDGGFSGGRGGGGRGGGRGFSDRGGRGRGRGPPRGGARGGRGPAGRGGMKGGGSVSKGEEDNMAIIKEFMRFKVHMEGSVNGHEFEIEGEGEGRPYEGTQTAKLKVTKGGPLPFAWDILSPQFMYGSKAYVKHPADIPDYLKLSFPEGFKWERVMNFEDGGVVTVTQDSSLQDGEFIYKVKLRGTNFPSDGPVMQKKTMGWEASSERMYPEDGALKGEIKQRLKLKDGGHYDAEVKTTYKAKKPVQLPGAYNVNIKLDITSHNEDYTIVEQYERAEGRHSTGGMDELYK

MW = 34.7 kDa

-PCR amplified using At_Fib2GAR_NheI_F and At_Fib2GAR_BamHI_R primers. Full-length Fib2-mCherry construct above was used as template)

>PCR amplified sequence

GCAGCAGCTAGCGGAAGTGGTGGTGGGTTCAGTGGTGGAAGAGGGCGTGGTGGATACAGTGGTGGTAGAGGTGACGGTGGATTCAGTGGTGGTCGAGGTGGCGGTGGTAGAGGAGGAGGAAGAGGTTTCAGCGACCGTGGTGGTCGCGGCAGAGGAAGAGGACCACCACGTGGTGGTGCTCGCGGCGGCAGAGGACCAGCTGGCCGTGGAGGCATGAAAGGAGGAGGATCCGCAGCA

**mCherry**

>Amino acid sequence of recombinant protein (including tags)

MRGSHHHHHHGMASMTGGQQMGRDLYDDDDKDRWGSVSKGEEDNMAIIKEFMRFKVHMEGSVNGHEFEIEGEGEGRPYEGTQTAKLKVTKGGPLPFAWDILSPQFMYGSKAYVKHPADIPDYLKLSFPEGFKWERVMNFEDGGVVTVTQDSSLQDGEFIYKVKLRGTNFPSDGPVMQKKTMGWEASSERMYPEDGALKGEIKQRLKLKDGGHYDAEVKTTYKAKKPVQLPGAYNVNIKLDITSHNEDYTIVEQYERAEGRHSTGGMDELYK

MW = 30.7 kDa

**Full-length p26:GFP fusions were cloned into either pBIN61S or pJL-TRBO (TMV vector). Synthetic gBlock fragments were synthesized containing desired mutations.**

**Universal gBlock tag for PCR amplification**

**GFP coding sequence**

**p26_WT_**

-PCR amplified using p26_BamHI_F and SalI_GFP_R primers for digestion and ligation into pBIN61S

>PCR amplified sequence (used pJL-p26:GFP as template)

GCAGCAGGATCCATGGCGGTAGGGAAATATATGACGATAATCATTAATGTCAATAACGACGAGCGCAAGCAACCAGAAGGAGCTACTGGCAGCTCTGTACGGCGAGGTGACAATAAAAGAACTCGAGGAAACAAACCTCGGAGTCATCACCCCGGTTCGCGCGAACGAAAAGGTTACAATCACCCCTCTCCTACCCCCAAAAACTCAAAGCAGGGTCAGCTCCGTACTGAAGCGGTTCAGGAGCACCCGAAACACGGGGGGACTGCTTTCCGTAGAGAAAGTGGTGGTAGTGTTCACCCCTCACATCCCCGACGACGTGCTAGGAGAGGTGGAGATATGGCTCCACGACAGCATCCTCCCCCACCTCGGGAGCGTCGGACCAAGACTGAAACTCAAGCTGAGCGAAGGGCCCAAGCTCTTAGCGTTCTACCCACCCTACTCGATTGCATTGGGGGACTCGATCTCGGGCCAGCCGAGGTCCTTCTCCATTGTCACCGAGCTGTTCGAAGGCAACTTCGCACCGGGGTGCAGCCCATTCAGCCTGTTCCTCATGTGGAGTCCACGCATCGAAGCAGTGACCCACAACTACTTGAGTCGTCCACCACGTGCTCTGCCAATTTGCAGAACGATGGTGCGGGACGCGTTATCGGAGGTGGCATCCCAACAGCAATACCTGAAGGGAGCGATGTCGAACAGGTATGCCATGCCTCTCACTACGGGATGAGTAAAGGAGAAGAACTTTTCACTGGAGTTGTCCCAATTCTTGTTGAATTAGATGGTGATGTTAATGGGCACAAATTTTCTGTCAGTGGAGAGGGTGAAGGTGATGCAACATACGGAAAACTTACCCTTAAATTTATTTGCACTACTGGAAAACTACCTGTTCCATGGCCAACACTTGTCACTACTTTCTCTTATGGTGTTCAATGCTTTTCAAGATACCCAGATCATATGAAGCGGCACGACTTCTTCAAGAGCGCCATGCCTGAGGGATACGTGCAGGAGAGGACCATCTTCTTCAAGGACGACGGGAACTACAAGACACGTGCTGAAGTCAAGTTTGAGGGAGACACCCTCGTCAACAGGATCGAGCTTAAGGGAATCGATTTCAAGGAGGACGGAAACATCCTCGGCCACAAGTTGGAATACAACTACAACTCCCACAACGTATACATCATGGCAGACAAACAAAAGAATGGAATCAAAGTTAACTTCAAAATTAGACACAACATTGAAGATGGAAGCGTTCAACTAGCAGACCATTATCAACAAAATACTCCAATTGGCGATGGCCCTGTCCTTTTACCAGACAACCATTACCTGTCCACACAATCTGCCCTTTCGAAAGATCCCAACGAAAAGAGAGACCACATGGTCCTTCTTGAGTTTGTAACAGCTGCTGGGATTACACATGGCATGGATGAACTATACAAATAGGTCGACGCAGCA

>Amino acid sequence of expressed protein (including GFP)

MAVGKYMTIIINVNNDERKQPEGATGSSVRRGDNKRTRGNKPRSHHPGSRERKGYNHPSPTPKNSKQGQLRTEAVQEHPKHGGTAFRRESGGSVHPSHPRRRARRGGDMAPRQHPPPPRERRTKTETQAERRAQALSVLPTLLDCIGGLDLGPAEVLLHCHRAVRRQLRTGVQPIQPVPHVESTHRSSDPQLLESSTTCSANLQNDGAGRVIGGGIPTAIPEGSDVEQVCHASHYGMSKGEELFTGVVPILVELDGDVNGHKFSVSGEGEGDATYGKLTLKFICTTGKLPVPWPTLVTTFSYGVQCFSRYPDHMKRHDFFKSAMPEGYVQERTIFFKDDGNYKTRAEVKFEGDTLVNRIELKGIDFKEDGNILGHKLEYNYNSHNVYIMADKQKNGIKVNFKIRHNIEDGSVQLADHYQQNTPIGDGPVLLPDNHYLSTQSALSKDPNEKRDHMVLLEFVTAAGITHGMDELYK

MW = 52.7 kDa

**p26_R/K-G_ (For pBIN61S)**

-PCR amplified using gBlock_Tag_F and gBlock_Tag_R primers before digestion with BamHI and SalI for ligation into pBIN61S

>Synthetic DNA sequence

GGCATGCATTCCGCAACGAGGCAGCAGGATCCACA ATG GCC GTT GGC GGG TAC ATG ACC ATC ATC ATA AAT GTA AAT AAT GAT GAG GGC GGA CAA CCT GAG GGT GCG ACG GGA AGT TCA GTC GGT GGA GGT GAT AAT GGT GGT ACT GGG GGT AAC GGA CCA GGG TCC CAT CAC CCC GGT AGC GGT GAA GGG GGC GGA TAT AAC CAT CCA TCT CCA ACA CCG GGC AAT AGT GGG CAG GGG CAA CTG GGA ACA GAA GCC GTA CAG GAA CAC CCC GGA CAT GGA GGT ACT GCG TTT GGT GGA GAA AGT GGC GGC AGT GTT CAT CCG AGT CAC CCA GGG GGT GGT GCA GGC GGT GGA GGT GAT ATG GCA CCA GGC CAA CAT CCT CCA CCT CCC GGA GAA GGA GGT ACA GGG ACT GAA ACC CAA GCA GAA GGT GGG GCT CAA GCC CTG TCC GTC CTA CCG ACT TTG CTG GAC TGT ATA GGT GGG CTG GAT CTC GGG CCA GCT GAA GTA CTT CTA CAC TGT CAC GGC GCG GTA GGA GGC CAG TTG GGT ACT GGG GTC CAA CCA ATA CAG CCG GTG CCA CAT GTC GAA AGT ACA CAT GGG TCA TCT GAC CCC CAG CTT CTG GAA TCT TCA ACA ACG TGC TCA GCT AAT TTG CAG AAT GAC GGG GCT GGA GGG GTG ATA GGT GGT GGA ATA CCA ACA GCG ATT CCA GAG GGT AGT GAT GTG GAG CAG GTT TGC CAT GCC TCA CAT TAC GGA ATGAGTAAAGGAGAAGAACTTTTCACTGGAGTTGTCCCAATTCTTGTTGAATTAGATGGTGATGTTAATGGGCACAAATTTTCTGTCAGTGGAGAGGGTGAAGGTGATGCAACATACGGAAAACTTACCCTTAAATTTATTTGCACTACTGGAAAACTACCTGTTCCATGGCCAACACTTGTCACTACTTTCTCTTATGGTGTTCAATGCTTTTCAAGATACCCAGATCATATGAAGCGGCACGACTTCTTCAAGAGCGCCATGCCTGAGGGATACGTGCAGGAGAGGACCATCTTCTTCAAGGACGACGGGAACTACAAGACACGTGCTGAAGTCAAGTTTGAGGGAGACACCCTCGTCAACAGGATCGAGCTTAAGGGAATCGATTTCAAGGAGGACGGAAACATCCTCGGCCACAAGTTGGAATACAACTACAACTCCCACAACGTATACATCATGGCAGACAAACAAAAGAATGGAATCAAAGTTAACTTCAAAATTAGACACAACATTGAAGATGGAAGCGTTCAACTAGCAGACCATTATCAACAAAATACTCCAATTGGCGATGGCCCTGTCCTTTTACCAGACAACCATTACCTGTCCACACAATCTGCCCTTTCGAAAGATCCCAACGAAAAGAGAGACCACATGGTCCTTCTTGAGTTTGTAACAGCTGCTGGGATTACACATGGCATGGATGAACTATACAAATAGGTCGACGCAGCACCATTATCCGGCGATGCGACG

>Amino acid sequence of expressed protein (including GFP)

MAVGGYMTIIINVNNDEGGQPEGATGSSVGGGDNGGTGGNGPGSHHPGSGEGGGYNHPSPTPGNSGQGQLGTEAVQEHPGHGGTAFGGESGGSVHPSHPGGGAGGGGDMAPGQHPPPPGEGGTGTETQAEGGAQALSVLPTLLDCIGGLDLGPAEVLLHCHGAVGGQLGTGVQPIQPVPHVESTHGSSDPQLLESSTTCSANLQNDGAGGVIGGGIPTAIPEGSDVEQVCHASHYGMSKGEELFTGVVPILVELDGDVNGHKFSVSGEGEGDATYGKLTLKFICTTGKLPVPWPTLVTTFSYGVQCFSRYPDHMKRHDFFKSAMPEGYVQERTIFFKDDGNYKTRAEVKFEGDTLVNRIELKGIDFKEDGNILGHKLEYNYNSHNVYIMADKQKNGIKVNFKIRHNIEDGSVQLADHYQQNTPIGDGPVLLPDNHYLSTQSALSKDPNEKRDHMVLLEFVTAAGITHGMDELYK

MW = 49.3 kDa

**p26_R/K-G_ (For pJL-TRBO)**

-PCR amplified using gBlock_Tag_F and gBlock_Tag_R primers before digestion with PacI and NotI for ligation into pJL-TRBO

>Synthetic DNA sequence

GGCATGCATTCCGCAACGAGGCAGCAttaattaaACA ATG GCC GTT GGC GGG TAC ATG ACC ATC ATC ATA AAT GTA AAT AAT GAT GAG GGC GGA CAA CCT GAG GGT GCG ACG GGA AGT TCA GTC GGT GGA GGT GAT AAT GGT GGT ACT GGG GGT AAC GGA CCA GGG TCC CAT CAC CCC GGT AGC GGT GAA GGG GGC GGA TAT AAC CAT CCA TCT CCA ACA CCG GGC AAT AGT GGG CAG GGG CAA CTG GGA ACA GAA GCC GTA CAG GAA CAC CCC GGA CAT GGA GGT ACT GCG TTT GGT GGA GAA AGT GGC GGC AGT GTT CAT CCG AGT CAC CCA GGG GGT GGT GCA GGC GGT GGA GGT GAT ATG GCA CCA GGC CAA CAT CCT CCA CCT CCC GGA GAA GGA GGT ACA GGG ACT GAA ACC CAA GCA GAA GGT GGG GCT CAA GCC CTG TCC GTC CTA CCG ACT TTG CTG GAC TGT ATA GGT GGG CTG GAT CTC GGG CCA GCT GAA GTA CTT CTA CAC TGT CAC GGC GCG GTA GGA GGC CAG TTG GGT ACT GGG GTC CAA CCA ATA CAG CCG GTG CCA CAT GTC GAA AGT ACA CAT GGG TCA TCT GAC CCC CAG CTT CTG GAA TCT TCA ACA ACG TGC TCA GCT AAT TTG CAG AAT GAC GGG GCT GGA GGG GTG ATA GGT GGT GGG ATC CCA ACA GCG ATT CCA GAG GGT AGT GAT GTG GAG CAG GTT TGC CAT GCC TCA CAT TAC GGA ATGAGTAAAGGAGAAGAACTTTTCACTGGAGTTGTCCCAATTCTTGTTGAATTAGATGGTGATGTTAATGGGCACAAATTTTCTGTCAGTGGAGAGGGTGAAGGTGATGCAACATACGGAAAACTTACCCTTAAATTTATTTGCACTACTGGAAAACTACCTGTTCCATGGCCAACACTTGTCACTACTTTCTCTTATGGTGTTCAATGCTTTTCAAGATACCCAGATCATATGAAGCGGCACGACTTCTTCAAGAGCGCCATGCCTGAGGGATACGTGCAGGAGAGGACCATCTTCTTCAAGGACGACGGGAACTACAAGACACGTGCTGAAGTCAAGTTTGAGGGAGACACCCTCGTCAACAGGATCGAGCTTAAGGGAATCGATTTCAAGGAGGACGGAAACATCCTCGGCCACAAGTTGGAATACAACTACAACTCCCACAACGTATACATCATGGCAGACAAACAAAAGAATGGAATCAAAGTTAACTTCAAAATTAGACACAACATTGAAGATGGAAGCGTTCAACTAGCAGACCATTATCAACAAAATACTCCAATTGGCGATGGCCCTGTCCTTTTACCAGACAACCATTACCTGTCCACACAATCTGCCCTTTCGAAAGATCCCAACGAAAAGAGAGACCACATGGTCCTTCTTGAGTTTGTAACAGCTGCTGGGATTACACATGGCATGGATGAACTATACAAATAGgcggccgcGCAGCACCATTATCCGGCGATGCGACG

>Amino acid sequence of expressed protein (including GFP)

MAVGGYMTIIINVNNDEGGQPEGATGSSVGGGDNGGTGGNGPGSHHPGSGEGGGYNHPSPTPGNSGQGQLGTEAVQEHPGHGGTAFGGESGGSVHPSHPGGGAGGGGDMAPGQHPPPPGEGGTGTETQAEGGAQALSVLPTLLDCIGGLDLGPAEVLLHCHGAVGGQLGTGVQPIQPVPHVESTHGSSDPQLLESSTTCSANLQNDGAGGVIGGGIPTAIPEGSDVEQVCHASHYGMSKGEELFTGVVPILVELDGDVNGHKFSVSGEGEGDATYGKLTLKFICTTGKLPVPWPTLVTTFSYGVQCFSRYPDHMKRHDFFKSAMPEGYVQERTIFFKDDGNYKTRAEVKFEGDTLVNRIELKGIDFKEDGNILGHKLEYNYNSHNVYIMADKQKNGIKVNFKIRHNIEDGSVQLADHYQQNTPIGDGPVLLPDNHYLSTQSALSKDPNEKRDHMVLLEFVTAAGITHGMDELYK

MW = 49.3 kDa

**p26_D/E-G_**

-PCR amplified using gBlock_Tag_F and gBlock_Tag_R primers before digestion with PacI and NotI for ligation into pJL-TRBO

**-**PCR amplified using D/E-G_p26_BamHI_F and SalI_GFP_R primers before digestion and ligation into pBIN61S

>Synthetic DNA sequence

GGCATGCATTCCGCAACGAGGCAGCAttaattaaACA ATG GCT GTC GGC AAG TAT ATG ACT ATA ATC ATC AAC GTA AAT AAC GGT GGA CGA AAA CAG CCT GGC GGG GCG ACA GGG TCC TCA GTT AGA AGA GGA GGC AAC AAG CGA ACG CGA GGA AAC AAG CCC CGA TCC CAC CAC CCC GGA TCA AGG GGG CGT AAG GGA TAC AAC CAC CCG AGT CCC ACC CCG AAA AAC TCA AAG CAG GGT CAG CTT CGT ACA GGG GCT GTC CAA GGA CAT CCT AAG CAC GGG GGA ACG GCA TTC AGA CGA GGC TCC GGT GGA AGT GTG CAT CCG AGC CAT CCA AGG AGG CGT GCA AGA CGA GGT GGC GGA ATG GCA CCG CGT CAA CAT CCT CCT CCG CCT AGA GGG AGA CGA ACA AAG ACG GGT ACG CAA GCG GGA AGG CGT GCC CAG GCT CTT TCT GTT CTG CCC ACC CTT CTC GGG TGC ATA GGG GGT CTC GGC CTT GGC CCA GCC GGA GTG CTT CTC CAT TGC CAC CGT GCT GTG AGA AGA CAG CTA CGA ACG GGA GTC CAA CCT ATA CAA CCA GTA CCT CAT GTC GGT AGC ACT CAT CGT TCA TCT GGT CCG CAA TTA CTA GGT TCA TCT ACG ACT TGT AGC GCG AAC TTA CAA AAC GGT GGG GCT GGC AGA GTT ATT GGT GGA GGC ATT CCC ACT GCT ATC CCC GGC GGT TCA GGC GTG GGC CAG GTA TGC CAT GCT AGT CAT TAT GGA ATGAGTAAAGGAGAAGAACTTTTCACTGGAGTTGTCCCAATTCTTGTTGAATTAGATGGTGATGTTAATGGGCACAAATTTTCTGTCAGTGGAGAGGGTGAAGGTGATGCAACATACGGAAAACTTACCCTTAAATTTATTTGCACTACTGGAAAACTACCTGTTCCATGGCCAACACTTGTCACTACTTTCTCTTATGGTGTTCAATGCTTTTCAAGATACCCAGATCATATGAAGCGGCACGACTTCTTCAAGAGCGCCATGCCTGAGGGATACGTGCAGGAGAGGACCATCTTCTTCAAGGACGACGGGAACTACAAGACACGTGCTGAAGTCAAGTTTGAGGGAGACACCCTCGTCAACAGGATCGAGCTTAAGGGAATCGATTTCAAGGAGGACGGAAACATCCTCGGCCACAAGTTGGAATACAACTACAACTCCCACAACGTATACATCATGGCAGACAAACAAAAGAATGGAATCAAAGTTAACTTCAAAATTAGACACAACATTGAAGATGGAAGCGTTCAACTAGCAGACCATTATCAACAAAATACTCCAATTGGCGATGGCCCTGTCCTTTTACCAGACAACCATTACCTGTCCACACAATCTGCCCTTTCGAAAGATCCCAACGAAAAGAGAGACCACATGGTCCTTCTTGAGTTTGTAACAGCTGCTGGGATTACACATGGCATGGATGAACTATACAAATAGgcggccgcGCAGCACCATTATCCGGCGATGCGACG

>Amino acid sequence of expressed protein (including GFP)

MAVGKYMTIIINVNNGGRKQPGGATGSSVRRGGNKRTRGNKPRSHHPGSRGRKGYNHPSPTPKNSKQGQLRTGAVQGHPKHGGTAFRRGSGGSVHPSHPRRRARRGGGMAPRQHPPPPRGRRTKTGTQAGRRAQALSVLPTLLGCIGGLGLGPAGVLLHCHRAVRRQLRTGVQPIQPVPHVGSTHRSSGPQLLGSSTTCSANLQNGGAGRVIGGGIPTAIPGGSGVGQVCHASHYGMSKGEELFTGVVPILVELDGDVNGHKFSVSGEGEGDATYGKLTLKFICTTGKLPVPWPTLVTTFSYGVQCFSRYPDHMKRHDFFKSAMPEGYVQERTIFFKDDGNYKTRAEVKFEGDTLVNRIELKGIDFKEDGNILGHKLEYNYNSHNVYIMADKQKNGIKVNFKIRHNIEDGSVQLADHYQQNTPIGDGPVLLPDNHYLSTQSALSKDPNEKRDHMVLLEFVTAAGITHGMDELYK

MW = 51.2 kDa

**p26_ΔNLS_**

-PCR amplified using gBlock_Tag_F and gBlock_Tag_R primers before digestion with BamHI and SalI for ligation into pBIN61S

>Synthetic DNA sequence

GGCATGCATTCCGCAACGAGGCAGCAGGATCCACAATGGCGGTAGGGAAATATATGACGATAATCATTAATGTCAATAACGACGAGCGCAAGCAACCAGAAGGAGCTACTGGCAGCTCTGTACGGCGAGGTGACAATAAAAGAACTCGAGGAAACAAACCTCGGAGTCATCACCCCGGTTCGCGCGAACGAAAAGGTTACAATCACCCCTCTCCTACCCCCAAAAACTCAAAGCAGGGTCAGCTCCGTACTGAAGCGGTTCAGGAGCACCCGAAACACGGGGGGACTGCTTTCCGTAGAGAAAGTGGTGGTAGTGTTCACCCCTCACATCCCGGTGGAGATATGGCTCCACGACAGCATCCTCCCCCACCTCGGGAGCGTCGGACCAAGACTGAAACTCAAGCTGAGCGAAGGGCCCAAGCTCTTAGCGTTCTACCCACCCTACTCGATTGCATTGGGGGACTCGATCTCGGGCCAGCCGAGGTCCTTCTCCATTGTCACCGAGCTGTTCGAAGGCAACTTCGCACCGGGGTGCAGCCCATTCAGCCTGTTCCTCATGTGGAGTCCACGCATCGAAGCAGTGACCCACAACTACTTGAGTCGTCCACCACGTGCTCTGCCAATTTGCAGAACGATGGTGCGGGACGCGTTATCGGAGGTGGCATCCCAACAGCAATACCTGAAGGGAGCGATGTCGAACAGGTATGCCATGCCTCTCACTACGGGATGAGTAAAGGAGAAGAACTTTTCACTGGAGTTGTCCCAATTCTTGTTGAATTAGATGGTGATGTTAATGGGCACAAATTTTCTGTCAGTGGAGAGGGTGAAGGTGATGCAACATACGGAAAACTTACCCTTAAATTTATTTGCACTACTGGAAAACTACCTGTTCCATGGCCAACACTTGTCACTACTTTCTCTTATGGTGTTCAATGCTTTTCAAGATACCCAGATCATATGAAGCGGCACGACTTCTTCAAGAGCGCCATGCCTGAGGGATACGTGCAGGAGAGGACCATCTTCTTCAAGGACGACGGGAACTACAAGACACGTGCTGAAGTCAAGTTTGAGGGAGACACCCTCGTCAACAGGATCGAGCTTAAGGGAATCGATTTCAAGGAGGACGGAAACATCCTCGGCCACAAGTTGGAATACAACTACAACTCCCACAACGTATACATCATGGCAGACAAACAAAAGAATGGAATCAAAGTTAACTTCAAAATTAGACACAACATTGAAGATGGAAGCGTTCAACTAGCAGACCATTATCAACAAAATACTCCAATTGGCGATGGCCCTGTCCTTTTACCAGACAACCATTACCTGTCCACACAATCTGCCCTTTCGAAAGATCCCAACGAAAAGAGAGACCACATGGTCCTTCTTGAGTTTGTAACAGCTGCTGGGATTACACATGGCATGGATGAACTATACAAATAGGTCGACGCAGCACCATTATCCGGCGATGCGACG

>Amino acid sequence of expressed protein (including GFP)

MAVGKYMTIIINVNNDERKQPEGATGSSVRRGDNKRTRGNKPRSHHPGSRERKGYNHPSPTPKNSKQGQLRTEAVQEHPKHGGTAFRRESGGSVHPSHPGGDMAPRQHPPPPRERRTKTETQAERRAQALSVLPTLLDCIGGLDLGPAEVLLHCHRAVRRQLRTGVQPIQPVPHVESTHRSSDPQLLESSTTCSANLQNDGAGRVIGGGIPTAIPEGSDVEQVCHASHYGMSKGEELFTGVVPILVELDGDVNGHKFSVSGEGEGDATYGKLTLKFICTTGKLPVPWPTLVTTFSYGVQCFSRYPDHMKRHDFFKSAMPEGYVQERTIFFKDDGNYKTRAEVKFEGDTLVNRIELKGIDFKEDGNILGHKLEYNYNSHNVYIMADKQKNGIKVNFKIRHNIEDGSVQLADHYQQNTPIGDGPVLLPDNHYLSTQSALSKDPNEKRDHMVLLEFVTAAGITHGMDELYK

MW = 51.8 kDa

**Free GFP**

-PCR amplified using PacI_GFP_F and NotI_GFP_R primers before digestion and ligation into pJL-TRBO

>PCR amplified sequence (used pBIN-GFP as a template)

GCAGCAttaattaaACAATGAGTAAAGGAGAAGAACTTTTCACTGGAGTTGTCCCAATTCTTGTTGAATTAGATGGTGATGTTAATGGGCACAAATTTTCTGTCAGTGGAGAGGGTGAAGGTGATGCAACATACGGAAAACTTACCCTTAAATTTATTTGCACTACTGGAAAACTACCTGTTCCATGGCCAACACTTGTCACTACTTTCTCTTATGGTGTTCAATGCTTTTCAAGATACCCAGATCATATGAAGCGGCACGACTTCTTCAAGAGCGCCATGCCTGAGGGATACGTGCAGGAGAGGACCATCTTCTTCAAGGACGACGGGAACTACAAGACACGTGCTGAAGTCAAGTTTGAGGGAGACACCCTCGTCAACAGGATCGAGCTTAAGGGAATCGATTTCAAGGAGGACGGAAACATCCTCGGCCACAAGTTGGAATACAACTACAACTCCCACAACGTATACATCATGGCAGACAAACAAAAGAATGGAATCAAAGTTAACTTCAAAATTAGACACAACATTGAAGATGGAAGCGTTCAACTAGCAGACCATTATCAACAAAATACTCCAATTGGCGATGGCCCTGTCCTTTTACCAGACAACCATTACCTGTCCACACAATCTGCCCTTTCGAAAGATCCCAACGAAAAGAGAGACCACATGGTCCTTCTTGAGTTTGTAACAGCTGCTGGGATTACACATGGCATGGATGAACTATACAAATAGgcggccgcGCAGCA

>Amino acid sequence of expressed protein

MSKGEELFTGVVPILVELDGDVNGHKFSVSGEGEGDATYGKLTLKFICTTGKLPVPWPTLVTTFSYGVQCFSRYPDHMKRHDFFKSAMPEGYVQERTIFFKDDGNYKTRAEVKFEGDTLVNRIELKGIDFKEDGNILGHKLEYNYNSHNVYIMADKQKNGIKVNFKIRHNIEDGSVQLADHYQQNTPIGDGPVLLPDNHYLSTQSALSKDPNEKRDHMVLLEFVTAAGITHGMDELYK

MW = 26.9 kDa

**ΔNTF2-G3BP fused to RFP was cloned into pBIN61S. Full-length G3BP:RFP served as a template. See TAIR accession AT5G43960.1 for full-length G3BP sequence.**

NTF2 Domain

>Amino acid sequence – Full length G3BP

MATPYPGATQVGSYFVGQYYQVLQQQPDLIHQFYSEPSRAIRIDGDSTETANSLLHIHNMVMSLNFTAIEVKTINSVESWEGGVLVVVSGSVKTKEFSNRRSFVQTFFLAPQEKGYFVLSDVFLFVDEGTVYYHQPSYLSEIKHEAQLNPPTRHPDPQVSDYVLEEEASDYVNAVQIKDDLVDKYSLQEDQHQPQHEDYEDEVAIEETPREEVAVDVVHEHRAAPVEEPVGEKSKMSYASILKVAKEAATVPVAATQPSYNKSSQDINEWDQPMRTPSPQLAAPLAPIQQSNSSTYVSDYGAEAEDGSGFEDFEFKSVYVRNLPSDISASEIEEEFKNFGTIKPDGVFLRTRKDVMGVCYAFVEFEDMTSVENAIKASPIYLGGRQVYIEERRPNPAGVRGARRGGGRGRGGYPTEAPRGRFGGRGSGRGNQDGGDYRPRGNGYYRGGGR

-G3BP:RFP minus the NTF2 domain (amino acids 2-125) was PCR amplified using primers ΔNTF2_BamHI_F and RFP_SalI_R. PCR products were digested and ligated into pBIN61S

>PCR amplified sequence

GCAGCAGGATCCATGGTTGATGAGGGTACTGTTTACTATCATCAGCCTTCTTATTTATCTGAAATCAAGCATGAGGCTCAGCTTAATCCTCCTACCCGTCATCCAGATCCCCAAGTTTCTGACTATGTGCTGGAGGAAGAGGCAAGCGATTATGTGAACGCAGTCCAAATCAAAGATGATCTTGTTGATAAGTACAGTCTGCAAGAGGACCAACATCAACCTCAGCATGAAGACTATGAAGATGAAGTTGCGATTGAGGAAACACCTAGGGAAGAAGTTGCAGTTGATGTGGTACATGAACATCGGGCTGCACCAGTAGAGGAACCAGTTGGTGAAAAATCAAAGATGAGTTATGCTTCCATTTTAAAAGTTGCAAAGGAAGCAGCAACTGTACCTGTAGCTGCTACACAACCATCATATAACAAGAGCTCTCAAGATATTAATGAGTGGGATCAACCAATGCGGACTCCTTCCCCTCAGCTGGCTGCGCCTCTTGCCCCTATTCAGCAATCAAACTCATCCACATATGTTTCCGACTATGGAGCAGAGGCAGAAGATGGCTCCGGTTTTGAAGACTTTGAGTTCAAATCAGTGTACGTTAGGAATTTGCCTTCCGACATATCTGCTTCTGAAATAGAGGAAGAGTTTAAGAACTTCGGTACAATCAAGCCTGATGGTGTGTTCCTCAGAACCCGCAAGGATGTTATGGGCGTATGTTATGCATTTGTTGAGTTTGAGGACATGACTTCCGTTGAGAATGCGATTAAGGCTTCTCCTATATACTTGGGTGGAAGGCAAGTATACATTGAGGAACGAAGACCAAATCCCGCTGGTGTTCGCGGAGCAAGAAGAGGAGGAGGACGTGGAAGGGGTGGTTACCCAACAGAAGCACCAAGAGGCCGGTTTGGTGGGCGTGGGTCTGGCAGAGGAAACCAGGATGGAGGTGACTACAGGCCAAGAGGAAATGGTTACTACCGCGGTGGTGGTCGC ---- RFP_CDS ----TAAGTCGACGCAGCA

**PEMV2 genomic sequence. Full-length infectious clone was previously constructed by cloning PEMV2 genome into pCB301.**

**-source material for p26 IDR and C-term sequences**

>Full-length PEMV2 genomic sequence

GGGTATTTATAGAGATCAGTATGAACTGTGTCGCTAGGATCAAGCGGTGGTTCACACCTGACTTCACCCCTGGCGAGGGCGTGAAGTCTAGAGCTCAACTGGAAAGAGAGCTGGATCCCACCTGGGCGCTTCTCGTGTGCCAAGAACGAGCGCGTCGTGATGCTGACAGTATTGCTAATGAGTGGTACGAGGGCAGCATGGAGTGCAACCTCCTTATCCCTCGGCCCACAACCGAGGATGTATTTGGCCCCTCCATCGCCCCTGAGCCTGTGGCTCTAGTGGAGGAAACTACCCGTTCCCGCGCGCCGTGCGTGGATGTCCCTGCCGAGGAGTCCTGTAAGTCAGCGGAGATTGATCCTGTTGATCTCGCCAAGTTCGACTCCCTCCATCGTCGCCTGTTGGCTGAAGCCAACCCTTGCAGGGAAATGGTTCTGTGGGTGCCTCCTGGCCTACCAGCAGAGCGCGACGTCCTGCCCAGGGCACGTGGGGTGATAATGATCCCCGAAGTCCCTGCCTCTGCACATACCTTGTCCGTGAAGGTTATGGAGGCTGTGCGGTTGGCACAGGAAGTCTTGGCATCCCTTGCCAAGAGGGCCTTAGAGAAAAGGTCTACACCAACCCTTACCGCCCAGGCCCAGCCAGAGGCTACCCTGTCGGGGTGCGACTACCCGTATCAGGAGACTGGAGCAGCAGCCGCGTGGATAACGCCTGGCTGCATTGCCATGGAGCTCAGAGCCAAATTTGGCGTCTGCAAACGCACCCCCGCAAACTTAGAGATGGGGAGTCGCGTCGCCCGCGAGCTCCTGCGGGATAACTGTGTCACTTGCAGGGAGACCACGTGGTACACCAGTGCCATTGCTGTGGACCTGTGGTTGACCCCGACCGTCGTCGACCTGGCCTGTGGCCGGCGAGCGGCGGATTTTTGGTAGGGGCTGTGCTGCCTCGGCTGGGGGAAGACACCAGTGTGCGGTTTGACAACCTGCACCCCAGCATCGAGGTAATCAAGGCGGCTAGGCCCCGCCCAACCCAGAGGATGTCGTACCAAATCGACGTTGTGCGTCCTCTTGGAGATTTTGGTGTGCACAACAACTCCCTTGTTAACCTAGCCAGGGGAATTAATGAAAGGGTGTTCTACACGGACAATGCTAGGACAGAACCCCTTAAGCCTAAGGTTCCCTTCCCCTCATCACGGGAGCTGAAAACCTTCAGAGTCACCCCTTGGACCATGGATAGGGTTGTGGAGAGCTACACTGGGTCCCAGCGCACTCGCTATGCTAATGCGCGGGACAGCATATTATCCAACCCACTGAGTCCTAAAGATGCGCGGGTCAAGATGTTTGTCAAAGCTGAAAAGATAAATTTCACAGCCAAACCTGACCCCGCCCCTCGTGTGATACAGCCTAGGGATCCACGATCCAACATTGTCCTGGCTAAATACATCAAGCCTTTGGAGCCAATGTTGTACAAAGCACTGGGGAAACTTTACAAGTACCCCGCAGTTGCTAAGGGGTTTAACGCGGTTGAGACGGGGGAGATCATCGCCGGCAAGTGGCGGTGCTTCAAAGATCCTGTCGTCGTGGGATTAGACGCTTCCCGATTTGATCAGCATGTATCTGTCGAGGCGTTGCAGTTCACCCACGCGGTGTACAGAGGGTTCATCAAGTCACGGGAGTTTAACAACCTCCTACAGATGATGTACACCAACCGTGGCCTAGGGTCCGCTAAGGACGGATTCGTCCGTTACAAGGTTAAAGGTAGACGCATGAGCGGTGACATGGACACCTCCTTGGGCAACTGTGTGCTCATGGTGTTGCTCACCAGGAACCTTTGCAAGGTTCTAGGCATCCCGCACGAGCTCTTCAACAATGGTGATGATTGCATCGTCTTTTTCGATCGTTGCCACTTGGAGAAGTTCAACAATGCTGTCAAGACTTATTTTGCGGACCTAGGGTTTAAGATGAAGGTGGAACCGCCGGTTGACGTGTTGGAGAAAATAGAGTTCTGCCAAACGCAGCCTATCTATGACGGGGAGAAGTGGCGCACCGTGCGTTGCATCTCGAGTATCGGAAAAGATTGCTCATCCGTTATTAGTTGGGACCAATTGGAGGGGTGGTGGAATGCCATCGCCCAGAGTGGTCTGGCTGTGTGTGGCGGAATGCCGATATACACGTCGTTCTACCGGTGGCTAGCACGGGCCGGTAAGAGTGGGACCAAGTGTCAGTCACACCCCTTGTGGAAAAACGAGGGGTTGAATTGGTACAGGATGGGGATGGACCTTTCTCATGAGGTTAATGTTACCCCTCAGGCGCGCCTGTCTTTCTTCGCGGGTTTTGGTATTTCCCCCCCGATGCAGGTCGCCATTGAGGCGCTGTATGACAAGCTGCCTCCACCGTCCCCCCACCATGGTCCTCCGGTTAAGGCTGTAACACAGCGAGTGTTCACCAATTATTTCACGCCGGAAAGCGCCTGTGTTAGCATGAGCACGAATGAAGACAACAAATCTGACTTTGCTGTTTACGGCCCTGTGCCTACAGTGATGTCTCTTTGTGCTCAGTGTTAGGCTCTTAAATTTTAGCGATGGCGTGACACGGTTACACCCTGAATTGACAGGGTACAGATCAAGGGAAGCCGGGGAGTCACCAACCCACCCTGAATCGACAGGGCAAAAAGGGAAGCCGGGCACCGCCCACGTGGAATCGACCACGTCACCTTTTCGCGTCGACTATGCCGTCAACACCCTTTCGGCCCGCCAGCCTAGGACAATGGCGGTAGGGAAATATATGACGATAATCATTAATGTCAATAACGACGAGCGCAAGCAACCAGAAGGAGCTACTGGCAGCTCTGTACGGCGAGGTGACAATAAAAGAACTCGAGGAAACAAACCTCGGAGTCATCACCCCGGTTCGCGCGAACGAAAAGGTTACAATCACCCCTCTCCTACCCCCAAAAACTCAAAGCAGGGTCAGCTCCGTACTGAAGCGGTTCAGGAGCACCCGAAACACGGGGGGACTGCTTTCCGTAGAGAAAGTGGTGGTAGTGTTCACCCCTCACATCCCCGACGACGTGCTAGGAGAGGTGGAGATATGGCTCCACGACAGCATCCTCCCCCACCTCGGGAGCGTCGGACCAAGACTGAAACTCAAGCTGAGCGAAGGGCCCAAGCTCTTAGCGTTCTACCCACCCTACTCGATTGCATTGGGGGACTCGATCTCGGGCCAGCCGAGGTCCTTCTCCATTGTCACCGAGCTGTTCGAAGGCAACTTCGCACCGGGGTGCAGCCCATTCAGCCTGTTCCTCATGTGGAGTCCACGCATCGAAGCAGTGACCCACAACTACTTGAGTCGTCCACCACGTGCTCTGCCAATTTGCAGAACGATGGTGCGGGACGCGTTATCGGAGGTGGCATCCCAACAGCAATACCTGAAGGGAGCGATGTCGAACAGGTATGCCATGCCTCTCACTACGGGTGATGGCCAGCATAGAGCCATGAAGGGGGCTCCCAGTGCCCTTCCACCAACGGGGGTGTGTACCCAGGCTTCTAAGTGAGGCTTCGCTTCCCGCCGGAAGACCGCGGCGGTTCTGTTCCTCCCACAGGAGTACGGCAACAACCCACCTTGGGAAAGTGGGGACCCCAGCACTAACTCCTTTAACTAGGCGGGCGTGTTGGTTACAGTAGGAGGGGACAGTGCGCATCGAAACTGAGCCCCACCACAACTCTCATCCACGGGGTGGTTGGGACGCAGGTGTCGGAGGGATCGCCAGCCCTCAGGATAGTGAGCTCCCGCAGAGGGATAAGCTATCTCCCTGCGACGTAGTGGTAGAACACGTGGGATAGGGGATGACCTTGTCGACCGGTTATCGGTCCCCTGCTCCTTCGAGCTGGCAAGGCGCTCACAGGTTCTACACTGCTACTAAAGTTGGTGGTGGATGTCTCGCCCAAAAAGATCACAAACGCGCGGGACAAGGTCCCTTCCACCTTCGCCGGGTAAGGCTAGAGTCAGCGCTGCATGACTATAACTTGCGGCCGATCCAGTTGCACGACTGGTGGTCCCCCTCAGTGTCTCGGTTGTCTGCCGAGTGGGCGGTGGTCGGATTCCACCACACCCTGCCACGAGGTGCGTGGAGACTTGGCCAGTCTAGGCTCGTCGTAATTAGTTGCAGCGACGTTAATCAACCCGTCCGGGCATATAATAGGACCGGTTGTGCTTCTTCCTCCCTTCTTAGCCAGGTGGTTACCTCCCTGGCGCCC
