# Supplementary material for: Phase separation of a plant virus movement protein and cellular factors support virus-host interactions
Source: PLoS Pathog. 2021 Sep 20;17(9):e1009622. doi: 10.1371/journal.ppat.1009622 (PMC8483311; doi:10.1371/journal.ppat.1009622)
Supplement: S1 Table — Includes primers for cloning, in vitro transcription template preparation, RT-PCR, and RT-qPCR. (DOCX) [file ppat.1009622.s006.docx]

| **Primer Name** | **Sequence (5’ → 3’)** |
| --- | --- |
| **Primers for amplifying synthetic dsDNA fragments (gBlocks)** | |
| gBlock_Tag_F | GGCATGCATTCCGCAACGAG |
| gBlock_Tag_R | CGTCGCATCGCCGGATAATGG |
| **Primers for amplifying IDR_WT_ and Cterm** | |
| IDR_BamHI_F | GCAGCAGGATCCATGGCGGTAGGGAAATATATGACG |
| IDR_BamHI_R | TGCTGCGGATCCCCTTCGCTCAGCTTGAGTTTCAGTC |
| Cterm_NheI_F | GCAGCAGCTAGCGCCCAAGCTCTTAGCGTTCTAC |
| Cterm_-10aa_BamHI_R | TGCTGCGGATCCGACATCGCTCCCTTCAGGTATTGC |
| **Primers for amplifying fibrillarin (Fib2) sequences** | |
| At_Fib2_NheI_F | GCAGCAGCTAGCATGAGACCTCCTCTAACTGGAAGTGG |
| At_Fib2_BamHI_R | CTGCTGCGGATCCAGCAGCAGTAGCAGCCTTTGGCTTC |
| At_Fib2GAR_NheI_F | GCAGCAGCTAGCGGAAGTGGTGGTGGGTTCAGTGG |
| At_Fib2GAR_BamHI_R | TGCTGCGGATCCTCCTCCTTTCATGCCTCCACGGCC |
| **Primers for amplifying T7-Renilla luciferase sequence for**  **in vitro transcription** | |
| T7_RLuc_F | GGCTAGAGTACTTAATACGACTCACTATAGG |
| RLuc+13nt_3UTR_R | TCCTAGGGGCCCCTTACGTCGACATTTGTTC |
| **Primers for amplifying free GFP or p26:GFP fusions** | |
| p26_BamHI_F | GCAGCAGGATCCATGGCGGTAGGGAAATATATGACG |
| D/E-G_p26_BamHI_F | GCAGCAGGATCCACAATGGCTGTTGGAAAGTATATG |
| SalI_GFP_R | TGCTGCGTCGACCTATTTGTATAGTTCATCCATGCC |
| PacI_GFP_F | GCAGCAttaattaaACAATGAGTAAAGGAGAAGAAC |
| NotI_GFP_R | TGCTGCgcggccgcCTATTTGTATAGTTCATCCATG |
| **Primers for amplifying ΔNTF2-G3BP** | |
| ΔNTF2_BamHI_F | GCAGCAGGATCCATGGTTGATGAGGGTACTGTTTACTATC |
| RFP_SalI_R | TGCTGCGTCGACTTAGGCGCCGGTGGAGTGGC |
| **Primers for RT-PCR (TMV movement assay)** | |
| TMV_RT-PCR_F | CCGCGAATCTTATGTGGAAT |
| TMV_RT-PCR_R | TCCTCCAAGTGTTCCCAATC |
| Nb_actin_RT-PCR_F | TCCTGATGGGCAAGTGATTAC |
| Nb_actin RT-PCR_R | TTGTATGTGGTCTCGTGGATTC |
| **Primers for RT-qPCR (Real-time PCR)** | |
| Nb_G3BP_qPCR_F | TAGGGGAAGCAATCCAGATG |
| Nb_G3BP_qPCR_R | TCCTTATCGATCCCAACAGC |
| PEMV2_qPCR_F | TTGCAAGGTTCTAGGCATCC |
| PEMV2_qPCR_R | CAACGATCGAAAAAGACGATG |
| p14_qPCR_F | TCCCAAACAGGGGTTTTATG |
| p14_qPCR_R | GGTAATTGGGAACCCTCGAT |
