# Supplementary material for: Phase separation of a plant virus movement protein and cellular factors support virus-host interactions
Source: PLoS Pathog. 2021 Sep 20;17(9):e1009622. doi: 10.1371/journal.ppat.1009622 (PMC8483311; doi:10.1371/journal.ppat.1009622)
Supplement: S1 Materials and Methods — Details for Histidine-tag removal from IDRWT, FRAP analyses, image processing, and statistical analyses are included for data presented in supporting figures (S1–S4 Figs). (DOCX) [file ppat.1009622.s007.docx]

**MATERIALS & METHODS**

*Construction of IDR_R-K_ and IDR_VLIMFYW-S_ for bacterial expression*. All coding sequences, amino acid sequences, and cloning strategies are outlined in S1 Appendix. All DNA primer sequences used for cloning are listed in S1 Table. The IDR_R-K_ contains lysine (K) substitutions for all arginines (R) to prevent cation-pi interactions. IDR_VLIMFYW-S_ contains serine (S) substitutions for all hydrophobic residues to prevent hydrophobic interactions. R-K and VLIMFYW-S IDR’s were synthesized commercially as dsDNA fragments (Integrated DNA Technologies, Coralville, Iowa). Fragments were digested with BamHI and cloned into the BamHI site of pRSET his-eGFP (a gift from Jeanne Stachowiak, Addgene plasmid # 113551) to generate N-terminally his-tagged IDR-mutants fused to GFP. Constructs were sequenced to confirm direction and accuracy. Protein expression and purification followed the same protocol as used for IDR_WT_ as described in the main text.

*Histidine-tag removal using recombinant enterokinase*. Approximately 100 µg of His-tagged IDR_WT_ was mixed with 5 units of recombinant enterokinase (rEK, EMD Biosciences, San Diego, California) in 1X rEK cleavage buffer (50 mM NaCl, 20 mM Tris-HCl, 2 mM CaCl_2_, pH 7.4) and digested overnight at 4ºC. His-tag removal was confirmed by SDS-PAGE analysis and samples were concentrated and buffer-exchanged into storage buffer (10 mM Tris-HCl [pH 7.0], 300 mM NaCl, 1 mM EDTA, 1 mM dithiothreitol, and 10% glycerol Amicon 10K Ultra centrifugal filters. IDR_WT_ became inherently unstable after prolonged incubations at 4ºC in the presence or absence of rEK. After incubation, protein concentrations were re-evaluated using A_280_ readings on a UV5Nano spectrophotometer in addition to SDS-PAGE analyses.

*Confocal microscopy and FRAP*. IDR_WT_, IDR_R-K_, and IDR_VLIMFYW-S_ droplets were formed using the following mixture: 8 µM protein, 10 mM Tris-HCl (pH 7.5), 1 mM DTT, 100 mM NaCl, and 10% PEG-8000 to induce phase separation. Samples were directly loaded onto glass slides for confocal microscopy using a Zeiss LSM 510 Meta confocal microscope (488 nm) with a 20X objective and Zen 2009 software. Approximately ~2 µm diameter regions of IDR_WT_ droplets were photobleached with 100% laser power (488 nm) and fluorescence recovery was recorded at 5 s intervals.

*Image processing*. Total droplet areas (%) and droplet sizes were measured as outlined in the main text. To measure droplet/aggregate circularity, raw images (.lsm extension) were imported into ImageJ and thresholded. Condensates >2 µm^2^ in size were assessed using the “analyze particles” function with the “shape descriptors” option checked.

*Statistical analyses*. Total droplet areas (%), droplet sizes, turbidities were compared using statistical analyses as outlined in the main text. End-point FRAP recoveries were compared using an unpaired t test. Droplet/aggregate circularities were compared using a one-way ANOVA with Dunnett’s multiple comparisons test. All analyses were performed using GraphPad Prism software (version 9.0.1).
