# Supplementary material for: Phase separation of a plant virus movement protein and cellular factors support virus-host interactions
Source: PLoS Pathog. 2021 Sep 20;17(9):e1009622. doi: 10.1371/journal.ppat.1009622 (PMC8483311; doi:10.1371/journal.ppat.1009622)
Supplement: Attachment — Submitted filename: Response to Reviewers.pdf [file ppat.1009622.s008.pdf]

Part I - Summary
Please use this section to discuss strengths/weaknesses of study, novelty/significance, general execution and scholarship.

Reviewer #1: This paper introduces a really interesting new concept of phase separation playing a role in viral intercellular transport. The introduction explains that proteins containing intrinsically disordered regions self associate in oligomers, bind RNA, and phase separate when they are also enriched in arginine residues. The arginine residues are essential for cation-pi interactions with aromatic contacts to promote phase separation. Stress granules, the nucleolus represent examples of membraneless compartments in the cell and he suggests examples of cytoplasmic inclusions and some viral factores also aggregate as phase separation. This is a very interesting topic and this paper is the first to directly explore the concept for a plant virus, in this case PEMV2. The work is significant and novel and well executed overall. The in vitro turbidity assay and confocal microscopy are the strengths of the article, but there are some gaps when it comes to exlaining the various mutants in Figure2. Figure 2 is the most critical figure to the paper and there is room for improvement for this paper to be published. The M&M also needs to be better organized to match the order of the results, and the refererence list needs to be reviewed and edited for style.

Reviewer #2: In this manuscript, Brown and May present exciting data illustrating the phase separation of a viral protein and a host protein that participates in host-virus interactions. The viral protein, P26, participates in the phase separation in the nucleus with fibrillarin to support the systemic movement of viruses. P26 also phase separates together with a stress granule marker (G3BP) to limit viral accumulations when over-expressed. The study provides a timely update for the mechanistic understanding of host-virus interactions, thus fitting the scope of PLoS Pathogens.

Reviewer #3: This work presents a biochemical characterization of pea enation mosaic virus movement protein p26, which has an intrinsically disordered region with several charge amino acids at its N-terminal part. It belongs to proteins that can undergo phase separation both in vitro and in vivo. This property is convincingly demonstrated by many methods. With mutants having either all positively or all negatively charged amino acids of the N-terminal part substituted with glycine residues, the authors show that positive charges are required both for phase separation property and nuclear localization. Negative charges could be changed without affecting these functions, but the behavior of this protein in nucleus was altered. It's association with nucleolus was prolonged which was presented as the possible reason for its failure to complement long-distance movement function of a movement-deficient TMV. The authors investigated the associations of p26 protein with fibrillarin and viral RNA and propose an interplay between these as an enabler of systemic movement. The participation of nucleolus and fibrillarin together with GRV, an umbravirus, movement protein has previously been studied in detail. The authors also predict an antiviral role for association between PEMV p26 and G3PB, which is manifested as a reduced PEMV accumulation upon G3BP upregulation. This subject should be studied further to demonstrate how G3BP actually interferes with PEMV 2 infection.

Part II – Major Issues: Key Experiments Required for Acceptance
Please use this section to detail the key new experiments or modifications of existing experiments that should be absolutely required to validate study conclusions.

Reviewer #1: Page 6 line 123—should explain that the in vitro assays start with gene expression in E. coli and explain what the assays are. The M&M does not have a subtitle for In vitro phase separation assays, so it is not explained there either.
A short description of the phase separation assays has been added on Page 6 that reads "In vitro assays consisted of inducing phase separation of recombinant proteins with 10% PEG-8000 and observing phase separation via confocal microscopy or monitoring the solution turbidity (OD600)." E. coli expression has been mentioned in the text on page 6 as well. The M&M section now has a section titled "in vitro phase separation assays" that describes confocal microscopy, turbidity assays, and mean condensate size measurements.

Also, figure 2C is a Coomassie gel, so I think the results may be an immunoprecipitation?
Figure 2C is a Coomassie gel showing the relative purity and MW's of recombinant proteins used in this study. The Fig. 2 legend now states "Recombinant proteins used in this study were analyzed by SDS-PAGE to assess size and purity."

The M&M suggest the constructs have His Tag and so does this impact the IDR assays because of their charged sidechains. This is very important to address.
We thank the reviewer for raising this point and we have extensively examined the impact of the His-tag on IDR assays. Supplemental Fig. 1 now shows purified IDR-GFP with cleaved his-tag (Panel A). Importantly, cleaved IDR-GFP and tagged IDR-GFP behaved identically in confocal microscopy phase separation assays, turbidity assays, droplet size assays, sensitivity towards 10% 1,6 hexanediol, and RNA sorting assays. The only difference we found was FRAP recovery of IDR-GFP increased following tag removal (Panel F). Therefore, FRAP analyses of His-tagged proteins have been removed from the manuscript. Since the goal of our mutational analyses was to identify phase separation-deficient mutants (not study droplet dynamics), the use of His-tagged constructs was suitable since the His-tag did not influence phase separation propensity. Similar findings have been made for the SARS-CoV-2 N protein and this has been referenced in the text.

All IDR constructs are inherently unstable and difficult to purify. Cleaving the His-tag requires extended incubation periods at 4C for enterokinase reactions and reduces protein stability. Therefore, we favored maintaining protein integrity over altered FRAP dynamics. Finally, the R/K-G, D/E-G, and ΔNLS mutants behaved identically both in vitro (his-tagged) and in vivo (untagged) demonstrating the his-tagged proteins' behavior in vitro is a strong indicator of their behavior in plants.

Notably PEG is used to precipitate proteins by absorbing water and so I would suggest that this is a turbidity assay, not necessarily functioning as a mimic of cell crowding. I suggest rephrasing lines 13-131 on page 6.
The text suggesting PEG-8000 mimics cell crowding has been replaced with text simply stating "In vitro assays consisted of inducing phase separation of recombinant proteins with 10% PEG-8000"

Did free GFP also have the HisTag? Each lane in Fig 2C needs to be explained, for example what is R/K-G? I think the real in vitro assay is Figure 2D, not 2C.
All bacterially expressed recombinant proteins used in this study contained an N-terminal histidine-tag. This is now clearly stated on page 6 "Note: all constructs presented in Figure 2 contain N-terminal (His)tidine tags since the presence of a His-tag did not influence IDR-GFP phase separation propensity, particle size, or resistance to 1,6-hexanediol that selectively dissolves liquid condensates [46] (Supplemental Fig. 1A-E)."

The following phrase has been added to the M&M section: "Histidine-tagged recombinant proteins were expressed in BL21(DE3) E. coli".

We have better described each construct in Fig. 2. Furthermore, we have moved the R-K and VLIMFYW-S mutants to the supplemental figures since these constructs showed no change compared to IDR-GFP. We believe this simplifies the text and increases readability.

Importantly the authors indicate that IDR by itself is responsible for phase separation, but it would be useful to have additional segmental mutations to show that the non-IDR region is not responsible for phase separation.
We thank the reviewer for this suggestion. We have added a new panel to Fig 2A showing the predicted phase separation propensity of p26 using the catGRANULE algorithm. The C-terminal half of p26 is not predicted to drive phase separation and was fused to GFP (C-term construct). The last 10 amino acids of the C-terminus had to be omitted because they led to cleavage during E. coli expression. This has been described in the M&M section.

Importantly, the C-term was unable to drive phase separation when viewed by confocal microscopy or turbidity assays. This data has been added to Fig. 2.

The R-K, VLIMFYW-S, R/K-G, and D/E-G are not defined in M&M or Figure 2 legend or results and these are central to testing the hypothesis.
All mutations have now been described in the M&M sections as well as the Fig. 2 and Supplemental Fig. 3 figure legends.

The R-K and VLIMFYW-S data has been moved to supplemental figure 3. R-K and VLIMFYW-S showed no difference versus IDR-GFP in phase separation propensity. Their mutations are now better defined on page 7 (bottom).

The R/K-G and D/E-G mutations have been described on Page 7 (Lines 149-151).

Figure 2D is to show turbidity. An important control that is missing is the non-IDR region fused to GFP.
The updated turbidity assays include the C-term fused to GFP that fails to phase separate (Fig. 2E).

The IDR-GFP fusion is not as green as GFP alone. Since I don't know what R-K, VLIMFYW-S, R/K-G, and D/E-G, I am also wondering why these are not included in Figure 2D. What if you mixed other proteins or RNA into the in vitro system? Why not add the salt and PEG into the tubes in panel D as in panel E? Figure 2E is referred to as in vitro assay but it seems to be in vivo? OR is this solution placed on a slide?
Fig. 2E (now Fig. 2D) was confocal microscopy of *in vitro* phase separation assays. Turbidity assays are now presented for **ALL** constructs tested in this study. Fig. 2E shows the turbidity assays (8 uM and 24 uM protein) for GFP, IDR-GFP, C-term, R/K-G, D/E-G, and ΔNLS.

Confocal microscopy was used for the salt assays since the confocal microscopy is far superior in sensitivity and dynamic range. Using 8 uM protein, the turbidity of IDR-GFP is <0.2. Therefore, there is little range for seeing decreases in turbidity. However, using confocal microscopy, large differences in phase separation were observed with 1 M NaCl.

Supplemental Fig. 3 contains turbidity assays for R-K and VLIMFYW-S (GFP and IDR-GFP are included for comparison).

The order of M&M sections should match the order of the results. The constructs start with the E coli expression vectors, but Figure 1 is Agro-infiltration of 35S plasmids. not E coli and I am not sure what the delivery is.
The order of the M&M section has been shuffled to coincide with the order experiments are presented in the results section. However, the first section "construction of binary plant expression vectors" contains information for all constructs used in subsequent figures to prevent having multiple sections for binary vector construction.
The delivery of expression vectors for all figures is now explicitly stated in both the text and figure legends to avoid confusion of 35S driven, TMV driven, etc.

It is not clear to me in Figure 1 and 2 if the p26 gene fusions are introduced into leaves via TMV vector or agro-delivery of plasmids. Lines 368 and 385 are contradictory—regarding synthesis and cloning. I suggest removing redundancies that may be confusing.
Fig. 1 legend now states "Free GFP and p26 C-terminally fused with GFP (p26:GFP) were expressed from binary expression plasmids under the constitutive CaMV 35S promoter. (B) Following agroinfiltration of N. benthamiana…"

The M&M section has been clarified regarding all types of constructs used in this study including pBIN binary expression vectors, pJL-TRBO TMV vectors, or pRSET bacterial expression vectors. All three types have a dedicated section in the M&M.

Pager 7 lines 144-146 describe Fig 2C which is out of order. Need to move the mutations up into the prior section and discuss all Figure 2 in one section of results.
Negative results have been moved to supplemental Fig. 3 in order to simplify Fig. 2. All mutations are now described together with IDR-GFP in a single panel (Fig. 2D). All panels are discussed in the order presented in Fig. 2.

Figure 3 is robust. But page 11 discusses figures out of order. I think this is confusing. Figure 4 shows P26-GFP complements movement defects of TMV which is a very important set of experiments to include.
All figures are now described in order. The earlier work studying GRV or PEMV2 (May et. al, 2020) has been referenced to show that p26:GFP could support TMV movement. This now reads "Interestingly, GRV pORF3 and PEMV2 p26 can systemically traffic TMV when expressed from a subgenomic promoter in place of CP [42, 59]."

Reviewer #2: Based on the presented data, P26 appears to facilitate viral systemic trafficking when phase separating with fibrillarin in the nucleus while phase separates with G3BP in cytoplasmic stress granule that seems to inhibit viral replication. But the data were all based on protein over-expressing. It will be informative to understand the P26 partition in the nuclear and cytoplasmic compartments in native infection conditions to quantitatively accessing the role of P26 in viral infection.
While much of our data is from p26 overexpression using the CaMV 35S promoter, our results expressing p26:GFP, R/K-G, or D/E-G proteins during a TMV infection are the same. In other

words, p26:GFP and D/E-G nuclear localization patterns are unchanged during TMV infection versus 35S-driven expression. Furthermore, R/K-G remains diffusely expressed in the cytoplasm irrespective of 35S promoter or TMV-driven expression. Data directly comparing nuclear localization of p26:GFP and D/E-G p26 is now included in Fig. 6C. No significant changes in nuclear retention of D/E-G were observed during virus infection supporting our conclusion that increased nucleolar retention of D/E-G is at least partially responsible for the observed block in virus movement.

While we agree with this reviewer that it would be useful to observe p26 localization patterns using authentic PEMV2 infections, PEMV2 will not tolerate addition of a fluorescent reporter (i.e. GFP) and we do not have p26 antibodies available (and additional antibodies for D/E-G would likely be required). Since p26 can systemically traffic TMV, we believe the TMV infection system is a suitable model for studying p26 biology.

Reviewer #3: 1. Specificity of p26 functions in virus infection remains hard to interpret. Does its nucleolar and stress granule partitioning with fibrillarin and G3BP occur in a specific manner or is it typical for proteins with this kind of properties to co-aggregate at certain concentrations. Are there specific interactions of p26 with either Fib or G3BP?
The related GRV ORF3 protein is known to interact with Fib2 through the Fib2 GAR domain. We have cited this research to justify our use of the Fib2GAR protein for partitioning assays with IDR-GFP. This reads as follows: "Fib2GAR was chosen since the related GRV pORF3 directly interacts with the Fib2 GAR domain [35]."

No known interactions exist between p26 and G3BP. However, G3BP condensates can contain hundreds of cellular proteins and we are not surprised that p26 can co-localize with G3BP since both proteins are RNA binding proteins that phase separate.

It is well established that proteins that undergo phase separation partition in shared phase separations as many of these proteins bind RNAs non-specifically and will co-localize after forming RNA-protein phase separations.

How is the selection of viral RNA done for long distance movement? Both cognate and non-cognate viral RNAs condensate with p26-GFP. Would any RNA condensate? The experimental design does not allow to make conclusions of how p26 works in PEMV infection.
RNA sorting assays with preformed IDR-GFP droplets were repeated with Cy5-labelled Renilla luciferase RNAs. This data has been added to Fig. 5B and Fig. 5C. Importantly, RLuc RNAs were sorted to IDR-GFP droplets with similar efficiency compared to the viral PEMV2 and TCV RNAs. Therefore, any RNA seemingly has the potential to partition in p26 droplets.

2. The mutants used in this study are very robust. Changing all positively and all negatively charged amino acids to glycine alters the protein products drastically. The different functions p26 has in long distance movement (phase separation, nuclear localization, retention in the nucleus, protein-protein and protein-RNA interactions etc.) may become impossible to separate from each other.

I suggest that the specificity of p26 IDR region interactions be investigated by subtle mutations, and especially in the natural context of PEMV infection, to understand the requirements of PEMV long-distance transport.
We thank the reviewer for their suggestion in trying more subtle arginine mutations. We agree that the drastic R/K-G mutation that was necessary for blocking phase separation made separating the role of phase separation from nuclear localization or virus movement difficult.

Very few proteins that phase separate (if any) can be prevented from phase separating through small deletions or a small number of substitutions. The most well-studied proteins like FUS and FMRP require large deletions (~200 amino acids) to block phase separation. TDP-43 requires substitution of hydrophobic residues to block phase separation (e.g. VLIMFYW-S).

However, we engineered a new mutation into the IDR that deleted the conserved nuclear localization signal (ΔNLS). This data has been added to Fig. 2 and Fig. 3. This mutation removed 5 arginine residues in a 6 amino acid tract (RRRARR). ΔNLS phase separated with equal propensity to wild-type IDR but was unable to partition in the nucleolus. Therefore, we concluded that phase separation alone is not sufficient for nucleolar trafficking (and subsequent virus movement). Rather, we suggest that phase separation is required to enter preformed Fib2 droplets in the nucleolus but requires a NLS that is not necessary for phase separation to reach the nucleus.

In regard to examining p26 IDR mutations in the context of natural infections, we encountered major roadblocks. First, PEMV2 will not tolerate the addition of fluorescent reporters to visualize p26 phase separation during PEMV2 infection. Next, the p26 ORF is overlapping with the p27 movement protein and mutation of p26 will simultaneously disrupt p27, adding additional variables to potential experiments. Using TMV as a virus vector to determine if p26 or mutants can complement systemic movement was in our opinion our best option for observing the effects of p26 mutations on virus movement.

Part III – Minor Issues: Editorial and Data Presentation Modifications
Please use this section for editorial suggestions as well as relatively minor modifications of existing data that would enhance clarity.

Reviewer #1: Please review the References page and fix the style.
The TMV work is an important complementation experiment showing the fusion protein functions and that the fusion is not malformed. I think this is important to state.
The following text has been added to page 11 and reads "Furthermore, p26 can systemically traffic TMV when expressed in place of CP from a subgenomic promoter [60] and remains functional when fused to GFP [42]."

Reviewer #2: 1) Phase separation-deficient G3BP already restricted viral accumulation up to 5 folds, which is already very efficient. One interpretation of data is that phase separation, in this case, enhances the inhibitory role of G3BP in viral infection. G3BP has other intrinsic activity to sufficiently suppress viral accumulation.
We thank the reviewer for their insight and interpretation of this data. We agree that this description of G3BP antiviral activity better describes the role of phase separation in this process. The introduction and results sections now have sentences that state "phase separation enhances antiviral activity of G3BP towards PEMV2"

2) Some rationales behind the experimental designs should be explained. For example, why particularly 1:6 molar ratio was used in line 224? There are numerous cases like this throughout the manuscript.
The 1:6 ratio was used since scaffold proteins (Fib2) must be at a higher concentration than client proteins (p26) for partitioning in pre-formed droplets to occur. The following description has been added to page 10: " Fib2 functions as a scaffold for recruiting client proteins into the phase separated nucleolus, and by nature, scaffolds should be present in excess relative to clients for partitioning to occur [56, 57]. Thus, a 1:6 molar ratio of p26:Fib2GAR was used in the following experiments."

Descriptions describing the rationales for using 1:500 RNA:protein molar ratios and 500:500:1 p26:Fib2:RNA ratios has been added to page 10 as well and read:

“Cy5-labelled PEMV2 RNA was mixed with pre-formed Fib2GAR or Fib2FL droplets at a 1:500 RNA:Fib2 molar ratio. This ratio was used since earlier work determined that umbravirus RNAs were saturated by protein interactors under these conditions [33, 41].”

and

“Droplets containing equimolar Fib2FL and IDR-GFP were pre-formed prior to the addition of PEMV2-Cy5 RNAs at a 1:500 RNA:protein molar ratio. Equimolar amounts of Fib2FL and IDR-GFP were used since atomic force microscopy revealed that Fib2 and GRV pORF3 form ring-like complexes with equimolar composition [32].”

3) It is relevant to include a recent reference in discussion (Pubmed ID: 33910901).
Reference has been added to the first paragraph of the discussion section.

Reviewer #3: Page 4 rows 84-88: PEMV 2 is a virus… in family Tombusviridae? Please, remind readers that taxonomically both PEMV 2 and GRV belong to genus Umbravirus of family Tombusviridae.
The text has been modified to read “Pea enation mosaic virus 2 (PEMV2) is a small (4,252 nt), positive-sense RNA plant virus belonging to the Tombusviridae family and umbravirus genus.” and “Both p26 and the closely related umbravirus orthologue pORF3 from Groundnut rosette virus (GRV) …”

Page 6 row 130-131: it is stated that phase separation of IDR-GFP phase separation under crowding conditions could be observed by turbidity assay (Fig. 2D). Unfortunately, the quality of the Fig. 2D does not allow to see this.
We agree the turbidity is difficult to see and is the result of the rather low turbidity values (~0.2). We have now included Turbidity assays (OD600 readings) for all IDR-GFP constructs examined in this study and are shown in Fig. 2E and Supplemental Fig. 3B. The initial photo has been removed.

Page 7 row 151: I don’t understand how the mean condensate sizes of all the other mutants are very similar except D/E-G. If I look the confocal image in 2E and 2G, I see differences. It would be good to explain which protein concentration was used to calculate this result.
Thank you for pointing out this discrepancy. All condensate sizes were measured under standard assay conditions using 8 µM protein. Indeed, when tripling the D/E-G concentration to 24 µM, the droplet sizes are comparable to IDR-GFP at 8 µM, but this concentration was not used for these measurements.

The main text now only includes condensate sizes for the three proteins that phase separated, including IDR-GFP, D/E-G, and ΔNLS. The supplemental data shows condensate sizes for IDR-GFP, R-K, and VLIMFYW-S mutants. Supplemental Fig. 2 shows IDR-GFP, R/K-G, and D/E-G condensates at 24 uM to demonstrate R/K-G forms irregular shaped aggregates whereas IDR-GFP and D/E-G form droplets.

Page 8 row 171: Please, explain what the basis to state is that the marked structures are nucleolus and Cajal bodies in the Fig. 3A. Did you use some markers here?
We labelled the largest, densely stained body in the nucleus as the nucleolus. We agree without using Nucleolus-specific markers that it is not possible to label the nucleolus or cajal bodies with

100% accuracy. Therefore, we have removed the nucleolus labels and refer to these regions as “Nuclear Bodies” in the Figure 3 legend. The white arrows now only point to nuclear bodies that contain p26. This general description serves our purpose for labelling NBs that co-localize with p26.

Page 12 row 285-188: The authors need to show that deltaNTF1-G3BP and G3BP are expressed on the same level (Fig. 5E) to make the conclusion that phase separation is needed for the full recovery of PEMV accumulation.
Western blots are now included (from the original samples) in Fig. 7E. Both full-length G3BP and NTF2 deletion proteins are expressed at similar levels.
