# Supplementary material for: Phase separation of a plant virus movement protein and cellular factors support virus-host interactions
Source: PLoS Pathog. 2021 Sep 20;17(9):e1009622. doi: 10.1371/journal.ppat.1009622 (PMC8483311; doi:10.1371/journal.ppat.1009622)
Supplement: Attachment — Submitted filename: PPATHOGENS-D-21-00877_R1.pdf [file ppat.1009622.s009.pdf]

PLOS Pathogens

Phase separation of both a plant virus movement protein and cellular factors support virus-host interactions

--Manuscript Draft--

Manuscript Number:	PPATHOGENS-D-21-00877R1
Full Title:	Phase separation of both a plant virus movement protein and cellular factors support virus-host interactions
Short Title:	Plant virus protein phase separation
Article Type:	Research Article
Section/Category:	Virology
Keywords:	phase separation, LLPS, liquid-liquid phase separation, RNA virus, stress granule, biomolecular condensate, fibrillarin, virus movement, virus replication, G3BP, virus-host interaction, plant virus, RNA, nucleolus
Abstract:	Phase separation concentrates biomolecules, which should benefit RNA viruses that must sequester viral and host factors during an infection. Here, the p26 movement protein from *Pea enation mosaic virus 2* (PEMV2) was found to phase separate and partition in nucleoli and G3BP stress granules (SGs) *in vivo*. Electrostatic interactions drive p26 phase separation as mutation of basic (R/K-G) or acidic (D/E-G) residues either blocked or reduced phase separation, respectively. During infection, p26 must partition inside the nucleolus and interact with fibrillarin (Fib2) as a pre-requisite for systemic trafficking of viral RNAs. Partitioning of p26 in pre-formed Fib2 droplets was dependent on p26 phase separation suggesting that phase separation supports a critical virus-host interaction required for virus movement. Furthermore, viral ribonucleoprotein complexes containing p26, Fib2, and PEMV2 RNAs were formed via phase separation *in vitro* and could provide the basis for self-assembly *in planta*. Interestingly, both R/K-G and D/E-G p26 mutants failed to support systemic trafficking of a *Tobacco mosaic virus* (TMV) vector in *Nicotiana benthamiana* suggesting that p26 phase separation, proper nucleolar partitioning, and systemic movement are intertwined. p26 also partitioned in SGs and G3BP over-expression restricted PEMV2 accumulation >20-fold. Expression of phase separation-deficient G3BP only restricted PEMV2 5-fold, demonstrating that phase separation enhances G3BP antiviral activity.
Additional Information:	
Question	Response
**Financial Disclosure**<br><br>Enter a financial disclosure statement that describes the sources of funding for the work included in this submission. Review the submission guidelines for detailed requirements. View published research articles from *PLOS Pathogens* for specific examples.<br><br>This statement is required for submission and **will appear in the published article** if the submission is accepted. Please make sure it is accurate.	This work was supported by University of Missouri-Kansas City (UMKC) institutional start-up funds to J.P.M.<br><br>The sponsors had no role in study design, data collection and analysis, decision to publish, or preparation of the manuscript.

Powered by Editorial Manager® and ProduXion Manager® from Aries Systems Corporation

**Unfunded studies**<br>Enter: *The author(s) received no specific funding for this work.*<br><br>**Funded studies**<br><br>Enter a statement with the following details:<br>• Initials of the authors who received each award<br>• Grant numbers awarded to each author<br>• The full name of each funder<br>• URL of each funder website<br>• Did the sponsors or funders play any role in the study design, data collection and analysis, decision to publish, or preparation of the manuscript?<br>• **NO** - Include this sentence at the end of your statement: *The funders had no role in study design, data collection and analysis, decision to publish, or preparation of the manuscript.*<br>• **YES** - Specify the role(s) played.<br><br>* typeset	
**Competing Interests**<br><br>Use the instructions below to enter a competing interest statement for this submission. On behalf of all authors, disclose any competing interests that could be perceived to bias this work—acknowledging all financial support and any other relevant financial or non-financial competing interests.<br><br>This statement **will appear in the published article** if the submission is accepted. Please make sure it is accurate. View published research articles from *PLOS Pathogens* for specific examples.	The authors have declared that no competing interests exist.

**NO authors have competing interests**<br><br>Enter: *The authors have declared that no competing interests exist.*<br><br>**Authors with competing interests**<br><br>Enter competing interest details beginning with this statement:<br><br>*I have read the journal's policy and the authors of this manuscript have the following competing interests: [insert competing interests here]*<br><br>* typeset<br><br>This statement is required for submission and **will appear in the published article** if the submission is accepted. Please make sure it is accurate and that any funding sources listed in your Funding Information later in the submission form are also declared in your Financial Disclosure statement.	
**Data Availability**<br><br>Authors are required to make all data underlying the findings described fully available, without restriction, and from the time of publication. PLOS allows rare exceptions to address legal and ethical concerns. See the PLOS Data Policy and FAQ for detailed information.<br><br>A Data Availability Statement describing where the data can be found is required at submission. Your answers to this question constitute the Data Availability Statement and **will be published in the article**, if accepted.<br><br>**Important:** Stating 'data available on request from the author' is not sufficient. If your data are only available upon request, select 'No' for the first question and explain your exceptional situation in the text box.	Yes - all data are fully available without restriction

Do the authors confirm that all data underlying the findings described in their manuscript are fully available without restriction?	
**Describe where the data may be found in full sentences. If you are copying our sample text, replace any instances of XXX with the appropriate details.**<br>• If the data are **held or will be held in a public repository**, include URLs, accession numbers or DOIs. If this information will only be available after acceptance, indicate this by ticking the box below. For example: *All XXX files are available from the XXX database (accession number(s) XXX, XXX.*).<br>• If the data are all contained **within the manuscript and/or Supporting Information files**, enter the following: *All relevant data are within the manuscript and its Supporting Information files.*<br>• If neither of these applies but you are able to provide **details of access elsewhere**, with or without limitations, please do so. For example:<br><br>*Data cannot be shared publicly because of [XXX]. Data are available from the XXX Institutional Data Access / Ethics Committee (contact via XXX) for researchers who meet the criteria for access to confidential data.*<br><br>*The data underlying the results presented in the study are available from (include the name of the third party and contact information or URL).*<br>• This text is appropriate if the data are owned by a third party and authors do not have permission to share the data.<br><br>* typeset	All relevant data are within the manuscript and its Supporting Information files.
Additional data availability information:	

Part I - Summary
Please use this section to discuss strengths/weaknesses of study, novelty/significance, general execution and scholarship.

Reviewer #1: This paper introduces a really interesting new concept of phase separation playing a role in viral intercellular transport. The introduction explains that proteins containing intrinsically disordered regions self associate in oligomers, bind RNA, and phase separate when they are also enriched in arginine residues. The arginine residues are essential for cation-pi interactions with aromatic contacts to promote phase separation. Stress granules, the nucleolus represent examples of membraneless compartments in the cell and he suggests examples of cytoplasmic inclusions and some viral factores also aggregate as phase separation. This is a very interesting topic and this paper is the first to directly explore the concept for a plant virus, in this case PEMV2. The work is significant and novel and well executed overall. The in vitro turbidity assay and confocal microscopy are the strengths of the article, but there are some gaps when it comes to exlaining the various mutants in Figure2. Figure 2 is the most critical figure to the paper and there is room for improvement for this paper to be published. The M&M also needs to be better organized to match the order of the results, and the refererence list needs to be reviewed and edited for style.

Reviewer #2: In this manuscript, Brown and May present exciting data illustrating the phase separation of a viral protein and a host protein that participates in host-virus interactions. The viral protein, P26, participates in the phase separation in the nucleus with fibrillarin to support the systemic movement of viruses. P26 also phase separates together with a stress granule marker (G3BP) to limit viral accumulations when over-expressed. The study provides a timely update for the mechanistic understanding of host-virus interactions, thus fitting the scope of PLoS Pathogens.

Reviewer #3: This work presents a biochemical characterization of pea enation mosaic virus movement protein p26, which has an intrinsically disordered region with several charge amino acids at its N-terminal part. It belongs to proteins that can undergo phase separation both in vitro and in vivo. This property is convincingly demonstrated by many methods. With mutants having either all positively or all negatively charged amino acids of the N-terminal part substituted with glycine residues, the authors show that positive charges are required both for phase separation property and nuclear localization. Negative charges could be changed without affecting these functions, but the behavior of this protein in nucleus was altered. It’s association with nucleolus was prolonged which was presented as the possible reason for its failure to complement long-distance movement function of a movement-deficient TMV. The authors investigated the associations of p26 protein with fibrillarin and viral RNA and propose an interplay between these as an enabler of systemic movement. The participation of nucleolus and fibrillarin together with GRV, an umbravirus, movement protein has previously been studied in detail. The authors also predict an antiviral role for association between PEMV p26 and G3PB, which is manifested as a reduced PEMV accumulation upon G3BP upregulation. This subject should be studied further to demonstrate how G3BP actually interferes with PEMV 2 infection.

Part II – Major Issues: Key Experiments Required for Acceptance
Please use this section to detail the key new experiments or modifications of existing experiments that should be absolutely required to validate study conclusions.

Reviewer #1: Page 6 line 123—should explain that the in vitro assays start with gene expression in E. coli and explain what the assays are. The M&M does not have a subtitle for In vitro phase separation assays, so it is not explained there either.
A short description of the phase separation assays has been added on Page 6 that reads "In vitro assays consisted of inducing phase separation of recombinant proteins with 10% PEG-8000 and observing phase separation via confocal microscopy or monitoring the solution turbidity (OD600)." E. coli expression has been mentioned in the text on page 6 as well.
The M&M section now has a section titled "in vitro phase separation assays" that describes confocal microscopy, turbidity assays, and mean condensate size measurements.

Also, figure 2C is a Coomassie gel, so I think the results may be an immunoprecipitation?
Figure 2C is a Coomassie gel showing the relative purity and MW's of recombinant proteins used in this study. The Fig. 2 legend now states "Recombinant proteins used in this study were analyzed by SDS-PAGE to assess size and purity."

The M&M suggest the constructs have His Tag and so does this impact the IDR assays because of their charged sidechains. This is very important to address.
We thank the reviewer for raising this point and we have extensively examined the impact of the His-tag on IDR assays. Supplemental Fig. 1 now shows purified IDR-GFP with cleaved his-tag (Panel A). Importantly, cleaved IDR-GFP and tagged IDR-GFP behaved identically in confocal microscopy phase separation assays, turbidity assays, droplet size assays, sensitivity towards 10% 1,6 hexanediol, and RNA sorting assays. The only difference we found was FRAP recovery of IDR-GFP increased following tag removal (Panel F). Therefore, FRAP analyses of His-tagged proteins have been removed from the manuscript. Since the goal of our mutational analyses was to identify phase separation-deficient mutants (not study droplet dynamics), the use of His-tagged constructs was suitable since the His-tag did not influence phase separation propensity. Similar findings have been made for the SARS-CoV-2 N protein and this has been referenced in the text.

All IDR constructs are inherently unstable and difficult to purify. Cleaving the His-tag requires extended incubation periods at 4C for enterokinase reactions and reduces protein stability. Therefore, we favored maintaining protein integrity over altered FRAP dynamics. Finally, the R/K-G, D/E-G, and ΔNLS mutants behaved identically both in vitro (his-tagged) and in vivo (untagged) demonstrating the his-tagged proteins' behavior in vitro is a strong indicator of their behavior in plants.

Notably PEG is used to precipitate proteins by absorbing water and so I would suggest that this is a turbidity assay, not necessarily functioning as a mimic of cell crowding. I suggest rephrasing lines 13-131 on page 6.
The text suggesting PEG-8000 mimics cell crowding has been replaced with text simply stating "In vitro assays consisted of inducing phase separation of recombinant proteins with 10% PEG-8000"

Did free GFP also have the HisTag? Each lane in Fig 2C needs to be explained, for example what is R/K-G? I think the real in vitro assay is Figure 2D, not 2C.
All bacterially expressed recombinant proteins used in this study contained an N-terminal histidine-tag. This is now clearly stated on page 6 "Note: all constructs presented in Figure 2 contain N-terminal (His)tidine tags since the presence of a His-tag did not influence IDR-GFP phase separation propensity, particle size, or resistance to 1,6-hexanediol that selectively dissolves liquid condensates [46] (Supplemental Fig. 1A-E)."

The following phrase has been added to the M&M section: "Histidine-tagged recombinant proteins were expressed in BL21(DE3) E. coli".

We have better described each construct in Fig. 2. Furthermore, we have moved the R-K and VLIMFYW-S mutants to the supplemental figures since these constructs showed no change compared to IDR-GFP. We believe this simplifies the text and increases readability.

Importantly the authors indicate that IDR by itself is responsible for phase separation, but it would be useful to have additional segmental mutations to show that the non-IDR region is not responsible for phase separation.
We thank the reviewer for this suggestion. We have added a new panel to Fig 2A showing the predicted phase separation propensity of p26 using the catGRANULE algorithm. The C-terminal half of p26 is not predicted to drive phase separation and was fused to GFP (C-term construct). The last 10 amino acids of the C-terminus had to be omitted because they led to cleavage during E. coli expression. This has been described in the M&M section.

Importantly, the C-term was unable to drive phase separation when viewed by confocal microscopy or turbidity assays. This data has been added to Fig. 2.

The R-K, VLIMFYW-S, R/K-G, and D/E-G are not defined in M&M or Figure 2 legend or results and these are central to testing the hypothesis.
All mutations have now been described in the M&M sections as well as the Fig. 2 and Supplemental Fig. 3 figure legends.

The R-K and VLIMFYW-S data has been moved to supplemental figure 3. R-K and VLIMFYW-S showed no difference versus IDR-GFP in phase separation propensity. Their mutations are now better defined on page 7 (bottom).

The R/K-G and D/E-G mutations have been described on Page 7 (Lines 149-151).

Figure 2D is to show turbidity. An important control that is missing is the non-IDR region fused to GFP.
The updated turbidity assays include the C-term fused to GFP that fails to phase separate (Fig. 2E).

The IDR-GFP fusion is not as green as GFP alone. Since I don't know what R-K, VLIMFYW-S, R/K-G, and D/E-G, I am also wondering why these are not included in Figure 2D. What if you mixed other proteins or RNA into the in vitro system? Why not add the salt and PEG into the tubes in panel D as in panel E? Figure 2E is referred to as in vitro assay but it seems to be in vivo? OR is this solution placed on a slide?
Fig. 2E (now Fig. 2D) was confocal microscopy of *in vitro* phase separation assays. Turbidity assays are now presented for **ALL** constructs tested in this study. Fig. 2E shows the turbidity assays (8 uM and 24 uM protein) for GFP, IDR-GFP, C-term, R/K-G, D/E-G, and ΔNLS.

Confocal microscopy was used for the salt assays since the confocal microscopy is far superior in sensitivity and dynamic range. Using 8 uM protein, the turbidity of IDR-GFP is <0.2. Therefore, there is little range for seeing decreases in turbidity. However, using confocal microscopy, large differences in phase separation were observed with 1 M NaCl.

Supplemental Fig. 3 contains turbidity assays for R-K and VLIMFYW-S (GFP and IDR-GFP are included for comparison).

The order of M&M sections should match the order of the results. The constructs start with the E coli expression vectors, but Figure 1 is Agro-infiltration of 35S plasmids. not E coli and I am not sure what the delivery is.
The order of the M&M section has been shuffled to coincide with the order experiments are presented in the results section. However, the first section "construction of binary plant expression vectors" contains information for all constructs used in subsequent figures to prevent having multiple sections for binary vector construction.
The delivery of expression vectors for all figures is now explicitly stated in both the text and figure legends to avoid confusion of 35S driven, TMV driven, etc.

It is not clear to me in Figure 1 and 2 if the p26 gene fusions are introduced into leaves via TMV vector or agro-delivery of plasmids. Lines 368 and 385 are contradictory—regarding synthesis and cloning. I suggest removing redundancies that may be confusing.
Fig. 1 legend now states "Free GFP and p26 C-terminally fused with GFP (p26:GFP) were expressed from binary expression plasmids under the constitutive CaMV 35S promoter. (B) Following agroinfiltration of N. benthamiana…"

The M&M section has been clarified regarding all types of constructs used in this study including pBIN binary expression vectors, pJL-TRBO TMV vectors, or pRSET bacterial expression vectors. All three types have a dedicated section in the M&M.

Pager 7 lines 144-146 describe Fig 2C which is out of order. Need to move the mutations up into the prior section and discuss all Figure 2 in one section of results.
Negative results have been moved to supplemental Fig. 3 in order to simplify Fig. 2. All mutations are now described together with IDR-GFP in a single panel (Fig. 2D). All panels are discussed in the order presented in Fig. 2.

Figure 3 is robust. But page 11 discusses figures out of order. I think this is confusing. Figure 4 shows P26-GFP complements movement defects of TMV which is a very important set of experiments to include.
All figures are now described in order. The earlier work studying GRV or PEMV2 (May et. al, 2020) has been referenced to show that p26:GFP could support TMV movement. This now reads "Interestingly, GRV pORF3 and PEMV2 p26 can systemically traffic TMV when expressed from a subgenomic promoter in place of CP [42, 59]."

Reviewer #2: Based on the presented data, P26 appears to facilitate viral systemic trafficking when phase separating with fibrillarin in the nucleus while phase separates with G3BP in cytoplasmic stress granule that seems to inhibit viral replication. But the data were all based on protein over-expressing. It will be informative to understand the P26 partition in the nuclear and cytoplasmic compartments in native infection conditions to quantitatively accessing the role of P26 in viral infection.
While much of our data is from p26 overexpression using the CaMV 35S promoter, our results expressing p26:GFP, R/K-G, or D/E-G proteins during a TMV infection are the same. In other

words, p26:GFP and D/E-G nuclear localization patterns are unchanged during TMV infection versus 35S-driven expression. Furthermore, R/K-G remains diffusely expressed in the cytoplasm irrespective of 35S promoter or TMV-driven expression. Data directly comparing nuclear localization of p26:GFP and D/E-G p26 is now included in Fig. 6C. No significant changes in nuclear retention of D/E-G were observed during virus infection supporting our conclusion that increased nucleolar retention of D/E-G is at least partially responsible for the observed block in virus movement.

While we agree with this reviewer that it would be useful to observe p26 localization patterns using authentic PEMV2 infections, PEMV2 will not tolerate addition of a fluorescent reporter (i.e. GFP) and we do not have p26 antibodies available (and additional antibodies for D/E-G would likely be required). Since p26 can systemically traffic TMV, we believe the TMV infection system is a suitable model for studying p26 biology.

Reviewer #3: 1. Specificity of p26 functions in virus infection remains hard to interpret. Does its nucleolar and stress granule partitioning with fibrillarin and G3BP occur in a specific manner or is it typical for proteins with this kind of properties to co-aggregate at certain concentrations. Are there specific interactions of p26 with either Fib or G3BP?
The related GRV ORF3 protein is known to interact with Fib2 through the Fib2 GAR domain. We have cited this research to justify our use of the Fib2GAR protein for partitioning assays with IDR-GFP. This reads as follows: "Fib2GAR was chosen since the related GRV pORF3 directly interacts with the Fib2 GAR domain [35]."

No known interactions exist between p26 and G3BP. However, G3BP condensates can contain hundreds of cellular proteins and we are not surprised that p26 can co-localize with G3BP since both proteins are RNA binding proteins that phase separate.

It is well established that proteins that undergo phase separation partition in shared phase separations as many of these proteins bind RNAs non-specifically and will co-localize after forming RNA-protein phase separations.

How is the selection of viral RNA done for long distance movement? Both cognate and non-cognate viral RNAs condensate with p26-GFP. Would any RNA condensate? The experimental design does not allow to make conclusions of how p26 works in PEMV infection.
RNA sorting assays with preformed IDR-GFP droplets were repeated with Cy5-labelled Renilla luciferase RNAs. This data has been added to Fig. 5B and Fig. 5C. Importantly, RLuc RNAs were sorted to IDR-GFP droplets with similar efficiency compared to the viral PEMV2 and TCV RNAs. Therefore, any RNA seemingly has the potential to partition in p26 droplets.

2. The mutants used in this study are very robust. Changing all positively and all negatively charged amino acids to glycine alters the protein products drastically. The different functions p26 has in long distance movement (phase separation, nuclear localization, retention in the nucleus, protein-protein and protein-RNA interactions etc.) may become impossible to separate from each other.

I suggest that the specificity of p26 IDR region interactions be investigated by subtle mutations, and especially in the natural context of PEMV infection, to understand the requirements of PEMV long-distance transport.
We thank the reviewer for their suggestion in trying more subtle arginine mutations. We agree that the drastic R/K-G mutation that was necessary for blocking phase separation made separating the role of phase separation from nuclear localization or virus movement difficult.

Very few proteins that phase separate (if any) can be prevented from phase separating through small deletions or a small number of substitutions. The most well-studied proteins like FUS and FMRP require large deletions (~200 amino acids) to block phase separation. TDP-43 requires substitution of hydrophobic residues to block phase separation (e.g. VLIMFYW-S).

However, we engineered a new mutation into the IDR that deleted the conserved nuclear localization signal (ΔNLS). This data has been added to Fig. 2 and Fig. 3. This mutation removed 5 arginine residues in a 6 amino acid tract (RRRARR). ΔNLS phase separated with equal propensity to wild-type IDR but was unable to partition in the nucleolus. Therefore, we concluded that phase separation alone is not sufficient for nucleolar trafficking (and subsequent virus movement). Rather, we suggest that phase separation is required to enter preformed Fib2 droplets in the nucleolus but requires a NLS that is not necessary for phase separation to reach the nucleus.

In regard to examining p26 IDR mutations in the context of natural infections, we encountered major roadblocks. First, PEMV2 will not tolerate the addition of fluorescent reporters to visualize p26 phase separation during PEMV2 infection. Next, the p26 ORF is overlapping with the p27 movement protein and mutation of p26 will simultaneously disrupt p27, adding additional variables to potential experiments. Using TMV as a virus vector to determine if p26 or mutants can complement systemic movement was in our opinion our best option for observing the effects of p26 mutations on virus movement.

Part III – Minor Issues: Editorial and Data Presentation Modifications
Please use this section for editorial suggestions as well as relatively minor modifications of existing data that would enhance clarity.

Reviewer #1: Please review the References page and fix the style.
The TMV work is an important complementation experiment showing the fusion protein functions and that the fusion is not malformed. I think this is important to state.
The following text has been added to page 11 and reads “Furthermore, p26 can systemically traffic TMV when expressed in place of CP from a subgenomic promoter [60] and remains functional when fused to GFP [42].”

Reviewer #2: 1) Phase separation-deficient G3BP already restricted viral accumulation up to 5 folds, which is already very efficient. One interpretation of data is that phase separation, in this case, enhances the inhibitory role of G3BP in viral infection. G3BP has other intrinsic activity to sufficiently suppress viral accumulation.
We thank the reviewer for their insight and interpretation of this data. We agree that this description of G3BP antiviral activity better describes the role of phase separation in this process. The introduction and results sections now have sentences that state “phase separation enhances antiviral activity of G3BP towards PEMV2”

2) Some rationales behind the experimental designs should be explained. For example, why particularly 1:6 molar ratio was used in line 224? There are numerous cases like this throughout the manuscript.
The 1:6 ratio was used since scaffold proteins (Fib2) must be at a higher concentration than client proteins (p26) for partitioning in pre-formed droplets to occur. The following description has been added to page 10: ” Fib2 functions as a scaffold for recruiting client proteins into the phase separated nucleolus, and by nature, scaffolds should be present in excess relative to clients for partitioning to occur [56, 57]. Thus, a 1:6 molar ratio of p26:Fib2GAR was used in the following experiments.”

Descriptions describing the rationales for using 1:500 RNA:protein molar ratios and 500:500:1 p26:Fib2:RNA ratios has been added to page 10 as well and read:

“Cy5-labelled PEMV2 RNA was mixed with pre-formed Fib2GAR or Fib2FL droplets at a 1:500 RNA:Fib2 molar ratio. This ratio was used since earlier work determined that umbravirus RNAs were saturated by protein interactors under these conditions [33, 41].”

and

“Droplets containing equimolar Fib2FL and IDR-GFP were pre-formed prior to the addition of PEMV2-Cy5 RNAs at a 1:500 RNA:protein molar ratio. Equimolar amounts of Fib2FL and IDR-GFP were used since atomic force microscopy revealed that Fib2 and GRV pORF3 form ring-like complexes with equimolar composition [32].”

3) It is relevant to include a recent reference in discussion (Pubmed ID: 33910901).
Reference has been added to the first paragraph of the discussion section.

Reviewer #3: Page 4 rows 84-88: PEMV 2 is a virus… in family Tombusviridae? Please, remind readers that taxonomically both PEMV 2 and GRV belong to genus Umbravirus of family Tombusviridae.
The text has been modified to read “Pea enation mosaic virus 2 (PEMV2) is a small (4,252 nt), positive-sense RNA plant virus belonging to the Tombusviridae family and umbravirus genus.” and “Both p26 and the closely related umbravirus orthologue pORF3 from Groundnut rosette virus (GRV) …”

Page 6 row 130-131: it is stated that phase separation of IDR-GFP phase separation under crowding conditions could be observed by turbidity assay (Fig. 2D). Unfortunately, the quality of the Fig. 2D does not allow to see this.
We agree the turbidity is difficult to see and is the result of the rather low turbidity values (~0.2). We have now included Turbidity assays (OD600 readings) for all IDR-GFP constructs examined in this study and are shown in Fig. 2E and Supplemental Fig. 3B. The initial photo has been removed.

Page 7 row 151: I don’t understand how the mean condensate sizes of all the other mutants are very similar except D/E-G. If I look the confocal image in 2E and 2G, I see differences. It would be good to explain which protein concentration was used to calculate this result.
Thank you for pointing out this discrepancy. All condensate sizes were measured under standard assay conditions using 8 µM protein. Indeed, when tripling the D/E-G concentration to 24 µM, the droplet sizes are comparable to IDR-GFP at 8 µM, but this concentration was not used for these measurements.

The main text now only includes condensate sizes for the three proteins that phase separated, including IDR-GFP, D/E-G, and ΔNLS. The supplemental data shows condensate sizes for IDR-GFP, R-K, and VLIMFYW-S mutants. Supplemental Fig. 2 shows IDR-GFP, R/K-G, and D/E-G condensates at 24 uM to demonstrate R/K-G forms irregular shaped aggregates whereas IDR-GFP and D/E-G form droplets.

Page 8 row 171: Please, explain what the basis to state is that the marked structures are nucleolus and Cajal bodies in the Fig. 3A. Did you use some markers here?
We labelled the largest, densely stained body in the nucleus as the nucleolus. We agree without using Nucleolus-specific markers that it is not possible to label the nucleolus or cajal bodies with

100% accuracy. Therefore, we have removed the nucleolus labels and refer to these regions as "Nuclear Bodies" in the Figure 3 legend. The white arrows now only point to nuclear bodies that contain p26. This general description serves our purpose for labelling NBs that co-localize with p26.

Page 12 row 285-188: The authors need to show that deltaNTF1-G3BP and G3BP are expressed on the same level (Fig. 5E) to make the conclusion that phase separation is needed for the full recovery of PEMV accumulation.
Western blots are now included (from the original samples) in Fig. 7E. Both full-length G3BP and NTF2 deletion proteins are expressed at similar levels.

Manuscript
Click here to access/download;Manuscript;PLoS_Pathogens_REVISION_PLA

Phase separation of both a plant virus movement protein and cellular factors support virus-host interactions

Shelby L. Brown[1], Dana J. Garrison[1], and Jared P. May[1*]

[1]Department of Cell and Molecular Biology and Biochemistry, School of Biological and Chemical Sciences, University of Missouri-Kansas City, Kansas City, MO 64110, USA

*Correspondence: jpmay@umkc.edu

Short title: Plant virus protein phase separation

Keywords: phase separation, LLPS, liquid-liquid phase separation, RNA virus, stress granule, biomolecular condensate, fibrillarin, virus movement, virus replication, G3BP, virus-host interaction, plant virus, RNA, nucleolus

ABSTRACT

Phase separation concentrates biomolecules, which should benefit RNA viruses that must sequester viral and host factors during an infection. Here, the p26 movement protein from *Pea enation mosaic virus 2* (PEMV2) was found to phase separate and partition in nucleoli and G3BP stress granules (SGs) *in vivo*. Electrostatic interactions drive p26 phase separation as mutation of basic (R/K-G) or acidic (D/E-G) residues either blocked or reduced phase separation, respectively. During infection, p26 must partition inside the nucleolus and interact with fibrillarin (Fib2) as a pre-requisite for systemic trafficking of viral RNAs. Partitioning of p26 in pre-formed Fib2 droplets was dependent on p26 phase separation suggesting that phase separation supports a critical virus-host interaction required for virus movement. Furthermore, viral ribonucleoprotein complexes containing p26, Fib2, and PEMV2 RNAs were formed via phase separation *in vitro* and could provide the basis for self-assembly *in planta*. Interestingly, both R/K-G and D/E-G p26 mutants failed to support systemic trafficking of a *Tobacco mosaic virus* (TMV) vector in *Nicotiana benthamiana* suggesting that p26 phase separation, proper nucleolar partitioning, and systemic movement are intertwined. p26 also partitioned in SGs and G3BP over-expression restricted PEMV2 accumulation >20-fold. Expression of phase separation-deficient G3BP only restricted PEMV2 5-fold, demonstrating that phase separation enhances G3BP antiviral activity.

AUTHOR SUMMARY

Phase separation of several cellular proteins is associated with forming pathological aggregates and exacerbating neurodegenerative disease progression. In contrast, roles for viral protein phase separation in RNA virus lifecycles are less understood. Here, we demonstrate that the p26 movement protein from *Pea enation mosaic virus 2* phase separates and partitions with phase-separated cellular proteins fibrillarin and G3BP. The related orthologue from *Groundnut rosette virus* has been extensively studied and is known to interact with fibrillarin in

the nucleolus as a pre-requisite for virus movement. We determined that basic residues and electrostatic interactions were critical for p26 phase separation and partitioning in pre-formed fibrillarin droplets. Furthermore, mutation of charged residues prevented p26 from complementing a movement-deficient *Tobacco mosaic virus* vector in *Nicotiana benthamiana*. ~~Stress granules form through phase separation and~~ we found that p26 partitions inside stress granules following heat shock. Phase separation of the stress granule nucleator G3BP was required for maximum antiviral activity and constitutes a host response that requires phase separation. In summary, we found that phase separation of p26 and G3BP is necessary for pro-viral and anti-viral activities, respectively.

INTRODUCTION

Cellular organelles are membrane-bound compartments that are critical for eukaryotic cell function and RNA viruses often co-opt organelles to promote virus replication. Organelles exploited by RNA viruses include the endoplasmic reticulum (ER) [1], mitochondria [2], nucleus [3], and Golgi apparatus [4]. Recently, much attention has been directed towards membraneless organelles that form through protein phase separation. Phase separation transforms a single-phase solution into a dilute phase and droplet phase that concentrates biomolecules, such as proteins or RNAs [5, 6]. Some cellular proteins phase separate and form aggregates that are associated with several neurodegenerative disorders [7]. Proteins that undergo phase separation consistently contain intrinsically disordered regions (IDRs) that self-associate to form oligomers [8]. Many IDR-containing proteins have RNA-recognition motifs that non-specifically bind RNA and fine-tune phase separation by controlling material exchange, shape, and rigidity of liquid droplets [8, 9]. Proteins that phase separate are often enriched in arginine residues that promote phase separation through cation-pi interactions with aromatic contacts [10]. In addition, hydrophobic interactions can stabilize phase separations of low-complexity domains [11].

Membraneless organelles exist as liquids, gels, or solids, [12]. The most notable examples of liquid-liquid phase separated (LLPS) membraneless compartments are the nucleolus and cytoplasmic P-bodies [13]. Less dynamic stress granules (SGs) also form in the cytoplasm through phase separation and allow host cells to repress translation and influence messenger RNA (mRNA) stability in response to various stresses [14]. SGs are visible by microscopy within minutes following stress and contain Ras-GTPase-activating protein SH3 domain-binding protein 1 (G3BP1) that self-associates to induce SG formation [15]. SGs contain a stable inner core and an outer shell that is formed by weak electrostatic and/or hydrophobic interactions [16]. The G3BP1 inner core is resistant to dilution (atypical for LLPS) and has been regarded as a form of liquid-solid demixing [17]. Interestingly, G3BP1 can have either pro-viral [18-20] or anti-viral roles [21-23] in RNA virus lifecycles.

Members of the *Mononegavirales*, including *Rabies virus*, *Measles virus* (MeV), and *Vesicular stomatitis virus* generate phase-separated cytoplasmic inclusion bodies that harbor viral factories [24-26]. Phase separation of MeV N and P proteins also promotes efficient encapsidation of viral RNAs [26]. Several groups have recently demonstrated that the nucleocapsid (N) protein from the novel SARS-CoV-2 coronavirus undergoes LLPS [27]. SARS-CoV-2 N protein phase separation is stimulated by the 5' end of its cognate RNA [28] and can partition into phase separations of heterogeneous nuclear ribonucleoproteins like TDP-43, FUS, and hnRNPA2 [29]. N protein phase separation has also been suggested to mediate nucleocapsid assembly and genome processing [30]. Finally, N protein interacts with G3BP1 and can attenuate SG formation [31, 32].

Pea enation mosaic virus 2 (PEMV2) is a small (4,252 nt), positive-sense RNA plant virus belonging to the *Tombusviridae* family and umbravirus genus. The PEMV2 long-distance movement protein p26 is required for systemic trafficking of viral RNA throughout an infected plant. Both p26 and the closely related umbravirus orthologue pORF3 from *Groundnut rosette virus* (GRV) primarily localize to the cytoplasm, but also target cajal bodies in the nucleus and

eventually partition in the nucleolus [33-35]. Umbravirus ORF3 proteins must interact with nucleolar fibrillarin, a pre-requisite for long-distance movement of viral RNA [35-37]. Additionally, the polerovirus *Potato leafroll virus* (PLRV) and the potexvirus *Bamboo mosaic virus* satellite RNA (satBaMV) encode proteins that must also localize to the nucleolus and interact with fibrillarin to support systemic movement [38-40]. Fibrillarin phase separates and forms the dense fibrillar component (DFC) of the nucleolus that shares a similar structure to SGs [16, 41]. Although the nucleolus itself is a phase separation and several plant virus proteins interact with fibrillarin, the role of viral protein phase separation in plant virus lifecycles has not been investigated.

This study demonstrates that PEMV2 p26 undergoes phase separation both *in vitro* and *in vivo* and forms poorly dynamic condensates. Viral ribonucleoprotein (vRNP) complexes containing p26, fibrillarin, and PEMV2 RNAs were reconstituted *in vitro* through phase separation and could represent the version of the *in vivo* event necessary for systemic trafficking. Charged residues played critical roles in p26 phase separation, nucleolar localization, and movement of a virus vector suggesting that phase separation and virus movement are intertwined. Finally, p26 partitions in G3BP SGs and G3BP over-expression exhibits strong antiviral activity towards PEMV2. Virus accumulation was largely restored during expression of a phase separation-deficient G3BP, demonstrating that phase separation enhances G3BP antiviral activity.

RESULTS

p26 forms poorly dynamic condensates *in vivo*. ~~PEMV2 p26 and related umbravirus orthologues form large cytoplasmic granules during infection [37, 42, 43].~~ To visualize p26 granules, green fluorescent protein (GFP) was fused to the C-terminus of full-length p26 and expressed from the *Cauliflower mosaic virus* (CaMV) 35S promoter following agroinfiltration of *Nicotiana benthamiana* leaves (Fig. 1A). As a control, free GFP was expressed from the CaMV

35S promoter and failed to form granules but was evenly distributed throughout the cytoplasm and nucleus of the cell (i.e, outside of the large vacuole that comprises most of the cellular space) (Fig. 1B, Left). However, p26:GFP formed large cytoplasmic granules as previously observed (Fig. 1B, Right) [43]. To define the material properties of p26 granules *in vivo*, we used fluorescence recovery after photobleaching (FRAP) [44]. If p26 granules are highly dynamic liquid droplets, then FRAP recovery should be rapid and complete. Conversely, if p26 granules are solid aggregates, no fluorescence recovery is expected. Interestingly, p26:GFP granules recovered nearly 50% by 30 seconds post-bleach (Fig. 1C) demonstrating that p26 droplets have measurable fluidity. However, since p26:GFP failed to fully recover, our data suggests that p26 forms poorly dynamic condensates *in vivo* similar to what has been observed for G3BP1 SG cores [17].

p26 is intrinsically disordered and undergoes phase separation via electrostatic interactions. Since IDRs typically drive phase separation, the IUPred prediction model [45] was used to identify an arginine-rich disordered region spanning amino acids 1-132 of p26 (Fig. 2A, Top). The same region was also predicted to have the highest propensity to phase separate using the catGRANULE algorithm that was trained to identify proteins known to form nuclear or cytoplasmic foci (Fig. 2A, Bottom) [46]. To confirm the p26 IDR drives phase separation and subsequently identify mutations that block phase separation, the p26 IDR or a set of IDR mutants were fused to the N-terminus of GFP and purified from *Escherichia coli* (Fig. 2B and C). *In vitro* assays consisted of inducing phase separation of recombinant proteins with 10% PEG-8000 and observing droplet formation via confocal microscopy or measuring the solution turbidity (OD_{600}). Expectedly, wild-type IDR-GFP readily phase separated as observed by both confocal microscopy (Fig. 2D) and turbidity assays (Fig. 2E). In contrast, both free GFP and GFP fused to the C-terminal region of p26 (amino acids 133-226) failed to phase separate ~~under all tested conditions~~ (Fig. 2D and E). Note: all constructs presented in Figure 2 contain N-terminal (His)tidine tags since the presence of a His-tag did not influence IDR-GFP phase

separation propensity, particle size, or resistance to 1,6-hexanediol that selectively dissolves liquid condensates [47] (Supplemental Fig. 1A-E). Similar observations have been reported for His-tagged and tag-free SARS-CoV-2 N protein [28]. Surprisingly, FRAP recovery of IDR-GFP dramatically increased following His-tag removal suggesting that histidine tracts ~~can~~ influence droplet dynamics *in vitro* (Supplemental Fig. 1F).

Electrostatic interactions support both protein self-association and phase separation but can be inhibited by high salt concentrations [48]. Therefore, to determine whether p26 phase separation is driven by electrostatic interactions, phase separation assays were performed with 1 M NaCl. Significantly reduced phase separation of IDR-GFP was observed by confocal microscopy (Fig. 2D) and 600 mM NaCl was sufficient to block IDR-GFP phase separation near the saturation concentration (C_{sat} = 2 μM) (Fig. 2F). To confirm electrostatic interactions drive p26 phase separation, all basic or acidic residues were mutated to glycine (R/K-G or D/E-G, respectively). Indeed, R/K-G failed to phase separate while D/E-G showed significantly reduced phase separation compared to IDR-GFP when examined by confocal microscopy (Fig. 2D), turbidity assays (Fig. 2E), or mean condensate size (Fig. 2G). At higher concentrations (24 μM), R/K-G formed non-uniform aggregates, whereas D/E-G formed uniform droplets (Supplemental Fig. 2). A more subtle mutation was tested by deleting the sequence 5'-RRRARR-3' (amino acids 100-105) that constitutes a conserved nuclear localization signal (NLS) first identified in GRV pORF3 [49]. ΔNLS phase separated with equal propensity to wild-type (Fig. 2D and E) demonstrating that the highly conserved NLS is not required for phase separation. This finding is somewhat unsurprising since the NLS only accounts for 16% (5/31) of the basic residues within the IDR. Finally, potential cation-pi or hydrophobic interactions were disrupted by mutating all arginines to lysines (R-K) or all hydrophobic residues to serine (VLIMFYW-S), respectively. Both R-K and VLIMFYW-S mutants phase separated with equal propensity to wild-type demonstrating cation-pi and hydrophobic interactions are not required for p26 phase

separation (Supplemental Fig. 3). Together, these results demonstrate that the N-terminal IDR drives p26 phase separation through electrostatic interactions.

Charged residues govern p26 nucleolar partitioning. Umbravirus movement proteins must access the nucleolus to support systemic virus trafficking [35]. Here, the nucleolar partitioning of full-length wild-type or mutant p26:GFP was examined after agroinfiltration of *N. benthamiana* leaves with constructs expressing p26:GFP from a CaMV 35S promoter. As previously reported for related orthologues [35-37, 49], p26 was observed in nuclear bodies (e.g. nucleolus) in addition to forming cytoplasmic granules appearing as droplets (Fig. 3A). Supporting our *in vitro* observations, full-length p26 containing glycine substitutions for all basic residues (R/K-G) did not form phase-separated granules but instead was diffusely expressed throughout the cytoplasm and failed to partition in the nucleolus (Fig. 3A). Expectedly, deletion of the conserved NLS resulted in strictly cytoplasmic localization of p26. Since ΔNLS formed phase-separated droplets but failed to enter the nucleolus, our data demonstrates that phase separation of p26 alone is insufficient for nucleolar localization. Despite reduced phase separation of the D/E-G IDR *in vitro*, full-length p26 containing glycine substitutions for all acidic residues (D/E-G) formed cytoplasmic granules that appeared like wild-type (Fig. 3A). However, 33% of D/E-G granules localized to the nucleus compared to only 5% of wild-type p26 granules (Fig. 3B) suggesting that the net charge of p26 influences nucleolar localization. The overall net charge of D/E-G at pH 7.4 is +36 compared to +14 for wild-type and our findings support earlier work that showed nucleolar localization of cellular and viral proteins was dependent on the overall positive charge [50, 51].

p26 phase separation is required for partitioning in Fib2 droplets. Fibrillarin (Fib2) is a host factor required for systemic trafficking of umbravirus vRNPs [33, 34] and makes up the dense fibrillar component of the nucleolus [52]. The *A. thaliana* Fib2 N-terminus contains an intrinsically disordered glycine- and arginine-rich (GAR) domain (Fig. 4A) that is common to fibrillarin across eukaryotes [53]. To determine whether the GAR domain of *A. thaliana* Fib2 is

sufficient for Fib2 phase separation, the GAR domain (amino acids 7-77, Fib2$_{GAR}$) was fused to the N-terminus of mCherry and purified from *E. coli* for *in vitro* phase separation assays (Fig. 4B). Full-length Fib2 was also fused to mCherry (Fib2$_{FL}$) for comparison. Free mCherry did not phase separate in the presence of 10% PEG-8000 or under high-salt conditions (Fig. 4C). Fib2$_{GAR}$ readily phase separated under crowding conditions but was unable to phase separate in the presence of 1 M NaCl (Fig. 4C). These results indicate that the GAR domain is sufficient to drive Fib2 phase separation through electrostatic interactions and is consistent with findings using mammalian or *Caenorhabditis elegans* fibrillarin [41, 54, 55]. Full-length Fib2 phase separated under crowding conditions but unlike Fib2$_{GAR}$, Fib2$_{FL}$ was resistant to 1 M NaCl (Fig. 4C). These results suggest that Fib2$_{FL}$ condensates are not strictly dependent on electrostatic interactions or Fib2$_{FL}$ can form salt-resistant aggregates.

During an infection, p26 must presumably partition in pre-formed Fib2 droplets in the dense fibrillar component of the nucleolus [41] to support virus movement. Therefore, we sought to determine whether phase separation of p26 was required for partitioning in Fib2 droplets. Fib2 functions as a *scaffold* for recruiting *client* proteins into the phase separated nucleolus, and by nature scaffolds should be present in excess relative to clients for partitioning to occur [56, 57]. Thus, a 1:6 molar ratio of IDR-GFP:Fib2$_{GAR}$ was used in the following experiments. Fib2$_{GAR}$ was chosen since the related GRV pORF3 directly interacts with the Fib2 GAR domain [36]. Expectedly, IDR-GFP was readily sorted into pre-formed Fib2$_{GAR}$ droplets *in vitro* (Fig. 4D, Left) and is likely the reconstituted version of the p26-Fib2 interaction required for Fib2 export from the nucleus and subsequent association with viral RNAs [35]. To determine whether phase separation of p26 was required for Fib2 partitioning, the phase separation-deficient R/K-G mutant was added to pre-formed Fib2$_{GAR}$ droplets. Interestingly, R/K-G remained in the bulk phase and was excluded from Fib2$_{GAR}$ droplets (Fig. 4D, Right, White arrows). These results demonstrate that p26 phase separation is critical for interactions with phase-separated Fib2 and strongly support a role for phase separation in PEMV2 movement.

vRNPs required for systemic trafficking can be reconstituted via phase separation. Movement-competent umbravirus vRNPs consist of Fib2, p26, and genomic RNAs [36]. Therefore, we sought to determine whether vRNPs could be re-constituted *in vitro* through phase separation. First, to determine whether full-length PEMV2 RNA could be sorted to Fib2 droplets, Cy5-labelled PEMV2 RNA was mixed with pre-formed $Fib2_{GAR}$ or $Fib2_{FL}$ droplets at a 1:500 RNA:Fib2 molar ratio. This ratio was used since earlier work determined that umbravirus RNAs were saturated by protein interactors under these conditions [34, 42]. PEMV2-Cy5 RNA was not efficiently sorted into $Fib2_{GAR}$ droplets (Fig. 5A) and is consistent with earlier findings that determined the GAR domain does not bind RNA [53, 54]. However, $Fib2_{FL}$ efficiently captured PEMV2-Cy5 RNAs demonstrating that PEMV2 RNAs can partition in Fib2 phase separations (Fig. 5A). Since p26 must also associate with viral RNAs, PEMV2-Cy5 RNAs were mixed with pre-formed IDR-GFP droplets again using a 1:500 RNA:protein ratio that saturates viral RNA with p26. Approximately 50% of IDR-GFP signal spatially overlapped PEMV2-Cy5 signal when visualized by confocal microscopy and quantified by MOC (Fig. 5B and C). Interestingly, partitioning of RNAs inside IDR-GFP condensates was not unique to PEMV2 RNAs since the distantly related *Turnip crinkle virus* (TCV) and non-viral *Renilla* luciferase (RLuc) RNAs were sorted to IDR-GFP phase separations with equal propensity (Fig. 5B and C). Importantly, the N-terminal His-tag of IDR-GFP did not influence RNA sorting into droplets (Supplemental Fig. 1G). Finally, equimolar $Fib2_{FL}$ and IDR-GFP were mixed with PEG to form droplets prior to the addition of PEMV2-Cy5 RNAs at a 1:500 molar ratio. Equimolar amounts of $Fib2_{FL}$ and IDR-GFP were used since atomic force microscopy revealed that Fib2 and GRV pORF3 form ring-like complexes with equimolar composition [33]. Droplets containing IDR-GFP, $Fib2_{FL}$, and PEMV2 RNAs were observed (Fig. 5D) and demonstrates that movement-competent vRNPs can be reconstituted by phase separation *in vitro*. Together, these findings suggest that phase separation of Fib2 and p26 ~~could~~ support the formation of movement-competent vRNPs *in planta*.

Phase separation-deficient p26 mutants fail to systemically traffic a virus vector.

To determine whether phase separation-deficient p26 mutants could support virus trafficking, a movement-deficient *Tobacco mosaic virus* (TMV) vector was used to express free GFP, p26, R/K-G, or D/E-G GFP fusions (Fig. 6A). The TMV vector (pJL-TRBO) contains a coat protein (CP) deletion that has been previously reported to block systemic movement [58]. However, previous work has demonstrated that GRV pORF3 can support long-distance movement of TMV when co-expressed alongside a movement-deficient TMV vector [59]. Furthermore, both native p26 and p26:GFP can systemically traffic TMV when expressed from a subgenomic promoter in place of CP [43, 60]. Local infections were established in young *N. benthamiana* plants (4th leaf stage) and high levels of free GFP and lower levels of p26:GFP, R/K-G, and D/E-G were observed at 4 days post-infiltration (dpi) (Fig. 6B). Localization patterns of p26:GFP, R/K-G, and D/E-G did not differ when expressed from either a 35S promoter or a TMV vector and confirmed that D/E-G granules were significantly enriched in nuclei compared to wild-type p26 during virus infection (Fig. 6C). As expected, systemic movement of TMV by p26:GFP was readily apparent by 14 dpi by both visual inspection of leaves and RT-PCR whereas free GFP did not move TMV systemically (Fig. 6D). Since R/K-G p26 can neither phase separate nor enter the nucleolus, R/K-G expectedly failed to systemically traffic TMV at 14 dpi (Fig. 6D). Surprisingly, D/E-G p26 also failed to support TMV movement at 14 dpi despite the ability to phase separate (albeit less efficiently *in vitro*) and localize to the nucleolus. However, drastically increased nucleolar retention of D/E-G likely contributed to the block in systemic movement and suggests that nucleolar and virus trafficking by p26 is a tightly regulated process. Together, these data suggest that p26 phase separation, nucleolar partitioning, and virus movement are connected and co-dependent on charged residues. The TMV CP deletion has been previously reported to block systemic movement of the TRBO vector [58], but we routinely observed systemic trafficking of pJL-GFP after 3 weeks (Supplemental Fig. 4). However, pJL-GFP was largely

restricted to the petiole and midrib of systemic leaves whereas pJL-p26:GFP spread throughout the veins and invaded the lamina.

p26 is sorted into G3BP phase separations that restrict PEMV2 accumulation*.* Our findings suggest that p26 phase separations are poorly dynamic and share similar material properties to G3BP SG cores [17]. Since SGs can have both pro-viral and antiviral roles in RNA lifecycles, we investigated whether p26 could partition in G3BP SGs. A NTF2-RRM domain-containing protein from *A. thaliana* (AtG3BP) functions as a G3BP-like SG nucleator in plants [61]. In mammals, the N-terminal NTF2 domain (Fig. 7A) is required for both phase separation and recruitment to SGs [62, 63]. As previously demonstrated by Krapp et. al. [61], G3BP:RFP displays a diffuse cytoplasmic expression pattern under no stress, but forms cytoplasmic SGs after heat shock (Fig. 7B). As expected, ΔNTF2-G3BP failed to phase separate and form SGs following heat shock (Fig. 7B). When co-expressed with p26:GFP, recruitment of p26 to G3BP SGs was observed following heat shock (Fig. 7B)~~ demonstrating that p26 can partition in phase-separated SGs.~~ To determine whether p26 partitions into SGs during a viral infection*,* G3BP:RFP was agroinfiltrated into *N. benthamiana* plants systemically infected with TMV expressing p26:GFP (Fig. 7C). p26:GFP condensates co-localized with G3BP:RFP demonstrating that p26 and G3BP can share phase separations during an authentic viral infection (Fig. 7C). Next, native G3BP expression was measured by RT-qPCR at 3 dpi in PEMV2-infected *N. benthamiana* leaves and revealed a 61% increase during infection that could be part the anti-viral host response (Fig. 7D). To confirm G3BP has an inhibitory effect on PEMV2 accumulation, G3BP:RFP was co-infiltrated with PEMV2 into *N. benthamiana*. At 3 dpi, PEMV2 accumulation was reduced >20-fold by G3BP over-expression demonstrating that G3BP exerts strong antiviral activity towards PEMV2 (Fig. 7E). Virus accumulation was largely restored (only 5-fold inhibition) during overexpression of ΔNTF2-G3BP indicating that phase separation of G3BP is required for maximal antiviral activity (Fig. 7E). Together, these data

demonstrate that p26 partitions inside G3BP SGs and phase separation of G3BP enhances antiviral activity towards PEMV2.

DISCUSSION

Phase separation of viral proteins has largely been associated with negative-sense RNA viruses that use phase separation to form virus factories [26], including Negri bodies during Rabies virus infections [24, 64, 65]. In contrast, many positive-strand RNA viruses, including members of the *Tombusviridae* family form membranous replication organelles to concentrate virus replication complexes [66, 67]. Although limited evidence for phase separation of plant virus proteins exists [68], a recent study demonstrated that *Turnip mosaic virus* inhibits the formation of phase-separated nuclear dicing bodies (D-bodies) that are responsible for microRNA processing and anti-viral defense [69, 70]. While these findings demonstrate plant viruses ~~have~~ evolved to suppress certain cellular phase separations, examples of plant virus proteins using phase separation to support virus-host interactions have not been reported.

This study demonstrates that the N-terminal IDR of p26 drives phase separation of poorly dynamic condensates through electrostatic interactions. Phase separation of p26 was abolished by mutating all basic residues to glycine (R/K-G) both *in vitro* and *in vivo*. Surprisingly, mutation of acidic residues (D/E-G) did not abolish phase separation but was significantly reduced *in vitro* compared to wild-type. Previous studies have found that phase separation of arginine-rich peptides can occur through charge repulsion in the presence of buffer counteranions and could explain D/E-G phase separation [71, 72]. Mutation of charged residues resulted in altered nucleolar localization of p26. Both deletion of the conserved p26 NLS (5' RRRARR 3') and R/K-G mutations blocked nucleolar localization. However, ΔNLS phase-separated with equal propensity to wild-type p26 demonstrating that phase separation alone is insufficient for p26 nucleolar partitioning. Interestingly, nucleolar retention of D/E-G p26

granules was >5-fold higher compared to wild-type p26 and was likely the result of increased protein net charge [51].

p26 must interact with Fib2 in phase-separated nucleoli to support systemic virus trafficking [36], but the role of phase separation in this interaction was previously unknown. Using *in vitro* assays with pre-formed Fib2 droplets, we demonstrated that the wild-type IDR, but not the R/K-G mutant ~~could~~ partition in Fib2 droplets. These observations suggest that p26 phase separation is required for systemic movement since p26 likely encounters pre-formed Fib2 droplets when first entering the nucleolus during infection. Indeed, R/K-G p26 failed to support systemic movement of a TMV vector but it remains unclear whether the block in systemic movement was due to R/K-G's inability to phase separate, enter the nucleolus, or a combination of both. Surprisingly, D/E-G p26 also failed move a TMV vector which could be attributed to the drastic increase in nucleolar retention of D/E-G p26. In summary, our findings demonstrate that charged amino acids play critical roles in p26 phase separation, nucleolar partitioning, and systemic virus movement.

Stress granules can support or restrict RNA virus replication and are assembled by the self-association and phase separation of G3BP [62, 63]. Seven *A. thaliana* G3BP-like candidates have been identified [73] and share an N-terminal NTF2 domain that is required for phase separation of mammalian G3BP1 [63]. In this study, the previously characterized AtG3BP-2 (AT5G43960) [61] was used to determine whether p26 could partition in G3BP stress granules. After heat shock, p26 readily partitioned inside G3BP SGs and both p26 and G3BP co-localized during virus infection. G3BP expression was upregulated during PEMV2 infection suggesting that G3BP could be expressed as part of a concerted host response to infection. PEMV2 infection was severely restricted by the over-expression of G3BP but was partially restored during expression of ΔNTF2-G3BP, demonstrating that phase separation of G3BP enhances antiviral activity towards PEMV2.

Since PEMV2 accumulation was not fully restored during ΔNTF2-G3BP expression, G3BP retains measurable antiviral activity in the dilute state. Human G3BP1 has been shown to bind and promote the degradation of mRNAs with structured 3' untranslated regions (3' UTRs) in conjunction with upframeshift 1 (Upf1) as part of the structure-mediated RNA decay (SRD) pathway [74]. PEMV2 contains a highly structured 3' UTR [75] and like many RNA viruses is inhibited by Upf1 [76, 77]. Therefore, G3BP over-expression could enhance SRD targeting of PEMV2 RNAs. It remains unknown whether p26 partitioning into G3BP SGs is beneficial or detrimental for PEMV2 replication. However, p26 disrupts the Upf1-dependent nonsense-mediated decay (NMD) pathway [43] and Upf1 is known to partition in G3BP1 SGs [78]. Partitioning of p26 into G3BP SGs has the potential to interfere with Upf1- or G3BP-dependent RNA decay pathways.

In summary, our findings demonstrate that a plant virus movement protein phase separates and partitions inside cellular phase separations, namely the nucleolus and SGs. Since nucleolar partitioning is required for virus trafficking and G3BP SG formation severely restricts PEMV2 replication, our findings highlight both beneficial and detrimental virus-host interactions mediated by phase separation.

ACKNOWLEDGEMENTS

We would like to thank Dr. Björn Krenz (Leibniz Institut DSMZ, Brunswick, Germany) for the generous gift of the G3BP:RFP construct. We would also like to thank Dr. Jonathan Dinman and Dr. Anne Simon (University of Maryland) for their thoughtful insight. We would also like to thank Dr. Anne Simon for critically reading this manuscript.

AUTHOR CONTRIBUTIONS

Conceptualization, J.P.M; Methodology, S.L.B. and J.P.M; Investigation, S.L.B., D.J.G, and J.P.M; Writing – Original Draft, J.P.M.; Writing – Review & Editing, S.L.B., D.J.G, and J.P.M; Supervision, J.P.M.

COMPETING INTERESTS

The authors declare no competing interests.

MATERIALS & METHODS

Construction of binary plant expression vectors. The pBIN61S binary vector was used to express proteins of interest from the constitutive *Cauliflower mosaic virus* (CaMV) 35S promoter. p26:GFP, R/K-G, D/E-G, and ΔNLS GFP-fusions were PCR-amplified from synthetic double-stranded DNA fragments (Integrated DNA Technologies) and cloned into pBIN61S using the *BamH*I and *Sal*I restriction sites. R/K-G and D/E-G p26:GFP fusions contain glycine substitutions for all basic or acidic p26 residues, respectively. pBIN61S-GFP has been previously described [79]. G3BP:RFP was a generous gift from Dr. Björn Krenz and has been previously described [61]. To construct ΔNTF2-G3BP:RFP, G3BP-RFP was PCR amplified with amino acids 2-125 of G3BP omitted. PCR amplification introduced forward *BamH*I and reverse *Sal*I restriction sites for cloning into pBIN61S. All DNA constructs used in this study were sequenced for accuracy.

Agroinfiltration and plant growth. All plant expression constructs used in this study were electroporated into *Agrobacterium tumerfaciens* (C58C1 strain). Liquid cultures were passaged in media containing the appropriate antibiotics and 20 µM acetosyringone 1 day prior to infiltration. Overnight cultures were pelleted and resuspended in 10 mM $MgCl_2$, 10 mM MES-K [pH 5.6], and 100 µM acetosyringone. All agroinfiltrations contained the p14 RNA silencing

suppressor from *Pothos latent virus* [80] at a final OD_{600} of 0.2. Typically, the 3rd-5th leaves from young *N. benthamiana* plants were infiltrated with a 1 mL syringe. Visualization of nuclei in agroinfiltrated leaves was achieved by infiltrating a solution of 5 µg/mL DAPI (4′,6-diamidino-2-phenylindole) into leaves 45 minutes prior to imaging. *N. benthamiana* plants were grown in a humidity-controlled chamber at 24°C, 65% humidity, and 12-hour day/night schedule (200 µmol $m^{-2}s^{-1}$).

Fluorescence recovery after photobleaching (FRAP). pBIN61S containing p26:GFP was agroinfiltrated into *N. benthamiana* using an OD_{600} = 0.4. GFP fluorescence was visible after 2 days and leaves expressing p26:GFP were wet-mounted and imaged using a Zeiss LSM 510 Meta confocal microscope with a 20X objective and Zen 2009 software. FRAP was performed by photobleaching a ~2 µm diameter region with 100% laser power (488 nm) with subsequent fluorescence recovery measured at 5 s intervals. Background regions and unbleached reference condensates were recorded as controls. Data analysis was performed as previously described [81]. Briefly, background intensity was subtracted, intensities were normalized to set the first post-bleach value to zero and presented as a fraction of the pre-bleach fluorescence intensity.

Construction of bacterial expression vectors. For C-terminal GFP-fusion recombinant protein production in *E. coli*, pRSET his-eGFP [82] was used as a backbone and was a gift from Jeanne Stachowiak (Addgene plasmid # 113551). ~~All recombinant proteins purified in this study contained N-terminal histidine-tags for affinity chromatography~~. The wild-type p26 IDR (amino acids 1-132) or p26 C-terminus (amino acids 133-226) were PCR amplified from a full-length PEMV2 infectious clone. Note: the last 10 amino acids of p26 were omitted from the C-term construct to circumvent proteolysis encountered during bacterial expression (not shown). Mutant IDRs containing R/K-G, D/E-G, or ΔNLS mutations were synthesized (Integrated DNA Technologies) as double stranded DNA fragments and were used in restriction digests and ligation reactions using T4 DNA Ligase (New England Biolabs). R/K-G and D/E-G mutants

contain glycine substitutions for all basic or acidic residues, respectively. ΔNLS is missing the sequence 5'-RRRARR-3' (amino acids 100-105) within the IDR. Wild-type IDR, was cloned into the *BamH*I restriction site of pRSET his-eGFP and sequenced for directionality and accuracy. C-term, R/K-G, D/E-G, and ΔNLS were cloned into pRSET his-eGFP using both the *Nhe*I and *BamH*I restriction sites and sequenced for accuracy.

Fibrillarin (Fib2) was first PCR amplified from cDNA synthesized from *Arabidopsis thaliana* seedling total RNA using primers Forward 5'-GCAGCAGCTAGCATGAGACCTCCTCTAACTGGAAGTGG-3' and Reverse 5'-CTGCTGCGGATCCAGCAGCAGTAGCAGCCTTTGGCTTC-3' where the underlined sequences denote the *Nhe*I and *BamH*I restriction sites used to clone the PCR fragment into pRSET-his-mCherry [83], a gift from Jeanne Stachowiak (Addgene plasmid # 113552). The resulting construct is full-length Fib2 with a C-terminal mCherry fusion (Fib2$_{FL}$). The Fib2 GAR domain was PCR amplified from Fib2$_{FL}$, digested, and ligated into the *Nhe*I and *BamH*I restriction sites of pRSET-his-mCherry to generate Fib2$_{GAR}$. Both constructs contain N-terminal histidine tags for affinity purification.

Protein expression and purification. Histidine-tagged recombinant proteins were expressed in BL21(DE3) *E. coli* (New England Biolabs) using autoinduction Luria-Bertani (LB) broth and purified using HisPur™ cobalt spin columns (Thermo Scientific). Proteins were purified under denaturing conditions according to the manufacturer's protocol using 8 M urea. All equilibration, wash, and elution buffers contained 1 M NaCl to suppress phase separation. Following elution of recombinant proteins from the cobalt resin, proteins were re-folded through dialysis in buffer containing 10 mM Tris-HCl (pH 7.0), 300 mM NaCl, 1 mM EDTA, 1 mM dithiothreitol, and 10% glycerol as previously used for the related pORF3 from *Groundnut rosette virus* [42]. Urea was removed in a stepwise fashion by using dialysis buffers containing 4 M Urea, 1 M Urea, or no Urea. Proteins were concentrated using centrifugal filters and concentrations were measured using a Bicinchoninic acid (BCA) protein assay (Millipore

Sigma). Protein integrity and purity was assessed by SDS-PAGE. If necessary, hydrophobic interaction chromatography (Methyl HIC resin) was used to further purify and concentrate GFP-fusion samples according to the manufacturers protocol (Bio-Rad).

In vitro phase separation assays. For *in vitro* assays, recombinant proteins were used at a final concentration of 8 µM unless otherwise noted ~~in the figures or text~~. Phase separation assays consisted of the following mixture: 8 µM protein, 10 mM Tris-HCl (pH 7.5), 1 mM DTT, 100 mM NaCl, and 10% PEG-8000 to induce phase separation. Phase separation occurred rapidly and samples were directly loaded onto glass slides for confocal microscopy using a Zeiss LSM 510 Meta confocal microscope with a 20x objective and appropriate filters. High-salt conditions included NaCl at a final concentration of 1 M and “no treatment” did not include PEG-8000. Phase separation assays were performed at least twice across two protein preparations. Turbidity assays comparing IDR-GFP with controls or IDR mutants were performed with either 8 µM or 24 µM protein under standard assay conditions. 100 µL reactions were placed at room temperature for 15 minutes prior to OD_{600} measurements using a 96-well plate reader. ImageJ was used to measure droplet size (condensate area) from thresholded images (20x objective) using the built-in “analyze particles” tool.

RNA sorting assays. Cy5-labelled PEMV2 or TCV RNA was synthesized by T7 run-off transcription using *Sma*I-linearized full-length infectious clones. Cy5-labelled *Renilla* luciferase (RLuc) RNAs were synthesized from PCR products containing a T7 promoter, RLuc ORF, and a 13-nt 3’ untranslated region. Cy5-UTP (APExBIO) was added to *in vitro* transcription reactions according to the HiScribe T7 Quick High Yield RNA Synthesis Kit protocol (New England Biolabs). RNAs were included in phase separation assays at a final concentration of 16 nM (1:500 RNA:protein ratio). Mander’s overlap coefficients (MOC) were used to measure the fraction of IDR-GFP that was positive for Cy5-labelled RNA from 20x fields of view using the ImageJ plugin EzColocalization [84].

Construction and agroinfiltration of Tobacco mosaic virus (TMV) vectors. The TMV vector pJL-TRBO has been previously described [58] and was a gift from John Lindbo (Addgene plasmid # 80082). The TMV vector containing p26:GFP has also been previously described [43]. R/K-G and D/E-G GFP-fusions were PCR amplified from synthetic DNA fragments with introduced *Pac*I and *Not*I restriction sites for digestion and ligation into the corresponding pJL-TRBO sites. R/K-G and D/E-G constructs contain full-length p26 with glycine substitutions for all basic or acidic residues, respectively. Both R/K-G and D/E-G contain a C-terminal GFP tag. Constructs were sanger sequenced for accuracy.

TMV movement assay and RT-PCR. pJL-TRBO derived TMV vectors expressing GFP or p26-GFP fusions were agroinfiltrated (OD_{600} = 0.4) into young *N. benthamiana* plants (3rd-4th true leaf stage). GFP fluorescence in local and systemic leaves was monitored daily. At 4 dpi, robust local infections were evident, and leaves were imaged (488 nm) prior to grinding in liquid nitrogen. Total protein was extracted by resuspending leaf tissue in 1X PBS supplemented with 3% β-mercaptoethanol and protease inhibitor cocktail (Thermo Scientific). Samples were mixed with 6X Laemmli SDS buffer, boiled, and separated by SDS-PAGE. A semi-dry transfer method was used to transfer proteins to nitrocellulose for western blotting using anti-GFP antibodies (Life technologies) at a 1:5000 dilution. Anti-rabbit IgG conjugated with horseradish peroxidase was used as a secondary antibody again with a 1:5000 dilution. Blots were visualized using the Pierce enhanced chemiluminescence kit (Thermo Scientific). Systemic leaves were harvested at 14 dpi for total RNA extraction using Trizol. 100 ng total RNA was digested with RQ1 DNase (Promega) and served as template for reverse transcription using iScript supermix (Bio-Rad). No reverse transcriptase controls (-RT) were Included for all sample and primer sets. 1 µL cDNA was used as template for 25 cycles of PCR using GoTaq polymerase (Promega) targeting the TMV replicase using forward primer 5’ CCGCGAATCTTATGTGGAAT 3’ and reverse primer 5’ TCCTCCAAGTGTTCCCAATC 3’. *N. benthamiana* actin was amplified by 31

cycles of PCR as a loading control with forward primer 5' TCCTGATGGGCAAGTGATTAC 3' and reverse primer 5' TTGTATGTGGTCTCGTGGATTC 3'.

G3BP expression and visualization. G3BP expression constructs were agroinfiltrated into *N. benthamiana* plants at an OD_{600} = 0.4 alongside p14. Heat shock of G3BP-expressing plants was performed by incubating plants at 37°C for 45 minutes prior to imaging. To determine whether p26:GFP partitions in G3BP SGs, pBIN-p26:GFP was co-infiltrated with G3BP:RFP 2-3 days prior to heat shock. To visualize G3BP:RFP alongside p26:GFP during virus infection, young *N. benthamiana* plants (3-4 leaf stage) were first infiltrated with TMV:p26:GFP. After strong p26:GFP signal was observed in the systemic leaves (typically ~2-3 weeks), G3BP:RFP was agroinfiltrated and imaged at 5 dpi using a Zeiss LSM 510 Meta confocal microscope with a 20x objective. The full-length PEMV2 expression construct has been previously described [76] and was agroinfiltrated alongside full-length G3BP or ΔNTF2-G3BP at a final OD_{600} of 0.2. Using the same protocol as above, western blotting with anti-RFP antibodies (Thermo Scientific, 1:5000 dilution) was performed to measure full-length G3BP or ΔNTF2 expression levels following agroinfiltration.

RT-qPCR. Agroinfiltrated "spots" were cut from leaves and stored at -80ºC. Samples were ground in liquid nitrogen and total RNA was extracted using the Quick-RNA Plant Kit (Zymo Research). An on-column DNase I step was added using RQ1 DNase (Promega). Total RNAs were used as templates for SYBR green-based one-step reverse-transcriptase quantitative PCR (RT-qPCR) using the NEB Luna One-Step RT-qPCR kit (New England Biolabs). All primers were validated by standard curve analysis and had PCR efficiencies ranging from 90-110%. Native *N. benthamiana* G3BP (Transcript ID: Niben101Scf03456g00002.1) was targeted using primers Forward 5' TAGGGGAAGCAATCCAGATG 3' and Reverse 5' TCCTTATCGATCCCAACAGC 3'. PEMV2 genomic RNA was targeted by forward primer 5' TTGCAAGGTTCTAGGCATCC 3' and reverse primer 5' CAACGATCGAAAAAGACGATG 3'. Gene expression was normalized to the internal

control transcripts from the agroinfiltrated p14 RNA silencing suppressor using forward primer 5' TCCCAAACAGGGGTTTTATG 3' and reverse primer 5' GGTAATTGGGAACCCTCGAT 3'. Expression analyses were performed by the ΔΔCq method using Bio-Rad CFX Maestro software. Target fidelity was monitored by melt curve analyses and no reverse transcriptase controls.

REFERENCES

1. Inoue T, Tsai B. How viruses use the endoplasmic reticulum for entry, replication, and assembly. Cold Spring Harb Perspect Biol. 2013;5(1):a013250-a.

2. Anand SK, Tikoo SK. Viruses as modulators of mitochondrial functions. Adv Virol. 2013;2013:738794-.

3. Walker EJ, Ghildyal R. Editorial: Viral Interactions with the Nucleus. Front Microbiol. 2017;8:951-.

4. Miller S, Krijnse-Locker J. Modification of intracellular membrane structures for virus replication. Nat Rev Microbiol. 2008;6(5):363-74.

5. Dolgin E. What lava lamps and vinaigrette can teach us about cell biology. Nature. 2018;555(7696):300-2.

6. Tang L. Liquid phase separation. Nat Methods. 2019;16(1):18-.

7. Elbaum-Garfinkle S. Matter over mind: Liquid phase separation and neurodegeneration. J Biol Chem. 2019;294(18):7160-8.

8. Drino A, Schaefer MR. RNAs, Phase Separation, and Membrane-Less Organelles: Are Post-Transcriptional Modifications Modulating Organelle Dynamics? BioEssays. 2018;40(12):1800085.

9. Zhang H, Elbaum-Garfinkle S, Langdon EM, Taylor N, Occhipinti P, Bridges AA, et al. RNA Controls PolyQ Protein Phase Transitions. Mol Cell. 2015;60(2):220-30.

10. Vernon RM, Chong PA, Tsang B, Kim TH, Bah A, Farber P, et al. Pi-Pi contacts are an overlooked protein feature relevant to phase separation. Elife. 2018;7:e31486.

11. Murthy AC, Dignon GL, Kan Y, Zerze GH, Parekh SH, Mittal J, et al. Molecular interactions underlying liquid-liquid phase separation of the FUS low-complexity domain. Nat Struct Mol Biol. 2019;26(7):637-48.

12. Boeynaems S, Alberti S, Fawzi NL, Mittag T, Polymenidou M, Rousseau F, et al. Protein Phase Separation: A New Phase in Cell Biology. Trends Cell Biol. 2018;28(6):420-35.

13. Shorter J. Phase separation of RNA-binding proteins in physiology and disease: An introduction to the JBC Reviews thematic series. J Biol Chem. 2019;294(18):7113-4.

14. Riback JA, Katanski CD, Kear-Scott JL, Pilipenko EV, Rojek AE, Sosnick TR, et al. Stress-Triggered Phase Separation Is an Adaptive, Evolutionarily Tuned Response. Cell. 2017;168(6):1028-40.e19.

15. Matsuki H, Takahashi M, Higuchi M, Makokha GN, Oie M, Fujii M. Both G3BP1 and G3BP2 contribute to stress granule formation. Genes Cells. 2013;18(2):135-46.

16. Jain S, Wheeler JR, Walters RW, Agrawal A, Barsic A, Parker R. ATPase-Modulated Stress Granules Contain a Diverse Proteome and Substructure. Cell. 2016;164(3):487-98.

17. Wheeler JR, Matheny T, Jain S, Abrisch R, Parker R. Distinct stages in stress granule assembly and disassembly. Elife. 2016;5:e18413.

18. Cristea IM, Rozjabek H, Molloy KR, Karki S, White LL, Rice CM, et al. Host factors associated with the Sindbis virus RNA-dependent RNA polymerase: role for G3BP1 and G3BP2 in virus replication. J Virol. 2010;84(13):6720-32.

19. Götte B, Panas MD, Hellström K, Liu L, Samreen B, Larsson O, et al. Separate domains of G3BP promote efficient clustering of alphavirus replication complexes and recruitment of the translation initiation machinery. PLoS Pathog. 2019;15(6):e1007842.

20. Hosmillo M, Lu J, McAllaster MR, Eaglesham JB, Wang X, Emmott E, et al. Noroviruses subvert the core stress granule component G3BP1 to promote viral VPg-dependent translation. Elife. 2019;8.

21. Yang W, Ru Y, Ren J, Bai J, Wei J, Fu S, et al. G3BP1 inhibits RNA virus replication by positively regulating RIG-I-mediated cellular antiviral response. Cell Death Dis. 2019;10(12):946.

22. Pandey K, Zhong S, Diel DG, Hou Y, Wang Q, Nelson E, et al. GTPase-activating protein-binding protein 1 (G3BP1) plays an antiviral role against porcine epidemic diarrhea virus. Vet Microbiol. 2019;236:108392.

23. Reineke LC, Kedersha N, Langereis MA, van Kuppeveld FJ, Lloyd RE. Stress granules regulate double-stranded RNA-dependent protein kinase activation through a complex containing G3BP1 and Caprin1. mBio. 2015;6(2):e02486.

24. Nikolic J, Le Bars R, Lama Z, Scrima N, Lagaudrière-Gesbert C, Gaudin Y, et al. Negri bodies are viral factories with properties of liquid organelles. Nat Commun. 2017;8(1):58.

25. Zhou Y, Su JM, Samuel CE, Ma D. Measles Virus Forms Inclusion Bodies with Properties of Liquid Organelles. J Virol. 2019;93(21).

26. Heinrich BS, Maliga Z, Stein DA, Hyman AA, Whelan SPJ. Phase Transitions Drive the Formation of Vesicular Stomatitis Virus Replication Compartments. mBio. 2018;9(5):e02290-17.

27. Cascarina SM, Ross ED. A proposed role for the SARS-CoV-2 nucleocapsid protein in the formation and regulation of biomolecular condensates. FASEB J. 2020;34(8) :9832-9842.

28. Iserman C, Roden CA, Boerneke MA, Sealfon RSG, McLaughlin GA, Jungreis I, et al. Genomic RNA Elements Drive Phase Separation of the SARS-CoV-2 Nucleocapsid. Mol Cell. 2020;80(6):1078-91.e6.

29. Perdikari TM, Murthy AC, Ryan VH, Watters S, Naik MT, Fawzi NL. SARS-CoV-2 nucleocapsid protein phase-separates with RNA and with human hnRNPs. EMBO J. 2020;39(24):e106478.

30. Carlson CR, Asfaha JB, Ghent CM, Howard CJ, Hartooni N, Safari M, et al. Phosphoregulation of Phase Separation by the SARS-CoV-2 N Protein Suggests a Biophysical Basis for its Dual Functions. Mol Cell. 2020;80(6):1092-103.e4.

31. Li J, Guo M, Tian X, Wang X, Yang X, Wu P, et al. Virus-Host Interactome and Proteomic Survey Reveal Potential Virulence Factors Influencing SARS-CoV-2 Pathogenesis. Med. 2021;15;2(1):99-112.e7

32. Nabeel-Shah S, Lee H, Ahmed N, Marcon E, Farhangmehr S, Pu S, et al. SARS-CoV-2 Nucleocapsid protein attenuates stress granule formation and alters gene expression via direct interaction with host mRNAs. bioRxiv. 2020:2020.10.23.342113.

33. Canetta E, Kim SH, Kalinina NO, Shaw J, Adya AK, Gillespie T, et al. A plant virus movement protein forms ringlike complexes with the major nucleolar protein, fibrillarin, in vitro. J Mol Biol. 2008;376(4):932-7.

34. Kim SH, MacFarlane S, Kalinina NO, Rakitina DV, Ryabov EV, Gillespie T, et al. Interaction of a plant virus-encoded protein with the major nucleolar protein fibrillarin is required for systemic virus infection. Proc Natl Acad Sci U S A. 2007;104(26):11115.

35. Kim SH, Ryabov EV, Kalinina NO, Rakitina DV, Gillespie T, MacFarlane S, et al. Cajal bodies and the nucleolus are required for a plant virus systemic infection. EMBO J. 2007;26(8):2169-79.

36. Kim SH, Macfarlane S, Kalinina NO, Rakitina DV, Ryabov EV, Gillespie T, et al. Interaction of a plant virus-encoded protein with the major nucleolar protein fibrillarin is required for systemic virus infection. Proc Natl Acad Sci U S A. 2007;104(26):11115-20.

37. Ryabov EV, Oparka KJ, Santa Cruz S, Robinson DJ, Taliansky ME. Intracellular location of two groundnut rosette umbravirus proteins delivered by PVX and TMV vectors. Virology. 1998;242(2):303-13.

38. Kalinina NO, Makarova S, Makhotenko A, Love AJ, Taliansky M. The Multiple Functions of the Nucleolus in Plant Development, Disease and Stress Responses. Front Plant Sci. 2018;9(132).

39. Haupt S, Stroganova T, Ryabov E, Kim SH, Fraser G, Duncan G, et al. Nucleolar localization of potato leafroll virus capsid proteins. J Gen Virol. 2005;86(Pt 10):2891-6.

40. Chang C-H, Hsu F-C, Lee S-C, Lo Y-S, Wang J-D, Shaw J, et al. The Nucleolar Fibrillarin Protein Is Required for Helper Virus-Independent Long-Distance Trafficking of a Subviral Satellite RNA in Plants. Plant Cell. 2016;28(10):2586-602.

41. Feric M, Vaidya N, Harmon TS, Mitrea DM, Zhu L, Richardson TM, et al. Coexisting Liquid Phases Underlie Nucleolar Subcompartments. Cell. 2016;165(7):1686-97.

42. Taliansky M, Roberts IM, Kalinina N, Ryabov EV, Raj SK, Robinson DJ, et al. An umbraviral protein, involved in long-distance RNA movement, binds viral RNA and forms unique, protective ribonucleoprotein complexes. J Virol. 2003;77(5):3031-40.

43. May JP, Johnson PZ, Ilyas M, Gao F, Simon AE. The Multifunctional Long-Distance Movement Protein of Pea Enation Mosaic Virus 2 Protects Viral and Host Transcripts from Nonsense-Mediated Decay. mBio. 2020;11(2):e00204-20.

44. Ishikawa-Ankerhold H, Ankerhold, R. and Drummen, G. . Fluorescence Recovery After Photobleaching (FRAP). In eLS, John Wiley & Sons, Ltd (Ed)2014.

45. Dosztányi Z. Prediction of protein disorder based on IUPred. Protein Sci. 2018;27(1):331-40.

46. Bolognesi B, Lorenzo Gotor N, Dhar R, Cirillo D, Baldrighi M, Tartaglia GG, et al. A Concentration-Dependent Liquid Phase Separation Can Cause Toxicity upon Increased Protein Expression. Cell Rep. 2016;16(1):222-31.

47. Kroschwald S, Maharana S, Alberti S. Hexanediol: a chemical probe to investigate the material properties of membrane-less compartments. Matters. 2017 May 22; http://dx.doi.org/10.19185/matters.201702000010.

48. Luo H, Lee N, Wang X, Li Y, Schmelzer A, Hunter AK, et al. Liquid-liquid phase separation causes high turbidity and pressure during low pH elution process in Protein A chromatography. J Chromatogr A. 2017;1488:57-67.

49. Ryabov EV, Kim SH, Taliansky M. Identification of a nuclear localization signal and nuclear export signal of the umbraviral long-distance RNA movement protein. J Gen Virol. 2004;85(Pt 5):1329-33.

50. Savada RP, Bonham-Smith PC. Charge versus sequence for nuclear/nucleolar localization of plant ribosomal proteins. Plant Mol Biol. 2013;81(4-5):477-93.

51. Musinova YR, Kananykhina EY, Potashnikova DM, Lisitsyna OM, Sheval EV. A charge-dependent mechanism is responsible for the dynamic accumulation of proteins inside nucleoli. Biochim Biophys Acta Mol Cell Res. 2015;1853(1):101-10.

52. Frottin F, Schueder F, Tiwary S, Gupta R, Körner R, Schlichthaerle T, et al. The nucleolus functions as a phase-separated protein quality control compartment. Science. 2019; 26;365(6451):342-347.

53. Rakitina DV, Taliansky M, Brown JWS, Kalinina NO. Two RNA-binding sites in plant fibrillarin provide interactions with various RNA substrates. Nucleic Acids Res. 2011;39(20):8869-80.

54. Yao RW, Xu G, Wang Y, Shan L, Luan PF, Wang Y, et al. Nascent Pre-rRNA Sorting via Phase Separation Drives the Assembly of Dense Fibrillar Components in the Human Nucleolus. Mol Cell. 2019;76(5):767-83.e11.

55. Berry J, Weber SC, Vaidya N, Haataja M, Brangwynne CP. RNA transcription modulates phase transition-driven nuclear body assembly. Proc Natl Acad Sci U S A. 2015;112(38):E5237.

56. Banani SF, Rice AM, Peeples WB, Lin Y, Jain S, Parker R, et al. Compositional Control of Phase-Separated Cellular Bodies. Cell. 2016;166(3):651-63.

57. Ditlev JA, Case LB, Rosen MK. Who's In and Who's Out—Compositional Control of Biomolecular Condensates. J Mol Biol. 2018;430(23):4666-84.

58. Lindbo JA. TRBO: A High-Efficiency Tobacco Mosaic Virus RNA-Based Overexpression Vector. Plant Physiol. 2007;145(4):1232.

59. Ryabov EV, Robinson DJ, Taliansky ME. A plant virus-encoded protein facilitates long-distance movement of heterologous viral RNA. Proc Natl Acad Sci U S A. 1999;96(4):1212-7.

60. Ryabov EV, Robinson DJ, Taliansky M. Umbravirus-encoded proteins both stabilize heterologous viral RNA and mediate its systemic movement in some plant species. Virology. 2001;288(2):391-400.

61. Krapp S, Greiner E, Amin B, Sonnewald U, Krenz B. The stress granule component G3BP is a novel interaction partner for the nuclear shuttle proteins of the nanovirus pea necrotic yellow dwarf virus and geminivirus abutilon mosaic virus. Virus Res. 2017;227:6-14.

62. Tourrière H, Chebli K, Zekri L, Courselaud B, Blanchard JM, Bertrand E, et al. The RasGAP-associated endoribonuclease G3BP assembles stress granules. J Cell Biol. 2003;160(6):823-31.

63. Guillén-Boixet J, Kopach A, Holehouse AS, Wittmann S, Jahnel M, Schlüßler R, et al. RNA-Induced Conformational Switching and Clustering of G3BP Drive Stress Granule Assembly by Condensation. Cell. 2020;181(2):346-61.e17.

64. Nevers Q, Albertini AA, Lagaudrière-Gesbert C, Gaudin Y. Negri bodies and other virus membrane-less replication compartments. Biochim Biophys Acta Mol Cell Res. 2020;1867(12):118831-.

65. Lahaye X, Vidy A, Pomier C, Obiang L, Harper F, Gaudin Y, et al. Functional characterization of Negri bodies (NBs) in rabies virus-infected cells: Evidence that NBs are sites of viral transcription and replication. J Virol. 2009;83(16):7948-58.

66. Belov GA, van Kuppeveld FJ. (+)RNA viruses rewire cellular pathways to build replication organelles. Curr Opin Virol. 2012;2(6):740-7.

67. Nagy PD, Strating JR, van Kuppeveld FJ. Building Viral Replication Organelles: Close Encounters of the Membrane Types. PLoS Pathog. 2016;12(10):e1005912.

68. Alers-Velazquez R, Jacques S, Muller C, Boldt J, Schoelz J, Leisner S. Cauliflower mosaic virus P6 inclusion body formation: A dynamic and intricate process. Virology. 2021;553:9-22.

69. Li Q, Liu N, Liu Q, Zheng X, Lu L, Gao W, et al. DEAD-box helicases modulate dicing body formation in Arabidopsis. Sci Adv. 2021; 28;7(18):eabc6266.

70. Yang Z, Li Y. Dissection of RNAi-based antiviral immunity in plants. Curr Opin Virol. 2018;32:88-99.

71. Boeynaems S, Bogaert E, Kovacs D, Konijnenberg A, Timmerman E, Volkov A, et al. Phase Separation of C9orf72 Dipeptide Repeats Perturbs Stress Granule Dynamics. Mol Cell. 2017;65(6):1044-55.e5.

72. Brangwynne Clifford P, Tompa P, Pappu Rohit V. Polymer physics of intracellular phase transitions. Nat Phys. 2015;11(11):899-904.

73. Reuper H, Amari K, Krenz B. Analyzing the G3BP-like gene family of Arabidopsis thaliana in early turnip mosaic virus infection. Sci Rep. 2021;11(1):2187.

74. Fischer JW, Busa VF, Shao Y, Leung AKL. Structure-Mediated RNA Decay by UPF1 and G3BP1. Mol Cell. 2020;78(1):70-84.e6.

75. Simon AE, Miller WA. 3' cap-independent translation enhancers of plant viruses. Annu Rev Microbiol. 2013;67:21-42.

76. May JP, Yuan X, Sawicki E, Simon AE. RNA virus evasion of nonsense-mediated decay. PLoS Pathog. 2018;14(11):e1007459.

77. May JP, Simon AE. Targeting of viral RNAs by Upf1-mediated RNA decay pathways. Curr Opin Virol. 2020;47:1-8.

78. Brown JAL, Roberts TL, Richards R, Woods R, Birrell G, Lim YC, et al. A novel role for hSMG-1 in stress granule formation. Mol Cell Biol. 2011;31(22):4417-29.

79. Kertesz S, Kerenyi Z, Merai Z, Bartos I, Palfy T, Barta E, et al. Both introns and long 3'-UTRs operate as cis-acting elements to trigger nonsense-mediated decay in plants. Nucleic Acids Res. 2006;34(21):6147-57.

80. Merai Z, Kerenyi Z, Molnar A, Barta E, Valoczi A, Bisztray G, et al. Aureusvirus P14 is an efficient RNA silencing suppressor that binds double-stranded RNAs without size specificity. J Virol. 2005;79(11):7217-26.

81. Boeynaems S, De Decker M, Tompa P, Van Den Bosch L. Arginine-rich Peptides Can Actively Mediate Liquid-liquid Phase Separation. Bio Protoc. 2017;7(17):e2525.

82. Busch DJ, Houser JR, Hayden CC, Sherman MB, Lafer EM, Stachowiak JC. Intrinsically disordered proteins drive membrane curvature. Nat Commun. 2015;6:7875.

83. DeGroot ACM, Busch DJ, Hayden CC, Mihelic SA, Alpar AT, Behar M, et al. Entropic Control of Receptor Recycling Using Engineered Ligands. Biophys J. 2018;114(6):1377-88.

84. Stauffer W, Sheng H, Lim HN. EzColocalization: An ImageJ plugin for visualizing and measuring colocalization in cells and organisms. Sci Rep. 2018;8(1):15764.

FIGURE LEGENDS

Fig. 1. **p26 forms poorly dynamic condensates *in vivo***. (A) PEMV2 is a small positive-sense RNA plant virus that encodes 4 genes, including the p26 long-distance movement protein. Free GFP and p26 C-terminally fused with GFP (p26:GFP) were expressed from binary expression plasmids under the constitutive CaMV 35S promoter (B) Following agroinfiltration of *N. benthamiana,* confocal microscopy showed diffuse cytoplasmic and nuclear expression of free GFP whereas p26:GFP formed large cytoplasmic bodies. Note that the majority of plant mesophyll cells is taken up by a single large vacuole. Differential interference contrast (DIC) microscopy was used for p26:GFP samples to visualize cell borders. Bar scale: 20 µm. (C) FRAP analysis of p26:GFP was performed by photobleaching cytoplasmic condensates and monitoring fluorescence recovery at 5 s intervals. A representative p26:GFP condensate is shown before photobleaching, immediately following photobleaching (5 s), and at 120 s. Bar scale 5 µm. Average FRAP intensity is shown from seven FRAP experiments and shaded area represents 95% confidence interval.

Fig. 2. **p26 is intrinsically disordered and phase separates through electrostatic interactions**. (A) (Top) The IUPRED algorithm [45] predicts that PEMV2 p26 contains a large intrinsically disordered region (IDR) spanning amino acids 1-132. (Bottom) The same region has the highest predicted phase separation propensity using the catGRANULE algorithm [46]. (B) The p26 IDR was fused to the N-terminus of GFP for bacterial expression and contained an N-terminal histidine tag. The p26 IDR sequence is shown with highlighted residues corresponding to basic (blue) or acidic (red) residues. The conserved nuclear localization signal (NLS) is highlighted in yellow. (C) Recombinant proteins used in this study were analyzed by SDS-PAGE to assess size and purity. Proteins were stained using Coomassie Blue. Marker (M) sizes are

shown in kilodaltons (kDa). R/K-G and D/E-G IDR mutants contain glycine substitutions for all basic or acidic IDR residues, respectively. ΔNLS is missing the nuclear localization signal 5'-RRRARR-3' (amino acids 100-105) within the IDR. Note: R/K-G ran markedly higher both *in vitro* and *in vivo* (see Fig. 6B). (D) *In vitro* phase separation assays were visualized by confocal microscopy. 8 µM protein was used for all assays and 10% PEG-8000 was added as a crowding agent (Middle panels). One molar NaCl was added to disrupt electrostatic interactions (Right panel). Bar scale: 20 µm. (E) Turbidity assays (OD_{600}) using either 8 µM or 24 µM protein were performed for all constructs. Only IDR-ΔNLS turbidity was not significantly reduced compared to IDR-GFP. **** P<0.0001 by two-way ANOVA with Dunnett's multiple comparisons test vs. IDR-GFP. (F) Phase diagram for IDR-GFP gives an apparent C_{sat} = 2 µM and sensitivity to high NaCl concentrations. Results are representative of two independent experiments. (G) Mean condensate sizes for all mutants (excluding R/K-G) were plotted by cumulative distribution frequency. Particle sizes were measured from three representative 20x fields using ImageJ. *P* values represent results from two-tailed Mann-Whitney tests compared to IDR-GFP. ns: not significant.

Fig. 3. **Charged residues govern p26 nucleolar partitioning**. (A) p26:GFP fusions were expressed from the CaMV 35S promoter in *N. benthamiana* leaves following agroinfiltration. Prior to imaging, leaves were infiltrated with 5 µg/mL DAPI to stain nuclei. 20x and 63x fields are shown. Arrows denote p26 partitioned inside Nuclear Bodies (NBs). Bar scale: Top 20 µm; Bottom 10 µm. (B) Nuclear granules were manually counted from six 20x fields. Total granule counts were calculated using the ImageJ "analyze particles" tool. Error bars denote standard deviations. ****P<0.0001 unpaired t test.

Fig 4. p26 phase separation is required for partitioning in Fib2 droplets. (A) Fib2 contains an N-terminal glycine- and arginine-rich (GAR) domain that is intrinsically disordered. (B) Either the Fib2 GAR domain ($Fib2_{GAR}$) or full-length Fib2 ($Fib2_{FL}$) were fused to mCherry and purified from *E. coli* and analyzed by SDS-PAGE. Molecular weight (kDa) marker is shown. (C) mCherry, $Fib2_{GAR}$, and $Fib2_{FL}$ were examined by confocal microscopy after inducing phase separation with 10% PEG-8000 alone or in the presence of 1 M NaCl. 8 µM protein was used for all assays. Bar scale: 20 µm. (D) $Fib2_{GAR}$ droplets were pre-formed using 24 µM protein before the addition of 4 µM IDR-GFP or R/K-G. Sorting of IDR-GFP to Fib2 droplets was observed whereas R/K-G remained in the bulk phase and failed to partition in $Fib2_{GAR}$ droplets (White arrows). Bar scale 10 µm.

Fig. 5. vRNPs required for systemic trafficking can be reconstituted *in vitro* via phase separation. (A) $Fib2_{GAR}$ and $Fib2_{FL}$ droplets were pre-formed prior to the addition of PEMV2-Cy5 RNAs at a 1:500 RNA:protein molar ratio. PEMV2 RNA was only efficiently sorted to $Fib2_{FL}$ condensates. Bar scale: 20 µm. (B) IDR-GFP droplets were pre-formed prior to the addition of PEMV2-Cy5, TCV-Cy5, or RLuc-Cy5 RNAs at a 1:500 RNA:protein molar ratio. Bar scale: 20 µm. (C) The fraction of IDR-GFP signal that was positive for Cy5-labelled RNA was determined by MOC analysis using EzColocalization [84]. ns: not significant by unpaired t test. Bars denote standard deviations. Three 20x fields were quantified for each condition. (D) IDR-GFP, $Fib2_{FL}$, and PEMV2-Cy5 RNA were mixed at a 500:500:1 molar ratio under crowding conditions. Droplets containing all components were observed. Bar scale: 10 µm. Images in all panels are representative of at least two independent experiments.

Fig. 6. Phase separation-deficient p26 mutants fail to systemically traffic a virus vector. (A) pJL-TRBO TMV vector lacks coat protein (CP) and is severely impaired in systemic trafficking. Free GFP, p26:GFP, R/K-G, and D/E-G GFP fusions were inserted into pJL-TRBO to

test whether systemic trafficking could be restored. (B) Following agroinfiltration of *N. benthamiana* leaves, TMV infections were established in local leaves. Free GFP, or GFP-fusion proteins were visualized and detected in local leaves at 4 dpi by UV exposure (Left) or western blotting (Right). Rubisco serves as a loading control. Red asterisks denote free GFP or GFP-fusion bands. (C) Localization patterns in TMV-infected leaves confirmed that neither free GFP or R/K-G form phase separated granules. Bar scale: 20 µm. Nuclear p26:GFP or D/E-G granules were counted from 5 20x fields of view and divided by the total number of granules (counted with ImageJ) to calculate a percentage (%). The fraction of D/E-G nuclear granules was significantly higher than observed for wild-type. Expression patterns did not differ between 35S-driven or TMV-expressed p26:GFP or D/E-G. 35S promoter data from Fig. 3B was included for comparison. (D) At 14 dpi, systemic leaves were imaged prior to total RNA extraction. RT-PCR was used to amplify 100-200 bp fragments targeting either the TMV replicase or actin as a control. -RT: No reverse transcriptase controls. Two pools of 3-4 leaves are shown for each construct. Results are representative of three independent experiments consisting of at least 4 plants/construct.

Fig. 7. **p26 is sorted into G3BP phase separations that restrict PEMV2 accumulation**. (A) *A. thaliana* G3BP contains an ordered NTF2 domain and RNA recognition motif (RRM) in addition to intrinsically disordered regions. (B) G3BP:RFP or ΔNTF2-G3BP:RFP were expressed from CaMV 35S promoters following agroinfiltration of *N. benthamiana* leaves. At 3 dpi, plants were either imaged directly or heat shocked for 45 minutes at 37°C. p26:GFP was co-infiltrated with G3BP:RFP and p26 partitioning in G3BP SGs was observed (White arrows). Scale bar: 20 µm. Inset shows western blot using anti-RFP antibodies to detect full-length G3BP and ΔNTF2-G3BP. Rubisco was used as a loading control (C) G3BP:RFP was agroinfiltrated into *N. benthamiana* plants systemically infected with TMV (pJL-TRBO) expressing p26:GFP.

Confocal microscopy was used to observe co-localization (White arrows) between p26 and G3BP during virus infection. Scale bar: 20 µm. (D) Native G3BP expression was measured in Mock- or PEMV2-infected *N. benthamiana* at 3 dpi by RT-qPCR. The agroinfiltrated p14 RNA silencing suppressor was used as a reference gene. Data is from three biological replicates. **P*<0.05; student's t-test. Bars denote standard error. (E) PEMV2 was agroinfiltrated alone, or alongside either G3BP or ΔNTF2-G3BP (both tagged with RFP). At 3 dpi, total protein and total RNA was extracted and used for western blot or RT-qPCR targeting PEMV2 or p14 (reference gene), respectively. Full-length G3BP and ΔNTF2 accumulated to similar levels when detected by anti-RFP antibody (top). RT-qPCR results represent 7 biological replicates from 2 independent experiments. Bars denote standard error. Brown-Forsythe and Welch ANOVA with multiple comparisons was used to determine if observed differences were significant. ** *P*<0.01.

Supplemental Fig. 1. Characterization of His-tagged and untagged IDR-GFP. (A) Coomassie-stained SDS-PAGE analysis shows expected subtle downward shift by IDR-GFP following His-tag cleavage with recombinant enterokinase (rEK). (B) Untagged IDR-GFP appeared identical to His-tagged IDR-GFP under crowding or high-salt conditions. Bar scale: 20 µm. (C) *In vitro* turbidity assay (OD_{600}) revealed untagged and tagged IDR-GFP phase separated with the same propensity. Three independent replicates are shown. (D) Particle sizes of tagged and untagged IDR-GFP droplets from three 20x fields were measured using ImageJ. ns: not significant by two-tailed Mann-Whitney test. (E) His-tagged and untagged IDR-GFP were mixed with 10% 1,6 hexanediol to assess the viscosity of droplets. The presence of a His-tag had no effect on sensitivity towards 1,6 hexanediol. (F) Droplet dynamics of His-tagged and untagged IDR-GFP were measured by FRAP. Results are from 9 FRAP experiments with representative droplets and heat map overlays shown for each construct. His-tagged IDR-GFP recovered 14% after two minutes while untagged IDR-GFP recovered 83% during the same

period. (G) RLuc-Cy5 RNAs were mixed with tagged and untagged IDR-GFP at a 1:500 RNA:protein ratio. The fraction of IDR-GFP signal that was positive for Cy5-labelled RNA was determined by Mander's Overlap Coefficient (MOC) analysis. ns: not significant by unpaired t test.

Supplemental Fig. 2. Aggregate formation by R/K-G. 24 µM protein was mixed with 10% PEG-8000 to induce phase separation in standard assay buffer. IDR-GFP and D/E-G formed uniform droplets whereas R/K-G formed non-uniform aggregates. Bar scale: 5 µm

Supplemental Fig. 3. Cation-pi and hydrophobic interactions do not influence p26 phase separation. (A) SDS-PAGE analysis of recombinant R-K and VLIMFYW-S IDR mutants. Marker weights are shown on left in kilodaltons (kDa). (B) Phase separation of R-K and VLIMFYW-S IDR mutants was compared to IDR-GFP. The R-K IDR mutation substituted lysine (K) for all arginines (R) whereas VLIMFYW-S contains serine (S) substitutions for all hydrophobic residues. R-K mutation blocks potential cation-pi interactions whereas VLIMFYW-S mutation prevents hydrophobic interactions. No differences were observed with either 10% PEG-8000 or PEG + 1 M NaCl. 8 µM protein was used for all assays. (C) Turbidity assays (OD_{600}) were performed to compare GFP alone, IDR-GFP, R-K, and VLIMFYW-S phase separation propensities. Only free GFP turbidity was significantly reduced compared to IDR-GFP. **** $P<0.0001$ by two-way ANOVA with Dunnett's multiple comparisons test vs. IDR-GFP. (D) Mean condensate sizes for R-K and VLIMFYW-S mutants and wild-type IDR-GFP were plotted by cumulative distribution frequency. Particle sizes were measured from three representative 20x fields using ImageJ. ns: not significant, two-tailed Mann-Whitney tests compared to IDR-GFP.

Supplemental Fig. 4. Systemic trafficking of TRBO vector. At 21 dpi, upper *N. benthamiana* systemic leaves were imaged at 488 nm. pJL-GFP and pJL-D/E-G:GFP were mostly restricted

to the petiole and midrib of systemic leaves. In contrast, pJL-p26:GFP invaded the lamina of systemic leaves. Images are representative of three independent experiments with at least four plants for each condition.

Figure 1

Click here to access/download;Figure;Fig1.TIF

Slide1.TIF

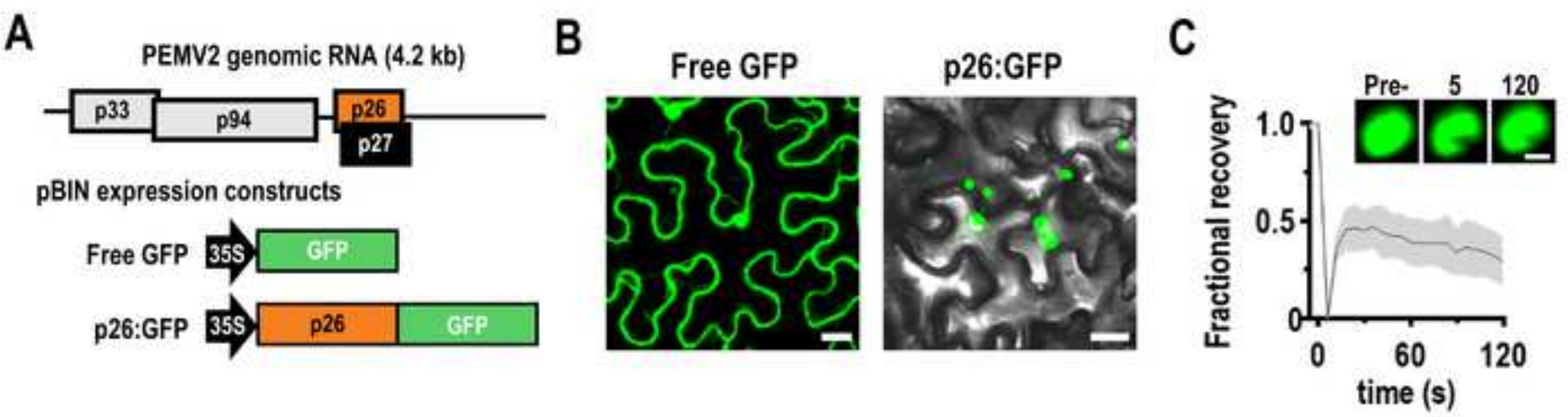

Figure 2

Click here to access/download;Figure;Fig2.TIF

Data_Figures - PLoSPathogens_REVISION.tif

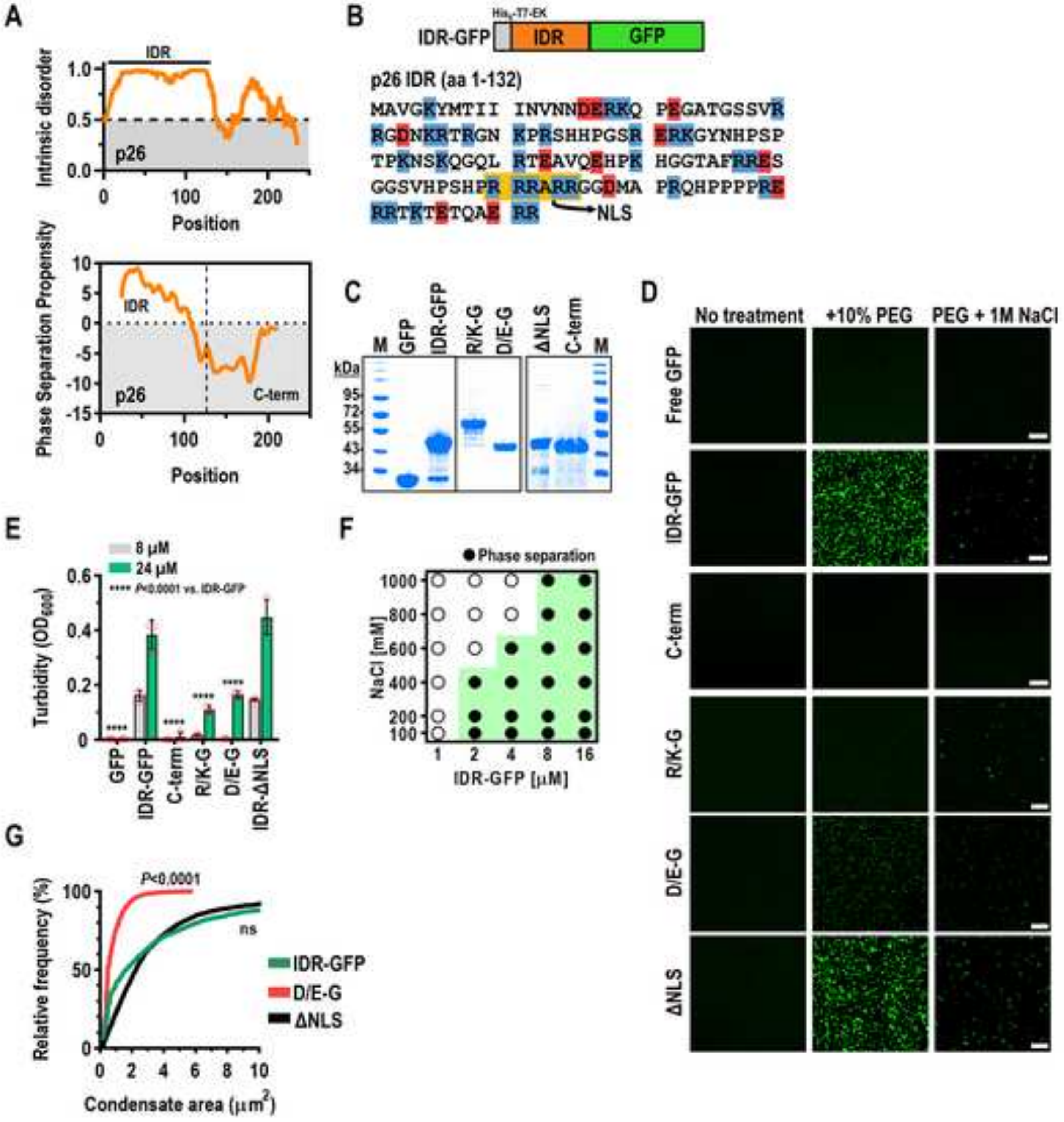

Figure 3

Click here to access/download;Figure;Fig3.TIF

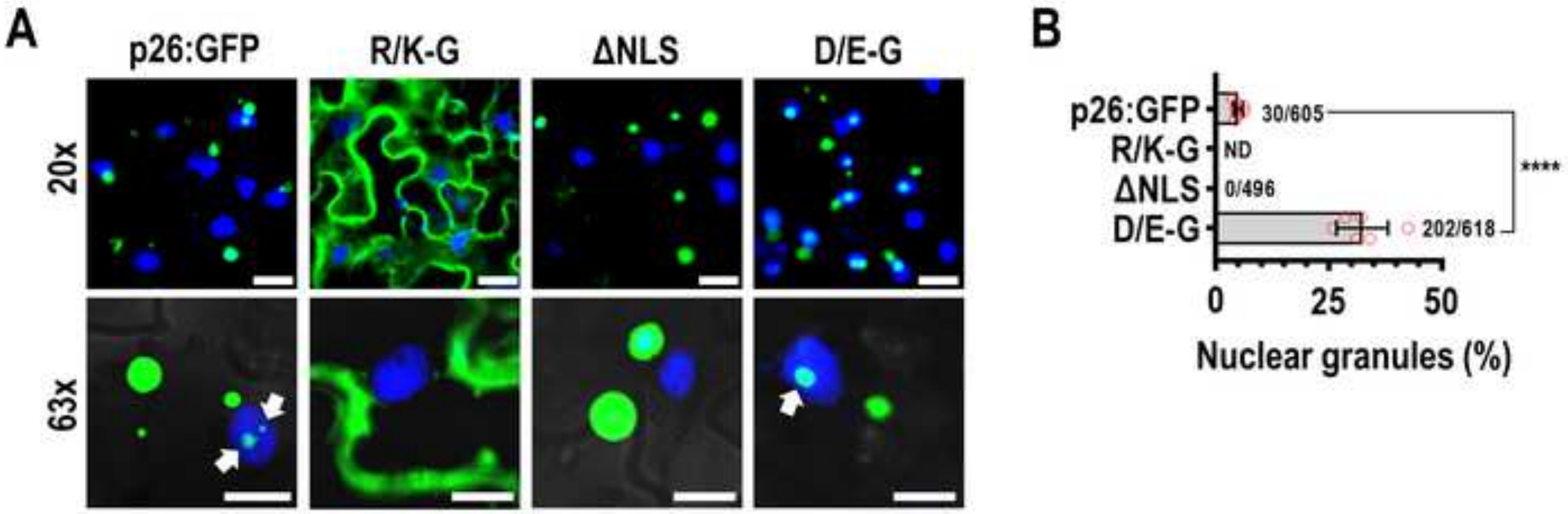

Slide3.TIF

Figure 4

Click here to access/download;Figure;Fig4.TIF

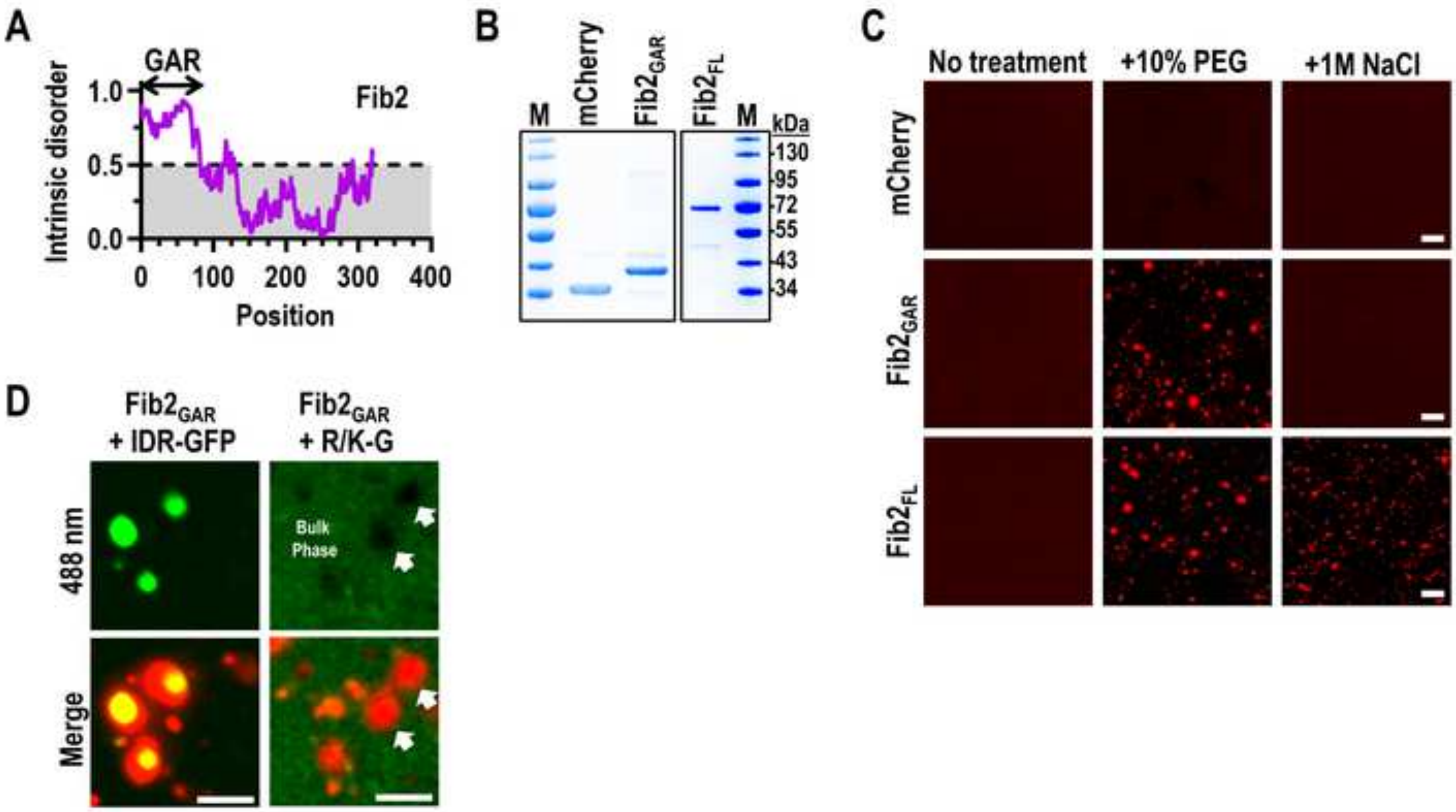

Slide4.TIF

Figure 5

Click here to access/download;Figure;Fig5.TIF

Slide5.TIF

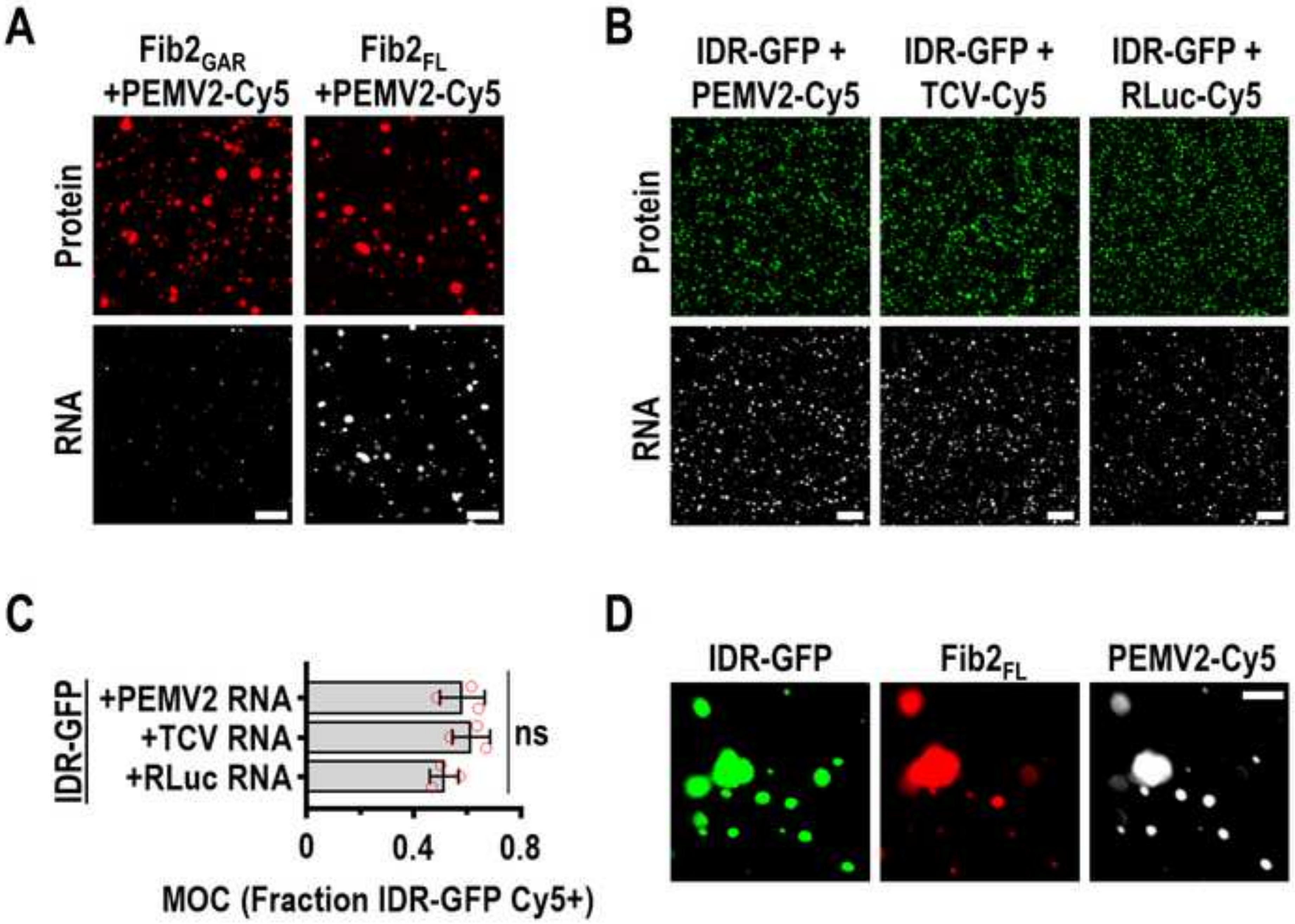

Click here to access/download;Figure;Fig6.TIF

Slide6.TIF

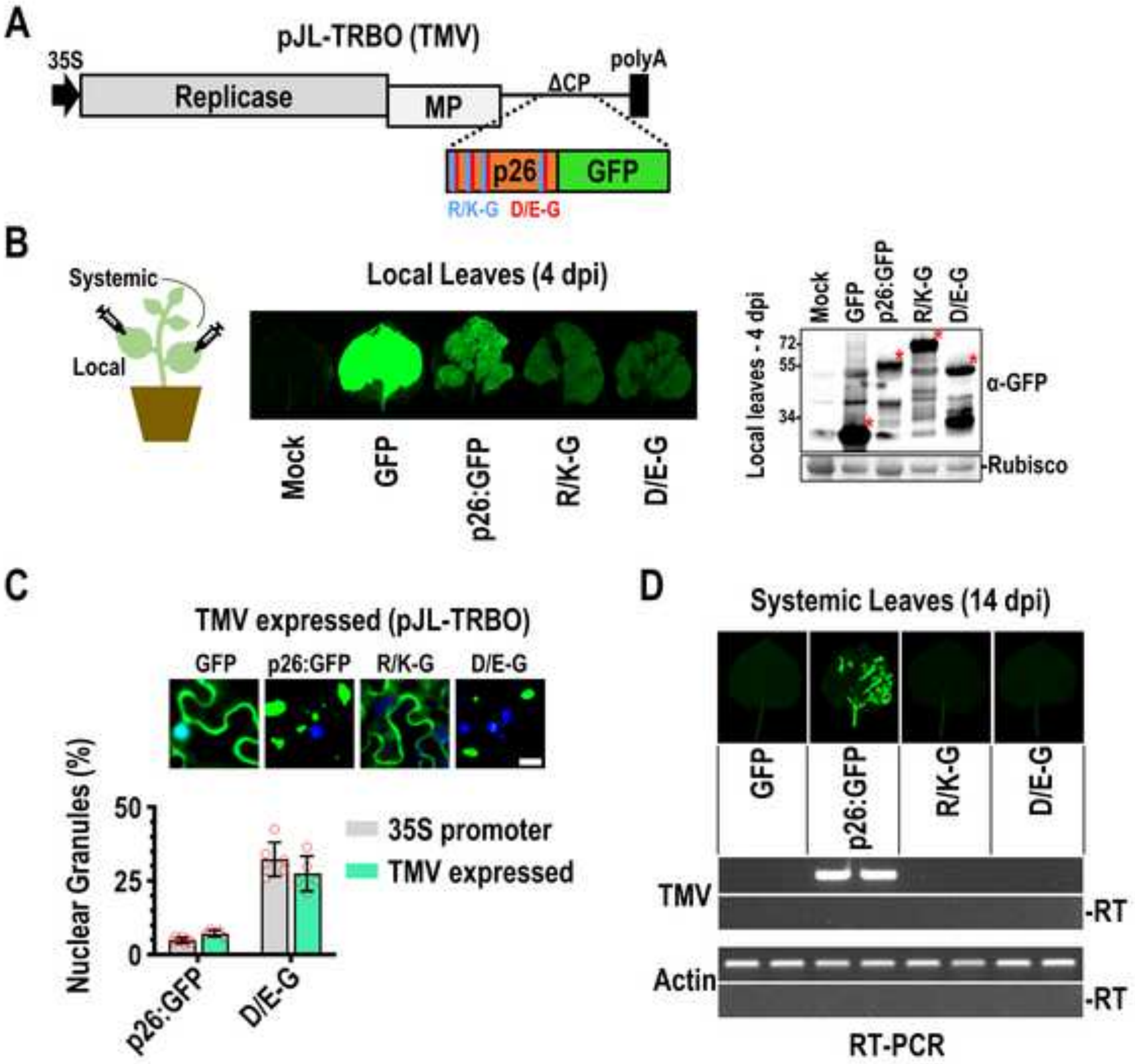

Figure 7

Click here to access/download;Figure;Fig7.TIF

Data_Figures - PLoSPathogens_REVISION.tif

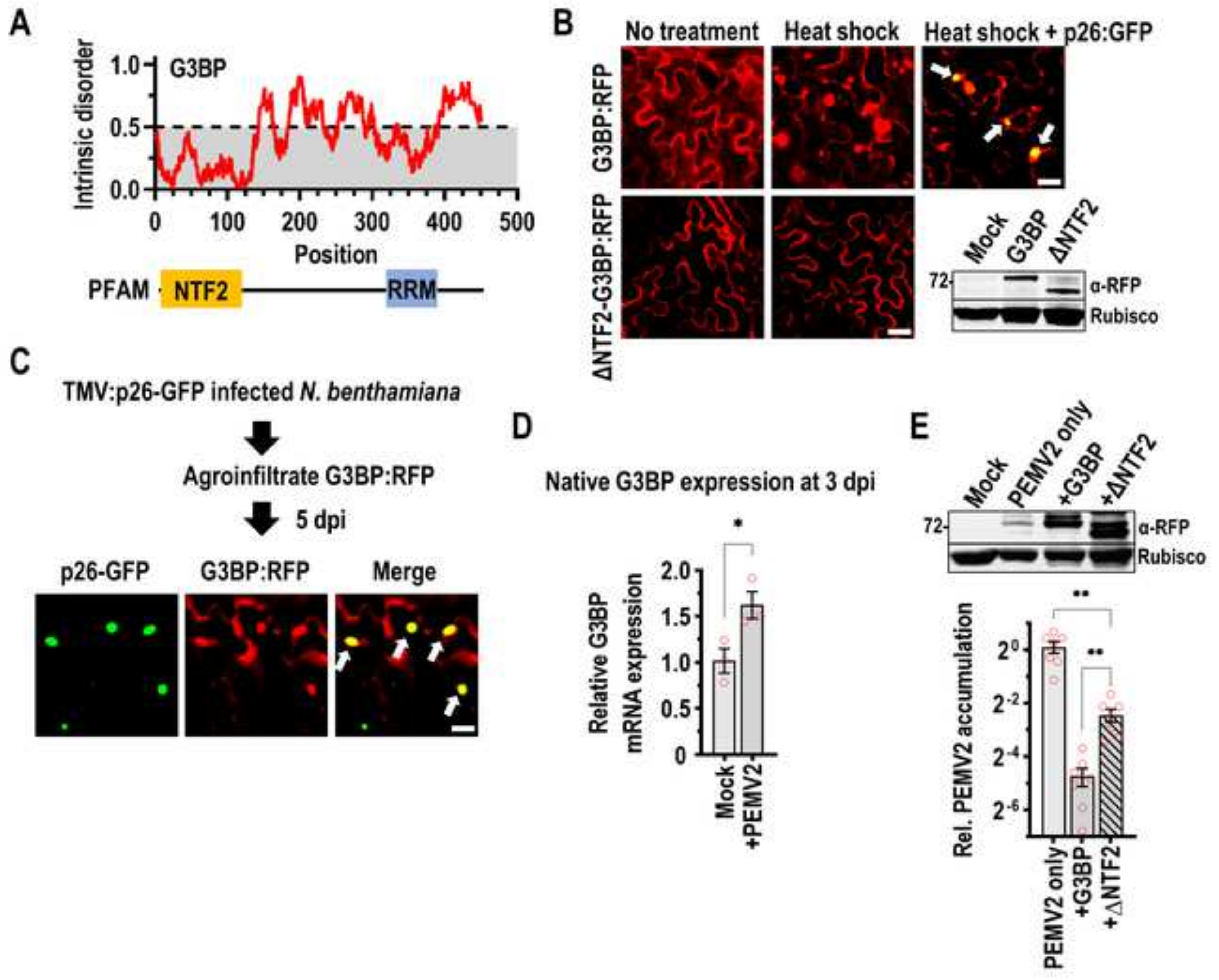

Figure S1

Click here to access/download
Supporting Information
FigS1.TIF

Figure S2

Click here to access/download
Supporting Information
FigS2.TIF

Figure S3

Click here to access/download
Supporting Information
FigS3.TIF

Figure S4

Click here to access/download
Supporting Information
FigS4.TIF

Click here to access/download
Supporting Information
Supplemental Information.docx

Phase separation of both a plant virus movement protein and cellular factors support virus-host interactions

Shelby L. Brown[1], Dana J. Garrison[1], and Jared P. May[1*]

[1]Department of Cell and Molecular Biology and Biochemistry, School of Biological and Chemical Sciences, University of Missouri-Kansas City, Kansas City, MO 64110, USA

*Correspondence: jpmay@umkc.edu

Short title: Plant virus protein phase separation

Keywords: phase separation, LLPS, liquid-liquid phase separation, RNA virus, stress granule, biomolecular condensate, fibrillarin, virus movement, virus replication, G3BP, virus-host interaction

ABSTRACT

Phase separation concentrates biomolecules, which should benefit RNA viruses that must sequester viral and host factors during an infection. Here, the p26 movement protein from *Pea enation mosaic virus 2* (PEMV2) was found to phase separate and partition in nucleoli and G3BP stress granules (SGs) *in vivo*. Electrostatic interactions drive p26 phase separation as mutation of basic (R/K-G) or acidic (D/E-G) residues either blocked or reduced phase separation, respectively. During infection, p26 must partition inside the nucleolus and interact with fibrillarin (Fib2) as a pre-requisite for systemic trafficking of viral RNAs. Partitioning of p26 in pre-formed Fib2 droplets was dependent on p26 phase separation suggesting that phase separation ~~of viral movement proteins~~ supports ~~nucleolar partitioning~~a critical virus-host interaction ~~and virus~~ required for virus movement. Furthermore, viral ribonucleoprotein complexes containing p26, Fib2, and PEMV2 RNAs were formed via phase separation *in vitro* and could provide the basis for self-assembly *in planta*. Interestingly, both R/K-G and D/E-G p26 mutants failed to support systemic trafficking of a *Tobacco mosaic virus* (TMV) vector in *Nicotiana benthamiana* suggesting that p26 phase separation, proper nucleolar partitioning, and systemic movement are intertwined. p26 also partitioned in SGs and G3BP over-expression restricted PEMV2 accumulation >20-fold. Expression of phase separation-deficient G3BP only restricted PEMV2 5-fold, demonstrating that phase separation enhances G3BP antiviral activity. ~~G3BP phase separation is critical for maximum antiviral activity.~~

AUTHOR SUMMARY

Phase separation of several cellular proteins is associated with forming pathological aggregates and exacerbating neurodegenerative disease progression. In contrast, roles for viral protein phase separation in RNA virus lifecycles are less understood. Here, we demonstrate that the p26 movement protein from *Pea enation mosaic virus 2* phase separates and partitions with phase-separated cellular proteins fibrillarin and G3BP. The related orthologue from

Groundnut rosette virus has been extensively studied and is known to interact with fibrillarin in the nucleolus as a pre-requisite for virus movement. We determined that basic residues and electrostatic interactions were critical for p26 phase separation and partitioning in pre-formed fibrillarin droplets. Furthermore, mutation of charged residues prevented ~~the rescue of a~~p26 from complementing a movement-deficient *Tobacco mosaic virus* vector in *Nicotiana benthamiana*. Stress granules form through phase separation and we found that p26 ~~could partition~~partitions inside stress granules following heat shock. Phase separation of the stress granule nucleator G3BP was required for maximum antiviral activity and constitutes a host response that ~~is dependent on~~requires ~~cellular protein~~ phase separation. ~~Collectively, we demonstrate that phase separation of a plant virus protein facilitates virus-host interactions that are required for virus movement and phase separation of cellular proteins can simultaneously restrict virus replication.~~ In summary, we found that phase separation of p26 and G3BP is necessary for pro-viral and anti-viral activities, respectively.

INTRODUCTION

Cellular organelles are membrane-bound compartments that are critical for eukaryotic cell function and RNA viruses often co-opt organelles to promote virus replication. Organelles exploited by RNA viruses include the endoplasmic reticulum (ER) [1], mitochondria [2], nucleus [3], and Golgi apparatus [4]. Recently, much attention has been directed towards membraneless organelles that form through protein phase separation. Phase separation transforms a single-phase solution into a dilute phase and droplet phase that concentrates biomolecules, such as proteins or RNAs [5, 6]. Some cellular proteins phase separate and form aggregates that are associated with several neurodegenerative disorders [7]. Proteins that undergo phase separation consistently contain intrinsically disordered regions (IDRs) that self-associate to form oligomers [8]. Many IDR-containing proteins have RNA-recognition motifs that non-specifically bind RNA and fine-tune phase separation by controlling material exchange, shape, and rigidity

of liquid droplets [8, 9]. Proteins that phase separate are often enriched in arginine residues that ~~can participate in cation-pi interactions with aromatic contacts and~~ promote phase separation through cation-pi interactions with aromatic contacts ~~-~~[10]. In addition, hydrophobic interactions can stabilize phase separations of low-complexity domains [11].

Membraneless organelles exist as liquids, gels, or solids, [12]. The most notable examples of liquid-liquid phase separated (LLPS) membraneless compartments are the nucleolus and cytoplasmic P-bodies ~~in the cytoplasm~~ [13]. Less dynamic stress granules (SGs) also form in the cytoplasm through phase separation and allow host cells to repress translation and influence messenger RNA (mRNA) stability in response to various stresses [14]. SGs are visible by microscopy within minutes following stress and contain Ras-GTPase-activating protein SH3 domain-binding protein 1 (G3BP1) that self-associates to induce SG formation [15]. SGs contain a stable inner core and an outer shell that is formed by weak electrostatic and/or hydrophobic interactions [16]. The G3BP1 inner core is resistant to dilution (atypical for LLPS) and has been ~~considered to be~~regarded as a form of liquid-solid demixing [17]. Interestingly, G3BP1 can have either pro-viral [18-20] ~~and~~ or anti-viral roles [21-23] in RNA virus lifecycles.

Members of the *Mononegavirales*, including *Rabies virus*, *Measles virus* (MeV), and *Vesicular stomatitis virus* generate phase-separated cytoplasmic inclusion bodies that ~~create~~ harbor viral factories [24-26]. Phase separation of MeV N and P proteins also promotes efficient encapsidation of viral RNAs [26]. Several groups have recently demonstrated that the nucleocapsid (N) protein from the novel SARS-CoV-2 coronavirus undergoes LLPS [27]. SARS-CoV-2 N protein phase separation is stimulated by the 5' end of its cognate RNA [28] and can partition into phase separations of heterogeneous nuclear ribonucleoproteins like TDP-43, FUS, and hnRNPA2 [29]. N protein phase separation has also been suggested to mediate nucleocapsid assembly and genome processing [30]. Finally, ~~The SARS-CoV-2~~ N protein ~~also~~ interacts with G3BP1 and can attenuate SG formation [31, 32].

Pea enation mosaic virus 2 (PEMV2) is a small (4,252 nt), positive-sense RNA plant virus ~~in the~~belonging to the ~~*tombusvirus*~~ *Tombusviridae* family and umbravirus genus. The PEMV2 long-distance movement protein ~~(MP)~~ p26 is required for systemic trafficking of viral RNA throughout an infected plant. Both p26 and the closely related umbravirus orthologue pORF3 from~~-~~ *Groundnut rosette virus* (GRV) primarily localize to the cytoplasm, but also target cajal bodies in the nucleus and eventually partition in the nucleolus [33-35]. Umbravirus ORF3 proteins must interact with nucleolar fibrillarin ~~(Fib2)~~, a pre-requisite for long-distance movement of viral RNA [35-37]. Additionally, the polerovirus *Potato leafroll virus* (PLRV) and the potexvirus *Bamboo mosaic virus* satellite RNA (satBaMV) encode proteins that must also localize to the nucleolus and interact with fibrillarin to support systemic movement [38-40]. Fibrillarin phase separates and forms the dense fibrillar component (DFC) of the nucleolus that shares a similar structure to SGs [16, 41]. Although the nucleolus itself is a phase separation and several plant virus proteins ~~co-localize~~interact with fibrillarin, the role of viral protein phase separation in plant virus lifecycles has not been investigated.

This study demonstrates that PEMV2 p26 undergoes phase separation both *in vitro* and *in vivo* and forms ~~highly viscous~~ poorly dynamic condensates. Viral ribonucleoprotein (vRNP) complexes containing p26, fibrillarin, and PEMV2 RNAs were reconstituted *in vitro* through phase separation ~~and likely represents~~and could represent the version of the *in vivo* event necessary for systemic trafficking. Charged residues played critical roles in p26 phase separation, nucleolar localization, and movement of a virus vector ~~Mutating charged residues required for phase separation and proper nucleolar localization blocked the movement of a viral vector~~ suggesting that phase separation and virus movement are intertwined. ~~Finally~~Finally, p26 ~~phase separates *in vivo* with the SG nucleator~~partitions in G3BP SGs~~, G3BP,~~ and G3BP over-expression ~~which~~ exhibits strong antiviral activity towards PEMV2. ~~PEMV2~~ Virus accumulation was largely restored during expression of a phase~~-~~-separation~~-~~-deficient G3BP, demonstrating that phase separation ~~of select cellular proteins aids~~ ~~enhances~~ ~~host antiviral responses.~~

Formatted: Font: Italic

enhances G3BP antiviral activity.

Formatted: Indent: First line: 0.5"

RESULTS

p26 forms poorly dynamic condensates *in vivo*. PEMV2 p26 and related umbravirus orthologues form large cytoplasmic ~~inclusion bodies~~granules during infection [37, 42, 43]. To visualize p26 granules, green fluorescent protein (GFP) was fused to the C-terminus of full-length p26 and expressed from the *Cauliflower mosaic virus* (CaMV) 35S promoter following agroinfiltration of *Nicotiana benthamiana* leaves (Fig. 1A). As a control, free GFP was expressed from the CaMV 35S promoter and failed to form granules but was evenly distributed throughout the cytoplasm and nucleus of the cell (i.e, outside of the large vacuole that comprises most of the cellular space) (Fig. 1B, Left). However, p26:GFP formed large cytoplasmic granules as previously observed (Fig. 1B, Right) [43]. To define the material properties of p26 ~~inclusion bodies~~granules *in vivo*, we used fluorescence recovery after photobleaching (FRAP) [44]. If p26 granules are highly dynamic liquid droplets, then FRAP recovery should be rapid and complete. Conversely, if p26 granules are solid aggregates, no fluorescence recovery is expected. Interestingly, p26:GFP granules recovered nearly 50% by 30 seconds post-bleach (Fig. 1C) demonstrating that p26 droplets have measurable fluidity. However, since p26:GFP failed to fully recover, our data suggests that p26 forms poorly dynamic condensates *in vivo* similar ~~free GFP was expressed from a 35S promoter and was evenly distributed throughout the cytoplasm and nucleus of the cell (i.e, outside of the large vacuole that comprises most of the cellular space) (Fig. 1B, Left). p26 with a C-terminal green fluorescent protein (GFP) tag was expressed from a *Cauliflower mosaic virus* (CaMV) 35S promoter in *Nicotiana benthamiana* by~~ following ~~agroinfiltration (Fig. 1A). Separately, free GFP was expressed from a 35S promoter and was evenly distributed throughout the cytoplasm and nucleus of the cell (i.e, outside of the large vacuole that comprises most of the cellular space) (Fig. 1B, Left). In contrast, p26:GFP formed large cytoplasmic inclusion bodies as previously~~

observed (Fig. 1B, Right) [43]. Nearly 50% recovery of p26:GFP was observed by 30 seconds post-bleach (Fig. 1C) demonstrating that p26 inclusion bodies have measurable fluidity. However, p26:GFP failed to recover any further suggesting that p26 forms poorly dynamic condensates *in vivo*, similar to what has been observed for G3BP1 SG cores [17].

p26 is intrinsically disordered and undergoes phase separation via electrostatic interactions. *In vitro* phase separation assays were performed to identify regions of p26 that drive phase separation as well as identify mutations that block phase separation. Since IDRs typically drive phase separation, the IUPred prediction model [45] was used to identify an arginine-rich disordered region spanning amino acids 1-132 of p26 (Fig. 2A, Top). The same region was also predicted to have the highest propensity to phase separate using the catGRANULE algorithm that was trained to identify proteins known to localize inform nuclear or cytoplasmic foci (Fig. 2A, Bottom) [46]. -To confirm the p26 IDR drives phase separation and subsequently identify mutations that block phase separation, the p26 IDR or a set of IDR mutants were fused to the N-terminus of GFP and purified from *Escherichia coli* (Fig. 2B and C). *In vitro* assays consisted of inducing phase separation of recombinant proteins with 10% PEG-8000 and observing phase separationdroplet formation via confocal microscopy or monitoringeasuring the solution turbidity (OD_{600}). Next, To support the *in vivo* FRAP observations suggesting that p26 undergoes phase separation, *in vitro* assays were performed. Using the IUPred disorder prediction model [45], a large IDR spanning amino acids 1-132 was predicted in p26 (Fig. 2A). For comparison, the non-essential PEMV2 cell-to-cell movement protein, p27, did not contain a predicted IDR (Fig. 2A). Glycine, proline, and arginine amino acids are the most abundant residues in the p26 IDR (Fig. 2B), consistent with disordered proteins known to phase separate [47]. tThe p26 IDRet was fused to the N-terminus of GFP and purified from *E. coli* for *in vitro* phase separation assaysin order (Fig. 2B and C). 10% PEG-8000 was used to induce phase separation andExpectedly, wild-type IDR-GFP readily phase separated as observed by both confocal microscopy (Fig. 2D) and turbidity assays (Fig. 2E)

and. In contrast, both free GFP and GFP fused to the C-terminal region of p26 (amino acids 133-226) failed to phase separate under all tested conditions (Fig. 2D and E). Note: aAll constructs presented in Fig.ure 2 contain N-terminal (His)tidine tags since the the presence of a His-tag did not influence IDR-GFP phase separation propensity, particle size, or or ability to sort RNAs into dropletsresistance to 1,6-hexanediol that selectively dissolves liquid condensates [48] -(Supplemental Fig. 1A-E). Similar findings have been reported for comparisons of untagged and His-tagged N protein from SARS-CoV-2Similar observations have been reported for His-tagged and tag-free SARS-CoV-2 N protein -[28]. Surprisingly, FRAP -Interestingly, two-minute FRAP recovery of IDR-GFP increased from 14% to 83%dramatically increased following tagHis-tag removal suggesting the His-tagthat histidine tracts can influence droplet dynamics *in vitro* (Supplemental Fig. 1F). Despite this, both tagged- and un-tagged IDR-GFP droplets were resistant to 10% 1,6-hexanediol that specifically dissolves liquid, but not gel-like condensates [48] suggesting that IDR-GFP droplets are highly viscous irrespective of the presence of a His-tag (Supplemental Fig. 1F and G).

Electrostatic interactions that support both protein self-association and phase separation but can be inhibited by high salt concentrations [49]. Therefore, to determine whether p26 phase separation is driven by electrostatic interactions, phase separation assays were performed with 1 M NaCl. IDR-GFP droplets were treated with 10% PEG-8000 was used to mimic cellular crowding and IDR-GFP readily phase separated under crowding conditions as observed by both turbidity assays (Fig. 2D) and confocal microscopy (Fig. 2E). In contrast, free GFP failed to phase separate under all tested conditions. 1M NaCl and sSignificantly reduced phase separation of IDR-GFP was observed by confocal microscopy (Fig. 2D). and 600 mM NaCl was sufficient to block IDR-GFP phase separation was sensitive towards NaCl in a dose-dependent manner as High-salt concentrations disrupt self-associations resulting from electrostatic interactions and can reverse phase separation [49]. Accordingly, IDR-GFP concentrations near the saturation concentration (C_{sat} = 4 2 µM) failed to phase separate in the presence 800 mM

Formatted: Indent: First line: 0.5"

~~NaCl and 1 M NaCl was required to block~~ suppress ~~phase separation under standard assay conditions using 8 µM protein~~ (Fig. 2~~E and~~ F). To confirm electrostatic interactions drive p26 phase separation, ~~IDR-GFP phase separations were next treated with 10% 1,6 hexanediol to probe the material properties of the *in vitro* condensates. 1,6 hexanediol interferes with weak hydrophobic protein-protein interactions and dissolves liquid-like, but not solid or highly viscous phase separations~~ [50]~~. IDR-GFP phase separations were resistant to 1,6 hexanediol treatment (Fig. 2E) and FRAP analyses revealed that IDR-GFP condensates only reached 13% recovery after 2 minutes following photo-bleaching (Fig. 2J). Together, these data suggest that the p26 IDR drives phase separation through electrostatic interactions and the resulting condensates are highly viscous.~~

~~**Charged residues are critical for efficient p26 IDR phase separation.** To determine if specific groups of amino acids contribute to p26 phase separation, a series of IDR-GFP mutants were purified (Fig. 2C) and tested. First,~~ all basic or acidic residues were mutated to glycine (R/K-G or D/E-G, respectively). ~~Since high-salt blocks IDR-GFP phase separation, simultaneous mutation of either basic or acidic residues was predicted to inhibit phase separation.~~ Indeed, R/K-G failed to phase separate while D/E-G showed significantly reduced phase separation compared to IDR-GFP when examined by confocal microscopy (Fig. ~~2G~~2D), turbidity assays (Fig. ~~2H~~2E), or mean condensate size ~~when measured using standard assay conditions with 8 µM protein~~ (Fig. ~~2I~~2G). At ~~elevated~~ higher concentrations (24 µM), R/K-G formed non-uniform aggregates, ~~and failed to recover in FRAP assays (Fig. 2J). However~~whereas~~,~~ D/E-G formed uniform droplets (Supplemental Fig. 2).~~condensates displayed significantly elevated fluidity when compared to IDR-GFP with 35% recovery after 2 minutes (Fig. 2J) and may be due to increased glycine content that has been associated with increasing condensate fluidity~~ [51]~~.~~ A more subtle ~~arginine~~ mutation was tested by deleting the sequence 5'-RRRARR-3' (amino acids 100-105) that constitutes a~~the~~ conserved nuclear localization signal (NLS) ~~5'-RRRARR-3'~~ first identified in GRV pORF3 [52]. ΔNLS phase separated with equal propensity to wild-type (Fig.

2D and E) demonstrating that the highly conserved NLS ~~arginines are~~is not required for ~~p26~~ phase separation. This finding is somewhat unsurprising since the NLS only accounts for 16% (5/31) of the basic residues within the IDR. Finally, potential cation-pi ~~and~~or hydrophobic interactions were disrupted by mutating all arginines to lysines (R-K) or all hydrophobic residues to serine (VLIMFYW-S), respectively. ~~all arginines were mutated to lysine (R-K) to prevent cation-pi interactions while a separate mutation prevented hydrophobic interactions by mutating all hydrophobic residues to serine (VLIMFYW-S).~~ Both R-K and VLIMFYW-S mutants phase separated with equal propensity ~~as the wild-type IDR~~to wild-type demonstrating cation-pi and hydrophobic interactions are not required for p26 phase separation (Supplemental Fig. 3). Together, these results demonstrate that the N-terminal IDR drives p26 phase separation through electrostatic interactions.

~~Cation-pi interactions between arginines and aromatic rings promote phase separation and are useful for predicting the propensity of a protein to phase separate~~ [10, 53]~~. However, the p26 IDR only contains three aromatic residues that could potentially facilitate cation-pi interactions and mutation of all arginines to lysine (R-K) had no effect on phase separation, condensate size, or FRAP recovery (Fig. 2G-J).turbidity or particle size (Fig. 2E and G). Finally, hydrophobic IDR residues (V, L, I, M, F, Y, W) were mutated to polar serine residues to reduce the hydrophobicity and prevent hydrophobic interactions that can also drive phase separation~~ [11]~~. Again, VLIMFYW-S phase separated like wild-type and was sensitive to high-saltand wild-type IDR did not differ in propensity to phase separate or particle size (Fig. 2G2E and G). However, VLIMFYW-S condensates failed to recover in FRAP assays (Fig. 2J). These results suggest that hydrophobic residues contribute to the limited fluidity of p26 phase separations or rather the observed decrease in fluidity is due to the hardening properties of introduced serine residues~~ [51]~~.~~

Formatted: Font: Bold

Formatted: Font: Bold

Formatted: Font: Bold

Formatted: Font: Bold

Formatted: Font: Bold

Formatted: Font: Bold

Formatted: Font: Bold

~~p26 partitions in the nucleolus and forms assemblies with the fibrillarin GAR domain via phase separation~~Charged residues govern p26 nucleolar partitioning.

Formatted: Font: Bold

Formatted: Font: Bold

Umbravirus movement proteins must access the nucleolus to support systemic virus trafficking [35]. Here, the nucleolar partitioning of full-length wild-type or mutant p26:GFP was examined after agroinfiltration of *N. benthamiana* leaves with constructs expressing p26:GFP from a CaMV 35S promoter. As previously reported for related orthologues [35-37, 52], p26 was observed in ~~the nucleolus and cajal bodies~~nuclear bodies (e.g. nucleolus) in addition to forming cytoplasmic granules appearing as droplets (Fig. 3A~~, Left~~). Supporting our *in vitro* observations, ~~However, R/K-G p26~~full-length p26 containing glycine substitutions for all basic residues (R/K-G) did not form phase-separated granules but instead was ~~instead~~ diffusely expressed throughout the cytoplasm and failed to partition in the nucleolus (Fig. 3A)~~, Middle)~~. Expectedly, ~~Conserved arginines in the related GRV pORF3 were previously shown to constitute a nuclear localization signal (NLS)~~ [52]~~. d~~Deletion of the conserved NLS resulted in strictly cytoplasmic localization of p26. Since ΔNLS formed phase-separated droplets ~~with droplets appearing similar to wild-type~~ but failed to enter the nucleolus, our data demonstrates that ~~and B~~p26 ~~phase separation~~phase separation of p26 alone is ~~not~~ insufficient for nucleolar localization~~and conserved arginine tracts are necessary for nucleolar entry~~. Despite reduced phase separation of the D/E-G IDR *in vitro*, full-length p26 containing glycine substitutions for all acidic residues (D/E-G) formed ~~Therefore, both p26 nuclear localization and phase separation are controlled by arginine residues and based on our mutagenesis studies it is unlikely that phase separation can be abolished without disrupting the NLS. Despite having markedly reduced phase separation *in vitro*, D/E-G p26 localized to the nucleolus and formed~~ cytoplasmic granules that appeared like wild-type (Fig. 3A~~, Right~~). However, ~~D/E-G had increased nucleolar retention compared to wild-type p26 as determined using the Manders Overlap Coefficient (MOC) to measure the degree of spatial overlap between D/E-G and DAPI-stained nuclei (Fig. 3B)~~33% of D/E-G granules localized to the nucleus compared to only 5% of wild-type p26 granules (Fig. 3B) suggesting

that the net charge of p26 influences nucleolar localization. The overall net charge of D/E-G at pH 7.4 is +36 compared to +14 for wild-type ~~p26~~ and ~~previous research~~our findings support earlier work~~ has demonstrated~~ that showed nucleolar localization of ~~of ribosomal proteins and the *Human immunodeficiency virus 1* Tat protein~~cellular and viral proteins ~~is~~was dependent on the overall positive charge ~~of the protein~~ [54, 55]~~. Nucleolar localization/retention of *Arabidopsis thaliana* ribosomal proteins is dependent on the overall positive (basic) charge of the protein~~ [54]~~ and could explain the increased retention of D/E-G since the net charge of D/E-G at pH 7.4 is +36 compared to +14 for wild-type p26. Similarly, nucleolar accumulation of the *Human immunodeficiency virus 1* Tat protein strongly correlates with the overall net charge~~ [55].

p26 phase separation is required for partitioning in Fib2 droplets. Fibrillarin (Fib2) is a ~~known~~ host factor required for systemic trafficking of umbravirus vRNPs [33, 34] and makes up the dense fibrillar component of the nucleolus [56]. The *A. thaliana* Fib2 N-terminus contains an intrinsically disordered glycine- and arginine-rich (GAR) domain (Fig. ~~3C~~4A) that is common to fibrillarin across eukaryotes [57]. To determine whether the GAR domain of *A. thaliana* Fib2 is sufficient for Fib2 phase separation, the GAR domain (amino acids 7-77, Fib2$_{GAR}$) was fused to the N-terminus of mCherry and purified from *E. coli* for *in vitro* phase separation assays (Fig. ~~3D~~4B). Full-length Fib2 was also fused to mCherry (Fib2$_{FL}$) for comparison. Free mCherry did not phase separate in the presence of 10% PEG-8000 or under high-salt conditions (Fig. ~~3E~~4C). Fib2$_{GAR}$ readily phase separated under crowding conditions but was unable to phase separate in the presence of 1 M NaCl (Fig. ~~3E~~4C). These results indicate that the GAR domain is sufficient to drive Fib2 phase separation through electrostatic interactions and is consistent with findings using mammalian or *Caenorhabditis elegans* fibrillarin [41, 58, 59]. Full-length Fib2 phase separated under crowding conditions but unlike Fib2$_{GAR}$, Fib2$_{FL}$ was resistant to 1 M NaCl (Fig. ~~3E~~4C). These results suggest that Fib2$_{FL}$ condensates are not strictly dependent on electrostatic interactions or Fib2$_{FL}$ ~~forms~~ can form ~~aggregates that are resistant to high salt~~salt-resistant

Formatted: Indent: First line: 0"

Formatted: Font: Bold

aggregates. ~~Indeed, Fib2$_{FL}$ condensates failed to recover in FRAP assays while Fib2$_{GAR}$ droplets were poorly dynamic but recovered nearly 20% after two minutes (Fig. 3F). Earlier work has determined that the GAR domain increases the solid-like properties of fibrillarin condensates~~ [58] ~~and supports our observations that both Fib2$_{GAR}$ and Fib2$_{FL}$ are poorly dynamic.~~

During an infection, p26 must presumably partition in pre-formed Fib2 droplets in the dense fibrillar component of the nucleolus [41] to support virus movement. Therefore, we sought to determine whether phase separation of p26 was required for partitioning in Fib2 droplets. Fib2 functions as a *scaffold* for recruiting *client* proteins into the phase separated nucleolus, and by nature, scaffolds should be present in excess relative to clients for partitioning to occur [60, 61]. Thus, a 1:6 molar ratio of IDR-GFP:Fib2$_{GAR}$ was used in the following experiments. Fib2$_{GAR}$ was chosen since the related GRV pORF3 directly interacts with the Fib2 GAR domain [36]. Expectedly, IDR-GFP was readily sorted into pre-formed Fib2$_{GAR}$ droplets *in vitro* (Fig. 4D, Left) and is likely the reconstituted version of the p26-Fib2 interaction required for Fib2 export from the nucleus and subsequent association with viral RNAs [35]. To determine whether phase separation of p26 was required for Fib2 partitioning, the phase separation-deficient R/K-G mutant was added to pre-formed Fib2$_{GAR}$ droplets. Interestingly, R/K-G remained in the bulk phase and was excluded from Fib2$_{GAR}$ droplets (Fig. 4D, Right, white arrows). These results demonstrate that p26 phase separation is critical for interactions with phase-separated Fib2 and strongly support a role for phase separation in PEMV2 movement.

vRNPs required for systemic trafficking can be reconstituted ~~*in vitro*~~ via phase separation. Movement-competent umbravirus vRNPs consist of Fib2, p26, and genomic RNAs ~~Fib2 is a necessary component of umbravirus vRNPs that move systemically during infection~~[36]. Therefore, we sought to determine whether vRNPs could be re-constituted *in vitro* through phase separation. First, t~~T~~o determine whether full-length PEMV2 RNA could be sorted to Fib2 droplets, Cy5-labelled PEMV2 RNA was mixed with pre-formed Fib2$_{GAR}$ or Fib2$_{FL}$

droplets at a ~~500:1~~1:500 ~~protein:RNA~~RNA:Fib2 molar ratio. This ratio was used since earlier work ~~showed~~ determined that umbravirus RNAs ~~could be~~were saturated by ~~viral MPs~~protein interactors under these conditions [34, 42]. PEMV2-Cy5 RNA was not efficiently sorted into Fib2$_{GAR}$ droplets (Fig. ~~3F~~5A) and is consistent with earlier findings that determined the GAR domain does not bind RNA [57, 58]. However, Fib2$_{FL}$ efficiently captured PEMV2-Cy5 RNAs demonstrating that ~~viral~~ PEMV2 RNAs can partition ~~with~~ in Fib2 phase separations (Fig. ~~3F~~5A). Since p26 must also ~~bind PEMV2 RNA prior to trafficking~~associate with viral RNAs, PEMV2-Cy5 RNAs ~~was~~ were mixed with pre-formed IDR-GFP droplets again using a 1:500 RNA:protein ratio that saturates viral RNA with ~~MP~~p26. Approximately 50% of IDR-GFP signal spatially overlapped PEMV2-Cy5 signal when visualized by confocal microscopy and quantified by MOC (Fig. 5B~~3G~~ and ~~H~~C). Interestingly, partitioning of ~~viral RNA~~RNAs inside IDR-GFP condensates was not unique to PEMV2 RNAs since the distantly related *Turnip crinkle virus* (TCV)~~ RNA~~ and non-viral *Renilla* luciferase (RLuc) RNAs were~~as~~ sorted to IDR-GFP phase separations with ~~similar~~ equal propensity (Fig. ~~3H~~ 5B and ~~I~~C). Importantly, the N-terminal His-tag of IDR-GFP did not influence RNA sorting into droplets (Supplemental Fig. 1G). ~~Collectively, these results demonstrate that both cognate and non-cognate viral RNAs are readily sorted into p26 phase separations.~~

~~Since the related GRV pORF3 directly interacts with the Fib2 GAR domain [36], IDR-GFP was added to pre-formed Fib2$_{GAR}$ droplets at a 1:6 molar ratio to determine whether p26 can partition into phase separated Fib2 condensates. relative~~ [60, 61]~~ Expectedly, IDR-GFP was readily sorted into pre-formed Fib2$_{GAR}$ droplets *in vitro* (Fig. 3I, Left) and is likely the reconstituted version of the p26-Fib2 interaction required for Fib2 export from the nucleus and subsequent vRNA association~~ with viral RNAs~~[35]. To determine whether phase separation of p26 supports Fib2 partitioning, the phase separation-deficient R/K-G mutant was added to pre-formed Fib2$_{GAR}$ droplets. Interestingly, R/K-G remained in the bulk phase and was excluded from Fib2$_{GAR}$ droplets (Fig. 3I, Right, White~~ white ~~arrows) suggesting that the ability of p26 to~~

Formatted: Font: Not Italic

Formatted: Not Highlight

~~phase separate supports the key interaction with Fib2 required for virus movemen~~Finally, ~~t.~~ ~~Finally, Fib2$_{FL}$ and IDR-GFP~~equimolar Fib2$_{FL}$ and IDR-GFP were mixed with PEG to form droplets prior to the addition of PEMV2-Cy5 RNAs at a 1:500 molar ratio. Equimolar amounts of Fib2$_{FL}$ and IDR-GFP were used since atomic force microscopy revealed that Fib2 and GRV pORF3 form ring-like complexes with equimolar composition [33].~~-~~ Droplets containing IDR-GFP, Fib2$_{FL}$, and PEMV2 RNAs were observed (Fig. ~~5E~~5D) and demonstrates that movement-competent vRNPs can be reconstituted ~~using *in vitro*~~by phase separation ~~*assays*~~*in vitro*. Together, these findings su~~pport a role for p26 phase separation in virus movement~~ggest that phase separation of Fib2 and p26 could support the formation of movement-competent vRNPs *in planta*.

Formatted: Font: Italic

Phase separation-deficient p26 mutants fail to systemically traffic a virus vector. To determine whether phase separation-deficient p26 mutants could support virus trafficking, a movement-deficient *Tobacco mosaic virus* (TMV) vector was used to express free GFP, p26, R/K-G, or D/E-G GFP fusions (Fig. ~~4A~~6A). The TMV vector (pJL-TRBO) contains a coat protein (CP) deletion that has been previously reported to block systemic movement [62]. ~~Interestingly,~~~~Previously~~However, previous work has demonstrated that GRV pORF3 ~~and PEMV2 p26 have been previously shown to~~ can ~~systemically traffic TMV when expressed from a subgenomic promoter in place of CP~~~~can complemen~~~~was shown to~~ can support long-distance movement of TMV when co-expressed ~~with a~~alongside a movement-deficient TMV vector [63]. Furthermore, both native p26 and p26:GFP can systemically traffic TMV when expressed ~~in place of CP from~~ from a subgenomic promoter in place of CP [43, 64]~~ and remains functional when fused to GFP [43].~~ [43]~~First, l~~L~~L~~ocal infections were established in young *N. benthamiana* plants (4th leaf stage) and high levels of free GFP and lower levels of p26:GFP, R/K-G, and D/E-G were observed at 4 days post-infiltration (dpi) (Fig. ~~4B~~6B). Localization patterns of p26:GFP, R/K-G, and D/E-G did not differ when expressed from either a 35S promoter or a TMV vector and ~~demonstrated~~confirmed that D/E-G granules were significantly enriched in nuclei compared

to wild-type p26 during virus infection (Fig. 6C). As expected, s~~S~~ystemic ~~trafficking~~ movement of TMV by ~~:~~p26:GFP was readily apparent by 14 dpi by both visual inspection of leaves and RT-PCR whereas free GFP did not move TMV systemically (Fig. ~~4C~~6~~C~~D). Since R/K-G p26 can neither phase separate nor enter the nucleolus, R/K-G expectedly failed to systemically traffic TMV at 14 dpi (Fig. 6~~C~~D). Surprisingly, ~~However, TMV expressing GFP, R/K-G, or D/E-G GFP fusions failed to move systemically at 14 dpi. Basic amino acids are known to function as a NLS for GRV pORF3~~ [52] ~~and are also required for partitioning in pre-formed Fib2 droplets (Fig. 3J). Therefore, p26 nucleolar localization and phase separation are co-dependent on basic residues and the R/K-G mutation presumably blocks interactions with Fib2 and subsequent virus trafficking. Failure of~~ D/E-G p26 also failed to support ~~virus~~ TMV movement at 14 dpi despite the ability ~~was surprising since D/E-G retained the ability~~ to phase separate (albeit less efficiently *in vitro*) and localize to the nucleolus ~~(Figs. 2G and 3A)~~. However, drastically increased nucleolar retention ~~(>5-fold)~~ of D/E-G ~~could~~ likely contributed to the block in systemic movement and suggests that nucleolar and virus trafficking by p26 is a tightly regulated process. Together, these data suggest that p26 phase separation, nucleolar partitioning, and virus movement are connected and co-dependent on charged residues. The TMV CP deletion has been previously reported to block systemic movement of the TRBO vector [62], but we routinely observed systemic trafficking of pJL-GFP after 3 weeks (Supplemental Fig. ~~1~~4). However, pJL-GFP was largely restricted to the petiole and midrib of systemic leaves whereas pJL-p26:GFP spread throughout the veins and invaded the lamina. ~~Weak D/E-G GFP expression was observed in the petioles and midribs of upper leaves at 21 dpi while R/K-G GFP was not visible (Supplemental Fig. 1).~~

p26 is sorted into G3BP phase separations that restrict PEMV2 accumulation. Our findings suggest that p26 phase separations are poorly dynamic and share ~~share~~ similar material properties to G3BP SG cores ~~, mostly consistent with liquid-solid demixing~~ [17]. Since SGs can have both pro-viral and antiviral roles in RNA lifecycles, we investigated whether p26

could partition in G3BP SGs. A NTF2-RRM domain-containing protein from *A. thaliana* (AtG3BP) functions as a G3BP-like SG nucleator in plants [65]. ~~The~~ In mammals, the N-terminal NTF2 domain (Fig. 7A) is required for both phase separation and recruitment to SGs [66, 67] ~~and G3BP contains downstream IDRs (Fig. 5A)~~. As previously demonstrated by Krapp et. al. [65], G3BP:RFP displays a diffuse cytoplasmic expression pattern under no stress, but forms cytoplasmic SGs after heat shock (Fig. ~~5B~~7B). As expected, ΔNTF2-G3BP failed to phase separate and form SGs following heat shock (Fig. ~~5B~~7B). When co-expressed with p26:GFP, recruitment of p26 to G3BP SGs was observed following heat shock (Fig. ~~5B~~7B) demonstrating that p26 can partition in phase-separated SGs. To determine ~~if~~ whether p26 partitions into SGs during a viral infection, G3BP:RFP was ~~expressed in~~agroinfiltrated into *N. benthamiana* ~~plants~~ plants systemically infected with TMV expressing p26:GFP (Fig. ~~5C~~7C). p26:GFP condensates co-localized with G3BP:RFP demonstrating that p26 and G3BP can share phase separations during an authentic viral infection (Fig. ~~5C~~7C). ~~To determine if G3BP expression is up- or down-regulated during PEMV2 infection~~Next, native G3BP ~~gene~~ expression was measured by RT-qPCR at 3 dpi in PEMV2-infected *N. benthamiana* leaves ~~(Fig. 5D). PEMV2 infection led to a~~ and revealed a 61% increase during infection ~~in G3BP expression~~that could be part the anti-viral host response (Fig. ~~5D~~7D~~) in accordance with~~). ~~previous RNA-seq analyses that showed a 2-fold increase in G3BP expression under similar conditions [43].~~ To ~~determine if G3BP exerts a pro- or~~confirm G3BP has an ~~anti-viral~~inhibitory effect on PEMV2 accumulation, G3BP:RFP was ~~over-expressed alongside~~co-infiltrated with PEMV2 into *N. benthamiana*. At 3 dpi, PEMV2 accumulation was reduced >20-fold ~~during~~ by G3BP over-expression demonstrating that G3BP exerts strong antiviral activity towards PEMV2 (Fig. ~~5E~~7E). Virus accumulation was largely restored (only 5-fold inhibition) during overexpression of ΔNTF2-G3BP ~~demonstrating~~ indicating that phase separation of G3BP is required for maximal antiviral activity (Fig. ~~5E~~7E). Together, these data demonstrate that p26 partitions inside G3BP SGs and ~~G3BP~~ phase separation of G3BP ~~facilitates an antiviral virus-host interaction~~enhances antiviral activity towards PEMV2.

DISCUSSION

Phase separation of viral proteins has largely been associated with negative-sense RNA viruses that use proteins that undergo phase separation to form virus factories [26], including Negri bodies during Rabies virus infections [24, 68, 69]. Also, measles virus N and P proteins encapsidate viral RNA more efficiently in a phase-separated droplet compared to a single phase solution [70]. In contrast, formation of phase-separated virus factories during positive-strand RNA virus infections have not been described.many positive-strand RNA viruses However, many many positive-strand RNA viruses, including members of the *Tombusviridae* family form membranous replication organelles to concentrate virus replication complexes [71, 72]. Although limited evidence for phase separation of plant virus proteins exists [73], aA recent study demonstrated that *Turnip mosaic virus* inhibits the formation of phase-separated nuclear dicing bodies (D-bodies) that are responsible for microRNA processing and anti-viral defense [74, 75]. While these findings demonstrate plant viruses have evolved to suppress certain cellular phase separations, However, the role ofexamples of plant virus proteins using phase separation to support virus-host interactions have not been reported. phase separation of viral proteins in virus-host interactions in plants has not been investigated. While specific roles for phase separation of positive-sense RNA virus proteins in the virus lifecycle remain limited, phase separation of the SARS-CoV-2 N protein has been suggested to mediate nucleocapsid assembly and genome processing [30].

This study demonstrates that the N-terminal IDR of p26 drives phase separation of poorly dynamic condensates through electrostatic interactions. Phase separation of p26 was abolished by mutating all basic residues to glycine (R/K-G) both *in vitro* and *in vivo*. p26 movement protein from the positive-sense RNA plant virus PEMV2 phase separates to form poorly dynamic condensates. Electrostatic interactions between acidic and basic IDR residues

Formatted: Indent: First line: 0.5"

drive p26 phase separation and mutation of basic residues (R/K-G) abolished phase separation. Surprisingly, mutation of acidic residues (D/E-G) did not abolish phase separation but was significantly reduced *in vitro* compared to wild-type *in vitro*. Previous studies have found that phase separation of arginine-rich peptides can occur through charge repulsion in the presence of buffer counteranions and could explainsupport D/E-G phase separation [76, 77]. Mutation of charged residues resulted in altered nucleolar localization of p26. Both deletion of the conserved p26 NLS (5' RRRARR 3') and R/K-G mutations blocked nucleolar localization. However, ΔNLS phase-separated with equal propensity to wild-type p26 demonstrating that phase separation alone is insufficient for p26 nucleolar partitioning. Interestingly, nucleolar retention of D/E-G p26 granules was >5-fold higher compared to wild-type p26 and was likely the result of increased protein net charge [55].

p26 must interact with fibrillarin (Fib2 Fib2) in phase-separated nucleoli to support systemic virus trafficking [36], but the role of phase separation in this interaction was previously unknown. and conserved arginine residues have been shown to function as a NLS for the related GRV pORF3 [52]. Using *in vitro* assays with pre-formed Fib2 droplets, we demonstrated that the wild-type IDR, but not the R/K-G mutant could partition in Fib2 droplets. These observations suggest that p26 phase separation is required for systemic movement since p26 likely encounters pre-formed Fib2 droplets when first entering the nucleolus during infection. Indeed, R/K-G p26 failed to support systemic movement of a TMV vector but it remains unclear whether the block in systemic movement was due to R/K-G's inability to phase separate, enter the nucleolus, or a combination of both. Our results demonstrated that p26 nuclear localization and phase separation are both governed by basic amino acids making it problematic to separate these phenomena. However, the R/K-G IDR failed to accumulate in pre-formed Fib2$_{GAR}$ droplets *in vitro* suggesting that phase separation of p26 could be required to partition in Fib2 phase separations and the nucleolus. Unsurprisingly, R/K-G p26 failed to support systemic movement of a TMV vector demonstrating that nucleolar partitioning, and potentially phase

~~separation is required for virus movement. Mutation of acidic residues (D/E-G) significantly increased nucleolar retention of p26 and could be the result of increased protein net charge that is known to correlate with increased nucleolar retention~~ [55]~~. Interestingly~~Surprisingly, D/E-G p26 also failed ~~to systemically traffic a~~ move a TMV vector which could be attributed to the drastic increase in nucleolar retention of D/E-G p26. In summary, our findings demonstrate that ~~suggesting that the interplay between p26 nucleolar localization and virus movement is tightly regulated. In summary,~~ charged amino acids play ~~a critical role~~critical roles in p26 phase separation, nucleolar partitioning, and systemic virus movement.

Stress granules can support or restrict RNA virus replication and are assembled by the self-association and phase separation of G3BP [66, 67]. Seven *A. thaliana* G3BP-like candidates have been identified [78] and share an N-terminal NTF2 domain that is required for phase separation of mammalian G3BP1 [67]. In this study, the previously characterized AtG3BP-2 (AT5G43960) [65] was used to determine whether p26 could partition in G3BP stress granules. After heat shock, p26 readily partitioned inside G3BP SGs and both p26 and G3BP co-localized during virus infection. G3BP expression was upregulated during PEMV2 infection suggesting that G3BP could be expressed as part of a concerted host response to infection. PEMV2 infection was severely restricted by the over-expression of G3BP but was ~~G3BP over-expression severely restricted PEMV2 infection but was~~ partially restored during expression of ΔNTF2-G3BP, demonstrating that phase separation of G3BP ~~is necessary for maximum~~enhances antiviral activity towards PEMV2.

Formatted: Indent: First line: 0"

Since PEMV2 accumulation was not fully restored during ΔNTF2-G3BP expression, G3BP retains measurable antiviral activity in the dilute state. Human G3BP1 has been shown to bind and promote the degradation of mRNAs with structured 3’ untranslated regions (3’ UTRs) in conjunction with upframeshift 1 (Upf1) as part of the structure-mediated RNA decay (SRD) pathway [79]. PEMV2 contains a highly structured 3’ UTR [80] and like many RNA viruses is inhibited by Upf1 [81, 82]. Therefore, G3BP over-expression could enhance SRD targeting of

PEMV2 RNAs. It remains ~~unclear if~~unknown whether p26 partitioning into G3BP SGs is beneficial or detrimental for PEMV2 replication. However, p26 disrupts the Upf1-dependent nonsense-mediated decay (NMD) pathway [43] and Upf1 is known to partition in G3BP1 SGs [83]. Partitioning of p26 into G3BP SGs ~~could potentially~~has the potential to interfere with Upf1- or G3BP-dependent RNA decay pathways.

In summary, our findings demonstrate that a plant virus movement protein phase separates and partitions inside cellular phase separations, namely the nucleolus and SGs. Since nucleolar partitioning is required for virus trafficking and G3BP SG formation severely restricts PEMV2 replication, our findings highlight both beneficial and detrimental virus-host interactions mediated by phase separation.

ACKNOWLEDGEMENTS

We would like to thank Dr. Björn Krenz (Leibniz Institut DSMZ, Brunswick, Germany) for the generous gift~~s~~ of the G3BP:RFP construct. We would also like to thank Dr. Jonathan Dinman and Dr. Anne Simon (University of Maryland) for their thoughtful insight. We would also like to thank Dr. Anne Simon for critically reading this manuscript.

AUTHOR CONTRIBUTIONS

Conceptualization, J.P.M; Methodology, S.B. and J.P.M; Investigation, S.B. and J.P.M; Writing – Original Draft, J.P.M.; Writing – Review & Editing, S.B. and J.P.M; Supervision, J.P.M.

COMPETING INTERESTS

The authors declare no competing interests.

MATERIALS & METHODS

Construction of binary plant expression vectors. The pBIN61S binary vector was used to express proteins of interest from the constitutive *Cauliflower mosaic virus* (CaMV) 35S promoter. p26:GFP, R/K-G, D/E-G, and ΔNLS GFP-fusions were PCR-amplified from synthetic double-stranded DNA fragments (Integrated DNA Technologies) and cloned into pBIN61S using the *BamH*I and *Sal*I restriction sites. R/K-G and D/E-G p26:GFP fusions contain glycine substitutions for all basic or acidic p26 residues, respectively. pBIN61S-GFP has been previously described [84]. p26:GFP, R/K-G, and D/E-G GFP fusions were also PCR amplified and cloned into pBIN61 using *BamH*I and *Sal*I restriction sites to transiently express p26-fusions downstream of the constitutive *Cauliflower mosaic virus* (CaMV) 35S promoter. G3BP:RFP was a generous gift from Dr. Björn Krenz and has been previously described [65]. To construct ΔNTF2-G3BP:RFP, G3BP-RFP was PCR amplified with amino acids 2-125 of G3BP omitted. PCR amplification introduced forward *BamH*I and reverse *Sal*I restriction sites for cloning into pBIN61S. All DNA constructs used in this study were sequenced for accuracy.

Agroinfiltration and plant growth. All plant eExpression constructs used in this study were electroporated into *Agrobacterium tumerfaciens* (C58C1 strain). Liquid cultures were passaged in media containing the appropriate antibiotics and 20 µM acetosyringone 1 day prior to infiltration. Overnight cultures were pelleted and resuspended in 10 mM $MgCl_2$, 10 mM MES-K [pH 5.6], and 100 µM acetosyringone. Infiltration mixturesAll agroinfiltrations contained the p14 RNA silencing suppressor from *Pothos latent virus* [85] at a final OD_{600} of 0.2. Typically, the 3rd-5th leaves from young *N. benthamiana* plants were infiltrated with a 1 mL syringe. pBIN-GFP constructs, TMV vectors, and G3BP:RFP constructs were infiltrated at a final OD_{600} of 0.4. The full-length PEMV2 expression construct has been previously described [81] and was agroinfiltrated at a final OD_{600} of 0.4. Visualization of nuclei in p26:GFP, R/K-G, or D/E-G-expressing plants agroinfiltrated leaves was achieved by infiltrating a solution of 5 µg/mL DAPI (4′,6-diamidino-2-phenylindole) into leaves 45 minutes prior to imaging. *Heat shock of G3BP-*

Formatted: Font: Italic

Field Code Changed

Formatted: Indent: First line: 0.5"

Field Code Changed

Formatted: Superscript

Formatted: Superscript

Field Code Changed

Formatted: Font: Italic

*expressing plants was performed by placing plants at 37°C for 45 minutes prior to imaging. To visualize G3BP:RFP alongside p26:GFP during virus infection, young N. benthamiana plants (3-4 leaf stage) were first infiltrated with TMV:p26:GFP. After strong p26:GFP signal was observed in the systemic leaves (typically ~2-3 weeks), G3BP:RFP was agroinfiltrated and imaged at 5 dpi using a Zeiss LSM 510 Meta confocal microscope with a 20x objective. P*N. benthamiana plants were grown in a humidity-controlled chamber at 24°C, 65% humidity, and 12-hour day/night schedule (200 µmol $m^{-2}s^{-1}$).

Fluorescence recovery after photobleaching (FRAP). pBIN61S containing p26:GFP was agroinfiltrated into *N. benthamiana* using an OD_{600} = 0.4. GFP fluorescence was visible after 2 days and leaves expressing p26:GFP were wet-mounted and imaged using a Zeiss LSM 510 Meta confocal microscope with a 20X objective and Zen 2009 software. FRAP was performed by photobleaching aA ~2 µm diameter region was photobleached with 100% laser power (488 nm) with subsequent fluorescence recovery measured at 5 s intervals. Background regions and unbleached reference condensates were recorded as controls. FRAP was performed using a Zeiss LSM 510 Meta confocal microscope with a 20X objective and Zen 2009 software. Data analysis was performed as previously described [86]. Briefly, background intensity was subtracted, intensities were normalized to set the first post-bleach value to zero and presented as a fraction of the pre-bleach fluorescence intensity.

Construction of bacterial expression vectors. For C-terminal GFP GFP-fusion recombinant protein production in *E. coli*, pRSET his-eGFP [87] was used as a backbone and was a gift from Jeanne Stachowiak (Addgene plasmid # 113551). All recombinant proteins purified in this study contained N-terminal histidine-tags for affinity chromatography. WildThe wild-type p26 IDR (amino acids 1-132) or p26 C-terminus (amino acids 133-2236) was were PCR amplified from a full-length PEMV2 infectious clone, whereas . Note: the last 10 amino

Formatted: Font: Italic

Formatted: Indent: First line: 0.5"

Formatted: Subscript

acids of p26 were omitted from the C-term construct to circumvent proteolysis encountered during bacterial expression (not shown). Mutant IDRs containing ~~R-K, VLIMFYW-S,~~ R/K-G, ~~and~~ D/E-G, or ΔNLS mutations were synthesized (Integrated DNA Technologies) as double stranded DNA fragments and were used in restriction digests and ligation reactions using T4 DNA Ligase (New England Biolabs). R/K-G and D/E-G mutants contain glycine substitutions for all basic or acidic residues, respectively. ΔNLS is missing the sequence 5'-RRRARR-3' (amino acids 100-105) within the IDR. ~~The R-K IDR mutation substituted lysine (K) for all arginines (R) to prevent cation-pi interactions. Finally, VLIMFYW-S contains serine (S) substitutions for all hydrophobic residues.~~ ~~All fragments except for R/K-G and D/E-G~~Wild-type IDR, ~~R-K, and VLIMFYW-S~~ ~~were~~was cloned into the *BamH*I restriction site of pRSET his-eGFP and sequenced for directionality and accuracy. C-term, R/K-G, ~~and~~ D/E-G, and ΔNLS were cloned into pRSET his-eGFP using both the *Nhe*I and *BamH*I restriction sites and sequenced for accuracy.

Fibrillarin (Fib2) was first PCR amplified from cDNA synthesized from *Arabidopsis thaliana* seedling total RNA using primers Forward 5'-GCAGCAGCTAGCATGAGACCTCCTCTAACTGGAAGTGG-3' and Reverse 5'-CTGCTGCGGATCCAGCAGCAGTAGCAGCCTTTGGCTTC-3' where the underlined sequences denote the *Nhe*I and *BamH*I restriction sites used to clone the PCR fragment into pRSET-his-mCherry [88], a gift from Jeanne Stachowiak (Addgene plasmid # 113552). The resulting construct is full-length Fib2 with a C-terminal mCherry fusion (Fib2$_{FL}$). The Fib2 GAR domain was PCR amplified from Fib2$_{FL}$, digested, and ligated into the *Nhe*I and *BamH*I restriction sites of pRSET-his-mCherry to generate Fib2$_{GAR}$. Both constructs contain N-terminal histidine tags for affinity purification.

~~The *Tobacco mosaic virus* (TMV) expression vector pJL-TRBO has been previously described~~ [62]~~ and was a gift from John Lindbo (Addgene plasmid # 80082). The TMV vector containing p26:GFP has also been previously described~~ [43]~~. R/K-G and D/E-G GFP-fusion~~

Formatted: Font: Arial

inserts were commercially synthesized (Integrated DNA Technologies). TMV vectors expressing free GFP, R/K-G or D/E-G GFP fusions were constructed by cloning respective PCR fragments into the *PacI* and *NotI* restriction sites in pJL-TRBO. p26:GFP, R/K-G, and D/E-G GFP fusions were also PCR amplified and cloned into pBIN61 using *BamHI* and *SalI* restriction sites to transiently express p26-fusions downstream of the constitutive *Cauliflower mosaic virus* (CaMV) 35S promoter. G3BP:RFP was a generous gift from Dr. Björn Krenz and has been previously described [65]. To construct ΔNTF2-G3BP:RFP, G3BP-RFP was PCR amplified with amino acids 2-125 of G3BP omitted. PCR amplification introduced forward *BamHI* and reverse *SalI* restriction sites for cloning into pBIN61S. All DNA constructs used in this study were sequenced for accuracy.

Fluorescence recovery after photobleaching (FRAP). A ~2 µm diameter region was photobleached with 100% laser power with subsequent recovery measured at 5 s intervals. Background regions and unbleached reference condensates were recorded as controls. FRAP was performed using a Zeiss LSM 510 Meta confocal microscope with a 20X objective and Zen 2009 software. Data analysis was performed as previously described [86]. Briefly, background intensity was subtracted, intensities were normalized to set the first post-bleach value to zero and presented as a fraction of the pre-bleach fluorescence intensity.

Formatted: Indent: First line: 0.5"

Protein expression and purification. Histidine-tagged recombinant proteins were expressed in BL21(DE3) *E. coli* (New England Biolabs) using autoinduction Luria-Bertani (LB) broth and purified using HisPur™ cobalt spin columns (Thermo Scientific). Proteins were purified under denaturing conditions according to the manufacturer's protocol using 8 M urea. All equilibration, wash, and elution buffers contained 1 M NaCl to suppress phase separation. Following elution of recombinant proteins from the cobalt resin, proteins were re-folded through dialysis in buffer containing 10 mM Tris-HCl (pH 7.0), 300 mM NaCl, 1 mM EDTA, 1 mM dithiothreitol, and 10% glycerol as previously done used for the related pORF3 from *Groundnut*

rosette virus [42]. Urea was removed in a stepwise fashion by using dialysis buffers containing 4 M Urea, 1 M Urea, or no Urea. Proteins were concentrated using centrifugal filters and concentrations were measured using ~~the~~ a Bicinchoninic acid (BCA) protein assay ~~kit~~ (Millipore Sigma). Protein integrity and purity was assessed by SDS-PAGE. If necessary, hydrophobic interaction chromatography (Methyl HIC resin) was used to further purify and concentrate GFP-fusion samples according to the manufacturers protocol (Bio-Rad). ~~Proteins were aliquoted and stored at -80°C.~~

~~*Phase*~~ *In vitro phase separation assays*. ~~GFP- or mCherry-tagged proteins were first expressed and purified from *E. coli* before used in *in vitro* assays. In this study~~For *in vitro* assays, recombinant proteins were used at a final concentration of 8 µM unless otherwise noted in the figures or text. Phase separation assays consisted of the following mixture: 8 µM protein, 10 mM Tris-HCl (pH 7.~~0~~5), 1 mM DTT, 100 mM NaCl, and 10% PEG-8000 to ~~mimic cellular crowding~~induce phase separation. Phase separation occurred rapidly and samples were directly loaded onto glass slides for confocal microscopy using a Zeiss LSM 510 Meta confocal microscope with a 20x objective and appropriate filters. High-salt conditions included NaCl at a final concentration of 1 M and “no treatment” did not include PEG-8000. Phase separation assays were performed at least twice across two protein preparations. Turbidity assays comparing IDR-GFP ~~and D/E-G~~with controls or IDR mutants were performed with either 8 µM or 24 µM protein under standard assay conditions. 100 µL reactions were placed at room temperature for 15 minutes prior to ~~measuring~~ OD_{600} measurements using a 96-well plate reader. ImageJ was used to measure droplet size (condensate area) from thresholded images (20x objective) using the built-in “analyze particles” tool.

RNA sorting assays. Cy5-labelled PEMV2 or TCV RNA was synthesized by T7 run-off transcription using *Sma*I-linearized full-length infectious clones. Cy5-labelled *Renilla* luciferase (RLuc) RNAs were synthesized from PCR products containing a T7 promoter, RLuc ORF, and a 13-nt 3’ untranslated region. Cy5-UTP (APExBIO) was added to *in vitro* transcription reactions

Formatted: Font: Italic

Formatted: Indent: First line: 0.5"

according to the HiScribe T7 Quick High Yield RNA Synthesis Kit protocol (New England Biolabs). RNAs were included in phase separation assays at a final concentration of 16 nM ~~(500:1 protein:RNA~~(1:500 RNA:protein ratio). Mander's overlap coefficients (MOC) were used to measure the fraction of IDR-GFP that was positive for Cy5-labelled RNA from 20x fields of view using the ImageJ plugin EzColocalization [89].

~~*Agroinfiltration*. Expression constructs were electroporated into *Agrobacterium tumorfaciens* (C58C1 strain). Liquid cultures were passaged in media containing 20 µM acetosyringone 1 day prior to infiltration. Overnight cultures were pelleted and resuspended in 10 mM $MgCl_2$, 10 mM MES-K [pH 5.6], and 100 µM acetosyringone. Infiltration mixtures contained the p14 RNA silencing suppressor from *Pothos latent virus*~~ [85] ~~at a final OD_{600} of 0.2. pBIN-GFP constructs, TMV vectors, and G3BP:RFP constructs were infiltrated at a final OD_{600} of 0.4. The full-length PEMV2 expression construct has been previously described~~ [81] ~~and was agroinfiltrated at a final OD_{600} of 0.4. Visualization of nuclei in p26:GFP, R/K-G, or D/E-G-expressing plants was achieved by infiltrating a solution of 5 µg/mL DAPI (4′,6-diamidino-2-phenylindole) into leaves 45 minutes prior to imaging. Heat shock of G3BP-expressing plants was performed by placing plants at 37°C for 45 minutes prior to imaging. To visualize G3BP:RFP alongside p26:GFP during virus infection, young *N. benthamiana* plants (3-4 leaf stage) were first infiltrated with TMV:p26:GFP. After strong p26:GFP signal was observed in the systemic leaves (typically ~2-3 weeks), G3BP:RFP was agroinfiltrated and imaged at 5 dpi using a Zeiss LSM 510 Meta confocal microscope with a 20x objective. Plants were grown in a humidity-controlled chamber at 24°C, 65% humidity, and 12-hour day/night schedule (200 µmol $m^{-2}s^{-1}$).~~

Construction and agroinfiltration of ~~of~~ Tobacco mosaic virus (TMV) ~~expression~~ vectors. The TMV vector pJL-TRBO has been previously described [62] and was a gift from John Lindbo (Addgene plasmid # 80082). The TMV vector containing p26:GFP has also been previously described [43]. R/K-G and~~,~~ D/E-G ~~, and ΔNLS~~ GFP-fusions were PCR amplified from synthetic

Field Code Changed

Field Code Changed

Formatted: Indent: First line: 0.5"

DNA fragments with introduced *Pac*I and *Not*I restriction sites for digestion and ligation into the corresponding pJL-TRBO sites. R/K-G and D/E-G constructs contain full-length p26 with glycine substitutions for all basic or acidic residues, respectively. Both R/K-G and D/E-G contain a C-terminal GFP tag. Constructs were sanger sequenced for accuracy.

TMV movement assay and RT-PCR. pJL-TRBO derived TMV vectors expressing GFP or p26-GFP fusions were agroinfiltrated (OD_{600} = 0.4) into young *N. benthamiana* plants (~~3-4~~3rd-4~~ true~~th true leaf stage). GFP fluorescence in local and systemic leaves was monitored daily. At 4 dpi, robust local infections were evident, and leaves were imaged (488 nm) prior to grinding in liquid nitrogen. Total protein was extracted by resuspending leaf tissue in 1X PBS supplemented with 3% β-mercaptoethanol and protease inhibitor cocktail (Thermo Scientific). Samples were mixed with 6X Laemmli SDS buffer, boiled, and separated by SDS-PAGE. A semi-dry transfer method was used to transfer proteins to nitrocellulose for western blotting using anti-GFP antibodies (Life technologies) at a 1:5000 dilution. Anti-rabbit IgG conjugated with horseradish peroxidase was used as a secondary antibody again ~~at~~ with a 1:5000 dilution. Blots were visualized using the Pierce enhanced chemiluminescence kit (Thermo Scientific). Systemic leaves were harvested at 14 dpi for total RNA extraction using Trizol. 100 ng total RNA was digested with RQ1 DNase (Promega) and served as template for reverse transcription using iScript supermix (~~Bio~~ Bio-Rad). No reverse transcriptase controls (-RT) were Included for all sample and primer sets. 1 µL cDNA was used as template for 25 cycles of PCR using GoTaq polymerase (Promega) targeting the TMV replicase using forward primer 5' CCGCGAATCTTATGTGGAAT 3' and reverse primer 5' TCCTCCAAGTGTTCCCAATC 3'. *N. benthamiana* actin was amplified by 31 cycles of PCR as a loading control with forward primer 5' TCCTGATGGGCAAGTGATTAC 3' and reverse primer 5' TTGTATGTGGTCTCGTGGATTC 3'.

G3BP expression and visualization. G3BP expression constructs were agroinfiltrated into *N. benthamiana* plants at an OD_{600} = 0.4 alongside p14. Heat shock of G3BP-expressing

Formatted: Subscript

Formatted: Superscript

Formatted: Font: Italic

Formatted: Subscript

plants was performed by incubating plants at 37°C for 45 minutes prior to imaging. To determine whether p26:GFP partitions in G3BP SGs, pBIN-p26:GFP was co-infiltrated with G3BP:RFP 2-3 days prior to heat shock. To visualize G3BP:RFP alongside p26:GFP during virus infection, young *N. benthamiana* plants (3-4 leaf stage) were first infiltrated with TMV:p26:GFP. After strong p26:GFP signal was observed in the systemic leaves (typically ~2-3 weeks), G3BP:RFP was agroinfiltrated and imaged at 5 dpi using a Zeiss LSM 510 Meta confocal microscope with a 20x objective. The full-length PEMV2 expression construct has been previously described [81] and was agroinfiltrated alongside full-length G3BP or ΔNTF2-G3BP at a final OD_{600} of 0.2. Using the same protocol as above, western blotting with anti-RFP antibodies (Thermo Scientific, 1:5000 dilution) was performed to measure full-length G3BP or ΔNTF2 expression levels following agroinfiltration.

RT-qPCR. Agroinfiltrated "spots" were cut from leaves and stored at -80ºC. Samples were ground in liquid nitrogen and total RNA was extracted using the Quick-RNA Plant Kit (Zymo Research). An on-column DNase I step was added using RQ1 DNase (Promega). Total RNAs were used as templates for SYBR green-based one-step reverse-transcriptase quantitative PCR (RT-qPCR) using the NEB Luna One-Step RT-qPCR kit (New England Biolabs). All primers were validated by standard curve analysis and had PCR efficiencies ranging from 90-110%. Native *N. benthamiana* G3BP (Transcript ID: Niben101Scf03456g00002.1) was targeted using primers Forward 5' TAGGGGAAGCAATCCAGATG 3' and Reverse 5' TCCTTATCGATCCCAACAGC 3'. PEMV2 genomic RNA was targeted by forward primer 5' TTGCAAGGTTCTAGGCATCC 3' and reverse primer 5' CAACGATCGAAAAAGACGATG 3'. Gene expression was normalized to the internal control transcripts from the agroinfiltrated p14 RNA silencing suppressor using forward primer 5' TCCCAAACAGGGGTTTTATG 3' and reverse primer 5' GGTAATTGGGAACCCTCGAT 3'. Expression analyses were performed by the ΔΔCq method using Bio-Rad CFX Maestro

Formatted: Indent: First line: 0.5"

software. Target fidelity was monitored by melt curve analyses and no reverse transcriptase controls.

REFERENCES

1. Inoue T, Tsai B. How viruses use the endoplasmic reticulum for entry, replication, and assembly. Cold Spring Harb Perspect Biol. 2013;5(1):a013250-a.

2. Anand SK, Tikoo SK. Viruses as modulators of mitochondrial functions. Adv Virol. 2013;2013:738794-.

3. Walker EJ, Ghildyal R. Editorial: Viral Interactions with the Nucleus. Front Microbiol. 2017;8:951-.

4. Miller S, Krijnse-Locker J. Modification of intracellular membrane structures for virus replication. Nature Reviews Microbiology. 2008;6(5):363-74.

5. Dolgin E. What lava lamps and vinaigrette can teach us about cell biology. Nature. 2018;555(7696):300-2.

6. Tang L. Liquid phase separation. Nature Methods. 2019;16(1):18-.

7. Elbaum-Garfinkle S. Matter over mind: Liquid phase separation and neurodegeneration. The Journal of biological chemistry. 2019;294(18):7160-8.

8. Drino A, Schaefer MR. RNAs, Phase Separation, and Membrane-Less Organelles: Are Post-Transcriptional Modifications Modulating Organelle Dynamics? BioEssays. 2018;40(12):1800085.

9. Zhang H, Elbaum-Garfinkle S, Langdon EM, Taylor N, Occhipinti P, Bridges AA, et al. RNA Controls PolyQ Protein Phase Transitions. Mol Cell. 2015;60(2):220-30.

10. Vernon RM, Chong PA, Tsang B, Kim TH, Bah A, Farber P, et al. Pi-Pi contacts are an overlooked protein feature relevant to phase separation. Elife. 2018;7:e31486.

11. Murthy AC, Dignon GL, Kan Y, Zerze GH, Parekh SH, Mittal J, et al. Molecular interactions underlying liquid-liquid phase separation of the FUS low-complexity domain. Nature structural & molecular biology. 2019;26(7):637-48.

12. Boeynaems S, Alberti S, Fawzi NL, Mittag T, Polymenidou M, Rousseau F, et al. Protein Phase Separation: A New Phase in Cell Biology. Trends in cell biology. 2018;28(6):420-35.

13. Shorter J. Phase separation of RNA-binding proteins in physiology and disease: An introduction to the JBC Reviews thematic series. The Journal of biological chemistry. 2019;294(18):7113-4.

14. Riback JA, Katanski CD, Kear-Scott JL, Pilipenko EV, Rojek AE, Sosnick TR, et al. Stress-Triggered Phase Separation Is an Adaptive, Evolutionarily Tuned Response. Cell. 2017;168(6):1028-40.e19.

15. Matsuki H, Takahashi M, Higuchi M, Makokha GN, Oie M, Fujii M. Both G3BP1 and G3BP2 contribute to stress granule formation. Genes to cells : devoted to molecular & cellular mechanisms. 2013;18(2):135-46.

16. Jain S, Wheeler JR, Walters RW, Agrawal A, Barsic A, Parker R. ATPase-Modulated Stress Granules Contain a Diverse Proteome and Substructure. Cell. 2016;164(3):487-98.

17. Wheeler JR, Matheny T, Jain S, Abrisch R, Parker R. Distinct stages in stress granule assembly and disassembly. Elife. 2016;5:e18413.

18. Cristea IM, Rozjabek H, Molloy KR, Karki S, White LL, Rice CM, et al. Host factors associated with the Sindbis virus RNA-dependent RNA polymerase: role for G3BP1 and G3BP2 in virus replication. Journal of virology. 2010;84(13):6720-32.

19. Götte B, Panas MD, Hellström K, Liu L, Samreen B, Larsson O, et al. Separate domains of G3BP promote efficient clustering of alphavirus replication complexes and recruitment of the translation initiation machinery. PLoS Pathog. 2019;15(6):e1007842.

20. Hosmillo M, Lu J, McAllaster MR, Eaglesham JB, Wang X, Emmott E, et al. Noroviruses subvert the core stress granule component G3BP1 to promote viral VPg-dependent translation. Elife. 2019;8.

21. Yang W, Ru Y, Ren J, Bai J, Wei J, Fu S, et al. G3BP1 inhibits RNA virus replication by positively regulating RIG-I-mediated cellular antiviral response. Cell death & disease. 2019;10(12):946.

22. Pandey K, Zhong S, Diel DG, Hou Y, Wang Q, Nelson E, et al. GTPase-activating protein-binding protein 1 (G3BP1) plays an antiviral role against porcine epidemic diarrhea virus. Veterinary microbiology. 2019;236:108392.

23. Reineke LC, Kedersha N, Langereis MA, van Kuppeveld FJ, Lloyd RE. Stress granules regulate double-stranded RNA-dependent protein kinase activation through a complex containing G3BP1 and Caprin1. mBio. 2015;6(2):e02486.

24. Nikolic J, Le Bars R, Lama Z, Scrima N, Lagaudrière-Gesbert C, Gaudin Y, et al. Negri bodies are viral factories with properties of liquid organelles. Nature communications. 2017;8(1):58.

25. Zhou Y, Su JM, Samuel CE, Ma D. Measles Virus Forms Inclusion Bodies with Properties of Liquid Organelles. Journal of virology. 2019;93(21).

26. Heinrich BS, Maliga Z, Stein DA, Hyman AA, Whelan SPJ. Phase Transitions Drive the Formation of Vesicular Stomatitis Virus Replication Compartments. mBio. 2018;9(5).

27. Cascarina SM, Ross ED. A proposed role for the SARS-CoV-2 nucleocapsid protein in the formation and regulation of biomolecular condensates. FASEB journal : official publication of the Federation of American Societies for Experimental Biology. 2020.

28. Iserman C, Roden CA, Boerneke MA, Sealfon RSG, McLaughlin GA, Jungreis I, et al. Genomic RNA Elements Drive Phase Separation of the SARS-CoV-2 Nucleocapsid. Mol Cell. 2020;80(6):1078-91.e6.

29. Perdikari TM, Murthy AC, Ryan VH, Watters S, Naik MT, Fawzi NL. SARS-CoV-2 nucleocapsid protein phase-separates with RNA and with human hnRNPs. EMBO J. 2020;39(24):e106478.

30. Carlson CR, Asfaha JB, Ghent CM, Howard CJ, Hartooni N, Safari M, et al. Phosphoregulation of Phase Separation by the SARS-CoV-2 N Protein Suggests a Biophysical Basis for its Dual Functions. Mol Cell. 2020;80(6):1092-103.e4.

31. Li J, Guo M, Tian X, Wang X, Yang X, Wu P, et al. Virus-Host Interactome and Proteomic Survey Reveal Potential Virulence Factors Influencing SARS-CoV-2 Pathogenesis. Med. 2020.

32. Nabeel-Shah S, Lee H, Ahmed N, Marcon E, Farhangmehr S, Pu S, et al. SARS-CoV-2 Nucleocapsid protein attenuates stress granule formation and alters gene expression via direct interaction with host mRNAs. bioRxiv : the preprint server for biology. 2020:2020.10.23.342113.

33. Canetta E, Kim SH, Kalinina NO, Shaw J, Adya AK, Gillespie T, et al. A plant virus movement protein forms ringlike complexes with the major nucleolar protein, fibrillarin, in vitro. J Mol Biol. 2008;376(4):932-7.

34. Kim SH, MacFarlane S, Kalinina NO, Rakitina DV, Ryabov EV, Gillespie T, et al. Interaction of a plant virus-encoded protein with the major nucleolar protein fibrillarin is required for systemic virus infection. Proceedings of the National Academy of Sciences. 2007;104(26):11115.

35. Kim SH, Ryabov EV, Kalinina NO, Rakitina DV, Gillespie T, MacFarlane S, et al. Cajal bodies and the nucleolus are required for a plant virus systemic infection. EMBO J. 2007;26(8):2169-79.

36. Kim SH, Macfarlane S, Kalinina NO, Rakitina DV, Ryabov EV, Gillespie T, et al. Interaction of a plant virus-encoded protein with the major nucleolar protein fibrillarin is required for systemic virus infection. Proc Natl Acad Sci U S A. 2007;104(26):11115-20.

37. Ryabov EV, Oparka KJ, Santa Cruz S, Robinson DJ, Taliansky ME. Intracellular location of two groundnut rosette umbravirus proteins delivered by PVX and TMV vectors. Virology. 1998;242(2):303-13.

38. Kalinina NO, Makarova S, Makhotenko A, Love AJ, Taliansky M. The Multiple Functions of the Nucleolus in Plant Development, Disease and Stress Responses. Frontiers in plant science. 2018;9(132).

39. Haupt S, Stroganova T, Ryabov E, Kim SH, Fraser G, Duncan G, et al. Nucleolar localization of potato leafroll virus capsid proteins. J Gen Virol. 2005;86(Pt 10):2891-6.

40. Chang C-H, Hsu F-C, Lee S-C, Lo Y-S, Wang J-D, Shaw J, et al. The Nucleolar Fibrillarin Protein Is Required for Helper Virus-Independent Long-Distance Trafficking of a Subviral Satellite RNA in Plants. Plant Cell. 2016;28(10):2586-602.

41. Feric M, Vaidya N, Harmon TS, Mitrea DM, Zhu L, Richardson TM, et al. Coexisting Liquid Phases Underlie Nucleolar Subcompartments. Cell. 2016;165(7):1686-97.

42. Taliansky M, Roberts IM, Kalinina N, Ryabov EV, Raj SK, Robinson DJ, et al. An umbraviral protein, involved in long-distance RNA movement, binds viral RNA and forms unique, protective ribonucleoprotein complexes. Journal of virology. 2003;77(5):3031-40.

43. May JP, Johnson PZ, Ilyas M, Gao F, Simon AE. The Multifunctional Long-Distance Movement Protein of Pea Enation Mosaic Virus 2 Protects Viral and Host Transcripts from Nonsense-Mediated Decay. mBio. 2020;11(2):e00204-20.

44. Ishikawa-Ankerhold H, Ankerhold, R. and Drummen, G. . Fluorescence Recovery After Photobleaching (FRAP). In eLS, John Wiley & Sons, Ltd (Ed)2014.

45. Dosztányi Z. Prediction of protein disorder based on IUPred. Protein Sci. 2018;27(1):331-40.

46. Bolognesi B, Lorenzo Gotor N, Dhar R, Cirillo D, Baldrighi M, Tartaglia GG, et al. A Concentration-Dependent Liquid Phase Separation Can Cause Toxicity upon Increased Protein Expression. Cell Rep. 2016;16(1):222-31.

47. Yang Y, Jones HB, Dao TP, Castañeda CA. Single Amino Acid Substitutions in Stickers, but Not Spacers, Substantially Alter UBQLN2 Phase Transitions and Dense Phase Material Properties. The Journal of Physical Chemistry B. 2019;123(17):3618-29.

48. S. KSMSA. Hexanediol: a chemical probe to investigate the material properties of membrane-less compartments. Matters [Internet]. Sciencematters; 2017 May 22; http://dx.doi.org/10.19185/matters.201702000010.

49. Luo H, Lee N, Wang X, Li Y, Schmelzer A, Hunter AK, et al. Liquid-liquid phase separation causes high turbidity and pressure during low pH elution process in Protein A chromatography. Journal of Chromatography A. 2017;1488:57-67.

50. Alberti S, Gladfelter A, Mittag T. Considerations and Challenges in Studying Liquid-Liquid Phase Separation and Biomolecular Condensates. Cell. 2019;176(3):419-34.

51. Wang J, Choi J-M, Holehouse AS, Lee HO, Zhang X, Jahnel M, et al. A Molecular Grammar Governing the Driving Forces for Phase Separation of Prion-like RNA Binding Proteins. Cell. 2018;174(3):688-99.e16.

52. Ryabov EV, Kim SH, Taliansky M. Identification of a nuclear localization signal and nuclear export signal of the umbraviral long-distance RNA movement protein. J Gen Virol. 2004;85(Pt 5):1329-33.

53. Hou Q, Bourgeas R, Pucci F, Rooman M. Computational analysis of the amino acid interactions that promote or decrease protein solubility. Scientific reports. 2018;8(1):14661.

54. Savada RP, Bonham-Smith PC. Charge versus sequence for nuclear/nucleolar localization of plant ribosomal proteins. Plant molecular biology. 2013;81(4-5):477-93.

55. Musinova YR, Kananykhina EY, Potashnikova DM, Lisitsyna OM, Sheval EV. A charge-dependent mechanism is responsible for the dynamic accumulation of proteins inside nucleoli. Biochimica et Biophysica Acta (BBA) - Molecular Cell Research. 2015;1853(1):101-10.

56. Frottin F, Schueder F, Tiwary S, Gupta R, Körner R, Schlichthaerle T, et al. The nucleolus functions as a phase-separated protein quality control compartment. Science. 2019:eaaw9157.

57. Rakitina DV, Taliansky M, Brown JWS, Kalinina NO. Two RNA-binding sites in plant fibrillarin provide interactions with various RNA substrates. Nucleic Acids Res. 2011;39(20):8869-80.

58. Yao RW, Xu G, Wang Y, Shan L, Luan PF, Wang Y, et al. Nascent Pre-rRNA Sorting via Phase Separation Drives the Assembly of Dense Fibrillar Components in the Human Nucleolus. Mol Cell. 2019;76(5):767-83.e11.

59. Berry J, Weber SC, Vaidya N, Haataja M, Brangwynne CP. RNA transcription modulates phase transition-driven nuclear body assembly. Proceedings of the National Academy of Sciences. 2015;112(38):E5237.

60. Banani SF, Rice AM, Peeples WB, Lin Y, Jain S, Parker R, et al. Compositional Control of Phase-Separated Cellular Bodies. Cell. 2016;166(3):651-63.

61. Ditlev JA, Case LB, Rosen MK. Who's In and Who's Out—Compositional Control of Biomolecular Condensates. Journal of Molecular Biology. 2018;430(23):4666-84.

62. Lindbo JA. TRBO: A High-Efficiency Tobacco Mosaic Virus RNA-Based Overexpression Vector. Plant Physiology. 2007;145(4):1232.

63. Ryabov EV, Robinson DJ, Taliansky ME. A plant virus-encoded protein facilitates long-distance movement of heterologous viral RNA. Proceedings of the National Academy of Sciences. 1999;96(4):1212-7.

64. Ryabov EV, Robinson DJ, Taliansky M. Umbravirus-encoded proteins both stabilize heterologous viral RNA and mediate its systemic movement in some plant species. Virology. 2001;288(2):391-400.

65. Krapp S, Greiner E, Amin B, Sonnewald U, Krenz B. The stress granule component G3BP is a novel interaction partner for the nuclear shuttle proteins of the nanovirus pea necrotic yellow dwarf virus and geminivirus abutilon mosaic virus. Virus Res. 2017;227:6-14.

66. Tourrière H, Chebli K, Zekri L, Courselaud B, Blanchard JM, Bertrand E, et al. The RasGAP-associated endoribonuclease G3BP assembles stress granules. J Cell Biol. 2003;160(6):823-31.

67. Guillén-Boixet J, Kopach A, Holehouse AS, Wittmann S, Jahnel M, Schlüßler R, et al. RNA-Induced Conformational Switching and Clustering of G3BP Drive Stress Granule Assembly by Condensation. Cell. 2020;181(2):346-61.e17.

68. Nevers Q, Albertini AA, Lagaudrière-Gesbert C, Gaudin Y. Negri bodies and other virus membrane-less replication compartments. Biochim Biophys Acta Mol Cell Res. 2020;1867(12):118831-.

69. Lahaye X, Vidy A, Pomier C, Obiang L, Harper F, Gaudin Y, et al. Functional characterization of Negri bodies (NBs) in rabies virus-infected cells: Evidence that NBs are sites of viral transcription and replication. Journal of virology. 2009;83(16):7948-58.

70. Guseva S, Milles S, Jensen MR, Salvi N, Kleman J-P, Maurin D, et al. Measles virus nucleo- and phosphoproteins form liquid-like phase-separated compartments that promote nucleocapsid assembly. Sci Adv. 2020;6(14):eaaz7095-eaaz.

71. Belov GA, van Kuppeveld FJ. (+)RNA viruses rewire cellular pathways to build replication organelles. Curr Opin Virol. 2012;2(6):740-7.

72. Nagy PD, Strating JR, van Kuppeveld FJ. Building Viral Replication Organelles: Close Encounters of the Membrane Types. PLoS Pathog. 2016;12(10):e1005912.

73. Alers-Velazquez R, Jacques S, Muller C, Boldt J, Schoelz J, Leisner S. Cauliflower mosaic virus P6 inclusion body formation: A dynamic and intricate process. Virology. 2021;553:9-22.

74. Li Q, Liu N, Liu Q, Zheng X, Lu L, Gao W, et al. DEAD-box helicases modulate dicing body formation in Arabidopsis. Sci Adv. 2021;7(18).

75. Yang Z, Li Y. Dissection of RNAi-based antiviral immunity in plants. Current opinion in virology. 2018;32:88-99.

76. Boeynaems S, Bogaert E, Kovacs D, Konijnenberg A, Timmerman E, Volkov A, et al. Phase Separation of C9orf72 Dipeptide Repeats Perturbs Stress Granule Dynamics. Mol Cell. 2017;65(6):1044-55.e5.

77. Brangwynne Clifford P, Tompa P, Pappu Rohit V. Polymer physics of intracellular phase transitions. Nature Physics. 2015;11(11):899-904.

78. Reuper H, Amari K, Krenz B. Analyzing the G3BP-like gene family of Arabidopsis thaliana in early turnip mosaic virus infection. Scientific reports. 2021;11(1):2187.

79. Fischer JW, Busa VF, Shao Y, Leung AKL. Structure-Mediated RNA Decay by UPF1 and G3BP1. Mol Cell. 2020;78(1):70-84.e6.

80. Simon AE, Miller WA. 3' cap-independent translation enhancers of plant viruses. Annual review of microbiology. 2013;67:21-42.

81. May JP, Yuan X, Sawicki E, Simon AE. RNA virus evasion of nonsense-mediated decay. PLoS Pathog. 2018;14(11):e1007459.

82. May JP, Simon AE. Targeting of viral RNAs by Upf1-mediated RNA decay pathways. Current opinion in virology. 2020;47:1-8.

83. Brown JAL, Roberts TL, Richards R, Woods R, Birrell G, Lim YC, et al. A novel role for hSMG-1 in stress granule formation. Mol Cell Biol. 2011;31(22):4417-29.

84. Kertész S, Kerényi Z, Mérai Z, Bartos I, Pálfy T, Barta E, et al. Both introns and long 3'-UTRs operate as cis-acting elements to trigger nonsense-mediated decay in plants. Nucleic Acids Res. 2006;34(21):6147-57.

85. Mérai Z, Kerényi Z, Molnár A, Barta E, Válóczi A, Bisztray G, et al. Aureusvirus P14 is an efficient RNA silencing suppressor that binds double-stranded RNAs without size specificity. Journal of virology. 2005;79(11):7217-26.

86. Boeynaems S, De Decker M, Tompa P, Van Den Bosch L. Arginine-rich Peptides Can Actively Mediate Liquid-liquid Phase Separation. Bio-protocol. 2017;7(17):e2525.

87. Busch DJ, Houser JR, Hayden CC, Sherman MB, Lafer EM, Stachowiak JC. Intrinsically disordered proteins drive membrane curvature. Nature communications. 2015;6:7875.

88. DeGroot ACM, Busch DJ, Hayden CC, Mihelic SA, Alpar AT, Behar M, et al. Entropic Control of Receptor Recycling Using Engineered Ligands. Biophysical journal. 2018;114(6):1377-88.

89. Stauffer W, Sheng H, Lim HN. EzColocalization: An ImageJ plugin for visualizing and measuring colocalization in cells and organisms. Scientific reports. 2018;8(1):15764.

Formatted: Line spacing: Double

FIGURE LEGENDS

Fig. 1. **p26 forms poorly dynamic condensates *in vivo***. (A) PEMV2 is a small positive-sense RNA plant virus that encodes 4 genes, including the p26 long-distance movement protein. Free GFP and p26 C-terminally fused with GFP (p26:GFP) were expressed from binary expression plasmids under the constitutive CaMV 35S promoter (B) Following agroinfiltration of *N. benthamiana,* confocal microscopy showed diffuse cytoplasmic and nuclear expression of free GFP whereas p26:GFP formed large cytoplasmic bodies. Note that the majority of plant mesophyll cells is taken up by a single large vacuole. Differential interference contrast (DIC) microscopy was used for p26:GFP samples to visualize cell borders. Bar scale: 20 µm. (C) FRAP analysis of p26:GFP was performed by photobleaching cytoplasmic condensates and monitoring fluorescence recovery at 5 s intervals. A representative p26:GFP condensate is shown before photobleaching, immediately following photobleaching (5 s), and at 120 s. Bar scale 5 µm. Average FRAP intensity is shown from seven FRAP experiments and shaded area represents 95% confidence interval.

Fig. 2. **p26 is intrinsically disordered and phase separates through electrostatic interactions**. (A) (Top) The IUPRED algorithm [45] predicts that PEMV2 p26 contains a large intrinsically disordered region (IDR) spanning amino acids 1-132. (Bottom) ~~.~~The same region has the highest predicted phase separation propensity using the catGRANULE algorithm [46]. ~~The dispensable cell-to-cell movement protein, p27, is highly ordered.~~ (B) The p26 IDR was fused to the N-terminus of GFP for bacterial expression and contained an N-terminal histidine tag. The p26 IDR sequence is shown with highlighted residues corresponding to basic (blue) or acidic (red) residues. The conserved nuclear localization signal (NLS) is highlighted in yellow. (C) Recombinant proteins used in this study were analyzed by SDS-PAGE to assess size and purity. Proteins were stained using Coomassie Blue. Marker (M) sizes are shown in kilodaltons (kDa). R/K-G and D/E-G IDR mutants contain glycine substitutions for all basic or acidic IDR

residues, respectively. ΔNLS is missing the nuclear localization signal 5’-RRRARR-3’ (amino acids 100-105) within the IDR. Note: R/K-G ran markedly higher both *in vitro* and *in vivo* (see Fig. 4B6B). (D) Molecular crowding was induced with 10% PEG in the presence of 24 µM free GFP or IDR-GFP. The IDR-GFP solution became turbid in the presence of PEG, indicative of phase separation. (E) *In vitro* phase separation assays were visualized by confocal microscopy. 8 µM protein was used for all assays and 10% PEG-8000 was added as a crowding agent (Middle panels). One molar NaCl was added to disrupt electrostatic interactions (Right panel). 10% 1,6 hexanediol was added to IDR-GFP phase separations to assess the fluidity of condensates. Bar scale: 20 µm. (E) Turbidity assays (OD_{600}) using either 8 µM or 24 µM protein were performed for all constructs. Only IDR-ΔNLS turbidity was not significantly reduced compared to IDR-GFP. **** *P*<0.0001 by two-way ANOVA with Dunnett’s multiple comparisons test vs. IDR-GFP. (F) Phase diagram for IDR-GFP gives an apparent C_{sat} = 4 2 µM and sensitivity to high NaCl concentrations. Results are representative of two independent experiments. (G)) IDR mutants (8 µM) were examined using *in vitro* phase separation assays. R/K-G formed irregular aggregates at high concentration (24 µM) and D/E-G showed reduced phase separation compared to IDR-GFP. R-K and VLIMFYW-S mutants appeared like wild-type IDR. Bar scale: 20 µm (H) D/E-G had significantly reduced turbidity (OD_{600}) under crowding conditions when compared to IDR-GFP at 8 µM and 24 µM concentrations. Data represents three independent replicates for each condition. Bars denote standard deviations. *** *P*<0.001 unpaired t test (I) Mean condensate sizes for all mutants (excluding R/K-G) were plotted by cumulative distribution frequency. Particle sizes were measured from three representative 20x fields using ImageJ. *P* values represent results from two-tailed Mann-Whitney tests compared to IDR-GFP. ns: not significant. (J) FRAP was performed for *in vitro* condensates. 24 µM protein was used for R/K-G and D/E-G. Inset shows representative IDR-GFP and D/E-G droplets, or R/K-G aggregates. Bar scale: 10 µm. Table shows %recovery after 2 minutes with Mann-

Formatted: Subscript

Whitney rank test comparisons against IDR-GFP. Data represents 7-10 separate FRAP measurements for each mutant. Shaded areas represent 95% confidence intervalsstandard deviations.

Fig. 3. **Charged residues govern p26 nucleolar partitioning**. **Phase separation supports p26 partitioning in Fib2 droplets and vRNP formation**. (A) p26:GFP, R/K-G, and D/E-G GFP fusions were expressed from the CaMV 35S promoter in *N. benthamiana* leaves following agroinfiltration. Prior to imaging, leaves were infiltrated with 5 µg/mL DAPI to stain nuclei. 20x and 63x fields are shown. Arrows denote p26 partitioned inside the nucleolus Nuclear Bodies (NBs)(No) or cajal bodies (CB). Bar scale: Top 20 µm; Bottom 10 µm. (B) Nuclear localization of p26:GFP or D/E-G was quantified using Mander's overlap coefficient (MOC) using ImageJ and EzColocalizationNuclear granules were manually counted from six 20x fields. Total granule counts were calculated using the ImageJ "analyze particles" toolg EzColocalization [89]. White outlines represent thresholded nuclei. Representative results are from ten 20x fields. Bar scale: 50 µm. Error bars denote standard deviations. ****P<0.0001 unpaired t test. (C) Fib2 contains an N-terminal glycine- and arginine-rich (GAR) domain that is intrinsically disordered. (D) Either the Fib2 GAR domain (Fib2$_{GAR}$) or full-length Fib2 (Fib2$_{FL}$) were fused to mCherry and purified from *E. coli* and analyzed by SDS-PAGE. Molecular weight (kDa) marker is shown. (E) mCherry, Fib2$_{GAR}$, and Fib2$_{FL}$ were examined by confocal microscopy after inducing phase separation with 10% PEG-8000 alone or in the presence of 1 M NaCl. 8 µM protein was used for all assays. Bar scale: 20 µm. (F) FRAP analyses of Fib2$_{GAR}$ and Fib2$_{FL}$ condensates. Shaded areas represent 95% confidence intervals. Results are from 8 separate FRAP experiments. Table shows %recovery after two minutes. **** P<0.0001 Mann-Whitney rank test comparison (G) Fib2$_{GAR}$ and Fib2$_{FL}$ droplets were pre-formed prior to addition of PEMV2-Cy5 at a 500:1 protein:RNA molar ratio. PEMV2 RNA was only sorted to Fib2$_{FL}$ condensates. Bar scale: 20 µm. (H) IDR-GFP droplets were pre-formed prior to addition of PEMV2-Cy5 or TCV-Cy5 at a 500:1

protein:RNA molar ratio. Bar scale: 20 µm. (I) The fraction of IDR-GFP signal that was positive for Cy5-labelled RNA was determined by MOC analysis using EzColocalization [89]. ns: not significant by unpaired t test. Bars denote standard deviations. Three 20x fields were quantified for each condition. (J) $Fib2_{GAR}$ droplets were pre-formed using 24 µM protein before the addition of 4 µM IDR-GFP or R/K-G. Sorting of IDR-GFP to Fib2 droplets was observed whereas R/K-G remained in the bulk phase and failed to partition in $Fib2_{GAR}$ droplets. Bar scale 10 µm. (K) IDR-GFP, $Fib2_{FL}$, and PEMV2-Cy5 RNA were mixed at a 500:500:1 molar ratio after pre-forming $Fib2_{FL}$ and IDR-GFP condensates under crowding conditions. Droplets containing all components were observed. Bar scale: 10 µm. Images in all panels are representative of at least three independent experiments.

Fig 4. p26 phase separation is required for partitioning in Fib2 droplets. (A) Fib2 contains an N-terminal glycine- and arginine-rich (GAR) domain that is intrinsically disordered. (B) Either the Fib2 GAR domain ($Fib2_{GAR}$) or full-length Fib2 ($Fib2_{FL}$) were fused to mCherry and purified from *E. coli* and analyzed by SDS-PAGE. Molecular weight (kDa) marker is shown. (C) mCherry, $Fib2_{GAR}$, and $Fib2_{FL}$ were examined by confocal microscopy after inducing phase separation with 10% PEG-8000 alone or in the presence of 1 M NaCl. 8 µM protein was used for all assays. Bar scale: 20 µm. (D) $Fib2_{GAR}$ droplets were pre-formed using 24 µM protein before the addition of 4 µM IDR-GFP or R/K-G. Sorting of IDR-GFP to Fib2 droplets was observed whereas R/K-G remained in the bulk phase and failed to partition in $Fib2_{GAR}$ droplets (White arrows). Bar scale 10 µm.

Formatted: Font: Bold

Fig. 5. vRNPs required for systemic trafficking can be reconstituted *in vitro* via phase separation. (A) $Fib2_{GAR}$ and $Fib2_{FL}$ droplets were pre-formed prior to the addition of PEMV2-Cy5 RNAs at a 1:500 RNA:protein molar ratio. PEMV2 RNA was only efficiently sorted to $Fib2_{FL}$ condensates. Bar scale: 20 µm. (B) IDR-GFP droplets were pre-formed prior to the addition of

PEMV2-Cy5, TCV-Cy5, or RLuc-Cy5 RNAs at a 1:500 RNA:protein molar ratio. Bar scale: 20 µm. (C) The fraction of IDR-GFP signal that was positive for Cy5-labelled RNA was determined by MOC analysis using EzColocalization [89]. ns: not significant by unpaired t test. Bars denote standard deviations. Three 20x fields were quantified for each condition. (D) IDR-GFP, Fib2$_{FL}$, and PEMV2-Cy5 RNA were mixed at a 500:500:1 molar ratio after pre-forming Fib2$_{FL}$ and IDR-GFP condensates under crowding conditions. Droplets containing all components were observed. Bar scale: 10 µm. Images in all panels are representative of at least threetwo independent experiments.

Formatted: Font: Bold

Fig. 46. Phase separation-deficient p26 mutants fail to systemically traffic a virus vector. (A) pJL-TRBO TMV vector lacks coat protein (CP) and is severely impaired in systemic trafficking. Free GFP, p26:GFP, R/K-G, and D/E-G GFP fusions were inserted into pJL-TRBO to test whether systemic trafficking could be restored. (B) Following agroinfiltration of *N. benthamiana* leaves, TMV infections were established in local leaves. Free GFP, or GFP-fusion proteins were visualized and detected in local leaves at 4 dpi by UV exposure (Left) or western blotting (Right). Rubisco serves as a loading control. Red asterisks denote free GFP or GFP-fusion bands. (C) Localization patterns in TMV-infected leaves confirmed that neither free GFP or R/K-G form phase separated granules. Bar scale: 20 µm. Nuclear p26:GFP or D/E-G granules were counted from 5 20x fields of view and divided by the total number of granules (counted with ImageJ) to calculate a percentage (%). The fraction of D/E-G nuclear granules was significantly higher than observed for wild-type. Expression patterns did not differ between 35S-driven or TMV-expressed p26:GFP or D/E-G. 35S promoter data from Fig. 3B was included for comparison. (D) At 14 dpi, systemic leaves were imaged prior to total RNA extraction. RT-PCR was used to amplify 100-200 bp fragments targeting either the TMV replicase or actin as a control. -RT: No reverse transcriptase controls. Two pools of 3-4 leaves are shown for each

construct. Results are representative of three independent experiments consisting of at least 4 plants/construct.

Fig. ~~5~~7. p26 is sorted into G3BP phase separations that restrict PEMV2 accumulation. (A) *A. thaliana* G3BP contains an ordered NTF2 domain and RNA recognition motif (RRM) in addition to intrinsically disordered regions. (B) G3BP:RFP or ΔNTF2-G3BP:RFP were expressed from CaMV 35S promoters following agroinfiltration of ~~were agroinfiltrated into~~ *N. benthamiana* leaves. At 3 dpi, plants were either imaged directly or heat shocked for 45 minutes at 37°C. p26:GFP was co-infiltrated with G3BP:RFP and p26 partitioning in G3BP SGs was observed (White arrows). Scale bar: 20 µm. Inset shows western blot using anti-RFP antibodies to detect full-length G3BP and ΔNTF2-G3BP. Rubisco was used as a loading control (C) G3BP:RFP was agroinfiltrated into ~~systemically-infected TMV:p26:GFP~~ ~~*plants*~~ *N. benthamiana* plants systemically infected with TMV (pJL-TRBO) expressing p26:GFP. Confocal microscopy was used to observe co-localization (White arrows) between p26 and G3BP during virus infection~~to determine if p26 partitions in G3BP SGs during a virus infection~~. ~~p26:GFP co-localized with G3BP SGs as labelled by white arrows.~~ Scale bar: 20 µm. (D) Native G3BP expression was measured in Mock- or PEMV2-infected *N. benthamiana* at 3 dpi by RT-qPCR. The ~~co-~~agroinfiltrated p14 RNA silencing suppressor was used as a reference gene. Data is from three biological replicates. **P*<0.05; student's t-test. Bars denote standard error. (E) PEMV2 was agroinfiltrated alone, or alongside either G3BP or ΔNTF2-G3BP (both tagged with RFP). At 3 dpi, total protein and total RNA~~s~~ ~~were~~ was extracted and used for western blot~~s~~ or RT-qPCR targeting PEMV2 or p14 (reference gene), respectively. Full-length G3BP and ΔNTF2 accumulated to similar levels when detected by anti-RFP antibody (top). RT-qPCR r~~R~~esults ~~shown~~ ~~are from~~represent 7 biological replicates from 2 independent experiments. Bars denote

Formatted: Font: Italic

standard error. Brown-Forsythe and Welch ANOVA with multiple comparisons was used to determine if observed differences were significant. ** P<0.01.

Formatted: Space After: 0 pt
