# Supplementary material for: Phase separation of a plant virus movement protein and cellular factors support virus-host interactions
Source: PLoS Pathog. 2021 Sep 20;17(9):e1009622. doi: 10.1371/journal.ppat.1009622 (PMC8483311; doi:10.1371/journal.ppat.1009622)
Supplement: Attachment — Submitted filename: Response_to_reviewers.docx [file ppat.1009622.s010.docx]

Reviewer #1: the study is novel, interesting, and significant. However the paper is very badly written. The M&M is very much of a mess. The results are circular arguments.

We would like to thank the reviewer for their positive assessment of our work. We would also like to thank the reviewer for their constructive feedback which has significantly improved the writing and clarity of the text. The M&M section has been completely reorganized and expanded (see below). We have realized how the excessive use of the term “phase separation” created many instances where circular arguments and jargon were present in the text. The revised version has 86 instances of “phase separate” versus 158 instances in the previous version. Circular arguments have been removed from the text. Please see the supplied annotated PDF for responses to all writing-related issues that were raised.

In M&M I learned that a silencing suppressor was used throughout the study and so that should be explained in the results.

The p14 was included in all agroinfiltrations to increase transient gene expression, as is common for agroinfiltration protocols. In the results on lines 114-116, the following sentence has been added to the results:

“To maximize transient gene expression, the p14 RNA silencing suppressor from Pothos latent virus [50] was included in all infiltrations.”

Later in the results on lines 277-278, the inclusion of p14 was reiterated to remind readers that p14 was included in the PEMV2 + G3BP agroinfiltrations. When assessing the inhibitory effects of G3BP on PEMV2 RNA accumulation, including the p14 silencing suppressor is beneficial for two reasons:

1. PEMV2 RNA accumulates to much higher levels making it easier to detect antiviral activities of proteins expressed *in trans*
2. We can now conclude that G3BP inhibits PEMV2 RNA accumulation independently of RNA silencing, eliminating this possibility from potential mechanisms.

Finally, the use of p14 has been described in the M&M (Lines 395-397). Also, per the reviewers concern, we did not construct the p14 plasmid and the original source is referenced.

The results need to elaborate experimental design, how data is collected and analyzed. how many replicates were performed. Statistics. Conclusions need to go into the discussion.

Figure legends include the number of replicates and applicable statistics for all figures. Furthermore, all bar graphs include individual data points for all experiments. For image analyses, the number of fields is included in all figure legends and ranges from 3-6 fields of view. All microscopy images are representative of at least two independent experiments.

The reviewers concern regarding a lack of statistics for comparing confocal microscopy images has been addressed by calculating the “total droplet areas” from individual 20x fields throughout the manuscript. ImageJ was used to measure droplet areas from three 20x fields for each condition. Samples were then compared with t tests or ANOVA w/ multiple comparisons to determine significance. This has been done for Fig. 2, Fig. 4, S1 Fig, and S3 Fig. Circularity of droplets was analyzed using imageJ for S2 Fig. Additional MOC analyses are now included for comparing RNA sorting into Fib2GAR or Fib2FL droplets (Fig. 5B).

Regarding Fig. 1C. The reviewer stated that statistics should be included. However, no comparisons were being made, rather we were determining whether there was measurable fluidity in p26 droplets in vivo. We could use a one-sample t test to show that the recovery (50%) was significantly higher than zero, but this seems unnecessary and is not typical for FRAP assays from other studies. See below for examples:

Burke KA, Janke AM, Rhine CL, Fawzi NL. Residue-by-Residue View of In Vitro FUS Granules that Bind the C-Terminal Domain of RNA Polymerase II. Mol Cell. 2015;60(2):231-241. doi:10.1016/j.molcel.2015.09.006

Schmidt HB, Barreau A, Rohatgi R. Phase separation-deficient TDP43 remains functional in splicing. Nat Commun. 2019 Oct 25;10(1):4890. doi: 10.1038/s41467-019-12740-2. PMID: 31653829; PMCID: PMC6814767.

All statistical analyses are detailed in the M&M section titled “Statistical analyses.” The software used (GraphPad Prism) and version number is included. Also, ANOVA, unpaired t tests, multiple unpaired t tests, Mann-Whitney rank test, multiple comparisons test (Tukey, Dunnet, Sidaks) are detailed in this section. P value cutoffs are also included.

Several sentences have been moved from the results to the conclusions as suggested in the annotated PDF file.

The introduction and summary should highlight the viral basis for this study and stay away from neurodegenerative studies. the viral basis for this work is in the first paragraph of the discussion. I am providing an annotated pdf to help the authors rewrite.

All references to neurodegenerative diseases in the author summary and introduction have been removed. Per the reviewer’s suggestion, the first paragraph of the discussion containing the viral basis for the work has been moved to the introduction.

The cloning needs to be in one section of the M&M and PCR primers need to be explained and maybe put in a table. The M&M needs to provide sufficient information that others can repeat the work. Since the genes and mutants were synthesized the coding sequences need to be provided-otherwise no one can reproduce this work. gene accessions need to be provided.

In order to fully address the reviewer’s concerns, the following changes have been made as outlined in the annotated PDF supplied by the reviewer:

1. The Materials & Methods section has been completely overhauled. A new file “S1 Supplemental Information” outlines all cloning strategies with restriction sites, primer names, etc. S1 Supplemental Information also includes all nucleotide sequences used in this study, includes all protein amino acid sequences produced in this study, and includes all predicted molecular weights (used for molarity calculations). All primers used in this study are now included in a new file named “S1 Table.”
2. The text has been reorganized to keep all cloning details together, all agroinfiltration conditions are described together, and new paragraphs for “*Confocal microscopy and image processing”* and “*Statistical analyses*” are now included. All supplier information for critical reagents are now included.
3. Accession numbers have been added for G3BP and Fibrillarin when first introduced in the M&M. The PEMV2 genome used in this study is not found in NCBI databases. Therefore, the complete PEMV2 genome is included in S1 Supplemental Information. This sequence was used to amplify p26, C-term, and is also the genomic sequence in pCB-PEMV2. Coding sequences and amino acid sequences for all proteins are included in S1 Supplemental Information.

The discussion makes sweeping conclusions connecting in vitro data to systemic movement and this is a false argument.

We have changed the following sentence from the previous version “These observations suggest that p26 phase separation is required for systemic movement since p26 likely encounters pre-formed Fib2 droplets when first entering the nucleolus during infection.”

to

“Unlike IDR_WT_, IDR_R/K-G_ remained in the bulk-phase and was excluded from Fib2 droplets suggesting that the ability of p26 to assemble in a dense phase is important for interacting with Fib2.” Lines 294-296

Now our in vitro data only suggests that phase separation is important for interactions with Fib2, not virus movement. However, since Fib2-p26 interactions are necessary for movement, our results suggest phase separation could be involved. However, we agree that it is best to remove this conclusion from the text and allow the reader to draw their own conclusions.

Reviewer #2: The authors have fully addressed my concerns/comments. It could be beneficial to general readers if the authors include their explanation "PEMV2 will not tolerate addition of a fluorescent reporter" to methods or discussions during the publication process. Other than that, I recommend publishing this manuscript.

We would like to thank the reviewer for their insight throughout the peer review process. Introduction of the R/K-G or D/E-G mutations into a full-length PEMV2 clone would most likely disrupt RNA replication since p26 is required for replication in single cells (i.e. independently of virus movement). References have been added to the text to support these claims. Using a system like TMV was critical to only assess the effects of the R/K-G and D/E-G mutations on virus movement, and not virus RNA replication. Had PEMV2 been used, RNA replication in the R/K-G and D/E-G viruses would have likely reduced RNA replication making it impossible to accurately assess the effects on virus movement. Therefore, we have included the following sentences to the results section to describe why we used a TMV vector to assay virus movement over PEMV2 with a fluorescent reporter.

Lines 232-236: “Since p26 protects PEMV2 subgenomic RNA from nonsense-mediated decay [42], it is required for efficient PEMV2 RNA replication in single cells [65]. Thus, it is difficult to distinguish the effects of p26 mutations on RNA replication and virus movement. To separate these two functions, we assayed if p26 mutants could support movement of a heterologous viral RNA in N. benthamiana.”
